# The systematics of the Cervidae: a total evidence approach

**DOI:** 10.7717/peerj.8114

**Published:** 2020-02-18

**Authors:** Nicola S. Heckeberg

**Affiliations:** 1 Museum für Naturkunde Berlin, Leibniz Institute for Evolution and Biodiversity Science, Berlin, Germany; 2 SNSB-Bayerische Staatssammlung für Paläontologie und Geologie, Munich, Germany; 3 Department of Earth and Environmental Sciences, Palaeontology & Geobiology, Ludwig-Maximilians-Universität München, Berlin, Germany

**Keywords:** Evolutionary history, Ruminantia, Morphology, Phylogeny, Combined analyses

## Abstract

Systematic relationships of cervids have been controversial for decades. Despite new input from molecular systematics, consensus could only be partially reached. The initial, gross (sub) classification based on morphology and comparative anatomy was mostly supported by molecular data. The rich fossil record of cervids has never been extensively tested in phylogenetic frameworks concerning potential systematic relationships of fossil cervids to extant cervids. The aim of this work was to investigate the systematic relationships of extant and fossil cervids using molecular and morphological characters and make implications about their evolutionary history based on the phylogenetic reconstructions. To achieve these objectives, molecular data were compiled consisting of five nuclear markers and the complete mitochondrial genome of 50 extant and one fossil cervids. Several analyses using different data partitions, taxon sampling, partitioning schemes, and optimality criteria were undertaken. In addition, the most extensive morphological character matrix for such a broad cervid taxon sampling was compiled including 168 cranial and dental characters of 41 extant and 29 fossil cervids. The morphological and molecular data were analysed in a combined approach and other comprehensive phylogenetic reconstructions. The results showed that most Miocene cervids were more closely related to each other than to any other cervids. They were often positioned between the outgroup and all other cervids or as the sister taxon to Muntiacini. Two Miocene cervids were frequently placed within Muntiacini. Plio- and Pleistocene cervids could often be affiliated to Cervini, Odocoileini or Capreolini. The phylogenetic analyses provide new insights into the evolutionary history of cervids. Several fossil cervids could be successfully related to living representatives, confirming previously assumed affiliations based on comparative morphology and introducing new hypotheses. New systematic relationships were observed, some uncertainties persisted and resolving systematics within certain taxa remained challenging.

## Introduction

Cervidae (deer) belong to Ruminantia together with Tragulidae (chevrotains), Antilocapridae (pronghorns), Moschidae (musk deer), Giraffidae (giraffes) and Bovidae (cattle, sheep, antelopes). Cervids are the second most diverse group of ruminants and are natively distributed in the Americas, Europe and Asia inhabiting a broad variety of habitats. Apart from the recent dispersal and radiation into South America, cervids are mainly restricted to the Northern Hemisphere (Geist, 1998; Gentry, 2000; Scott & Janis, 1987; Webb, 2000).

Despite all efforts to resolve cervid (and ruminant) systematics over the past decades, there is only partial consensus from the phylogenetic reconstructions and several problems persist. Controversial species delimitations, unknown taxon affiliation, contradictory information from the data, and/or incomplete phylogenetic reconstruction were specified as possible reasons for these problems. To solve phylogenetic relationships of cervids (and ruminants), however, is of considerable interest, because of their important biological and economic role as wild and domestic animals (Cronin, 1991; Randi et al., 2001; Price, Bininda-Emonds & Gittleman, 2005).

In contrast to early systematic studies, which were often based only on morphological characters (Gentry, Rössner & Heizmann, 1999), there are now numerous molecular approaches (Hassanin et al., 2012) and a few supertree studies (Price, Bininda-Emonds & Gittleman, 2005) reconstructing cervid systematics. However, combined or total evidence (TE) approaches are still scarce (Groves & Grubb, 1987; Groves, 2014). Although the fossil record for cervids is good, systematic relationships of fossil cervids are even more uncertain than those of extant cervids. There are numerous qualitative descriptions and comparative morphological studies for fossil cervids, but there are only very few phylogenetic approaches on fossil taxa. While these were mainly based on antler characters, Mennecart et al. (2016, 2017) presented the first phylogenetic reconstructions of Miocene cervids based on inner ear morphology.

Various hypotheses of the intra-cervid systematic relationships have been published in the last decades. While in earlier studies up to six subfamilies of Cervidae have been recognised (Ouithavon et al., 2009), the family Cervidae now is usually classified into two subfamilies, Cervinae, consisting of Muntiacini and Cervini and Capreolinae, consisting of Alceini, Capreolini, Odocoileini and Rangiferini (Groves & Grubb, 1990; Miyamoto, Kraus & Ryder, 1990; Cronin et al., 1996; Randi et al., 1998, 2001; Hassanin & Douzery, 2003; Kuznetsova, Kholodova & Danilkin, 2005; Price, Bininda-Emonds & Gittleman, 2005; Gilbert, Ropiquet & Hassanin, 2006; Hughes et al., 2006; Ouithavon et al., 2009; Hassanin et al., 2012; Heckeberg et al., 2016; Gutiérrez et al., 2017). This classification is supported by classical morphological concepts and molecular evidence. In some studies Muntiacini is considered as a subfamily (Cronin et al., 1996; Randi et al., 1998; Kuznetsova, Kholodova & Danilkin, 2005; Marcot, 2007). While the systematic relationships within Muntiacini and Cervini are resolved, with very few exceptions, systematic relationships within Capreolinae are much more controversial. The position of Capreolini and Alceini is uncertain and there are many polyphylies within Odocoileini (Heckeberg et al., 2016; Gutiérrez et al., 2017). The latter is the youngest clade of cervids and diversified quickly after entering South America around 2.5 million years ago (mya), which makes resolving the systematic relationships more difficult.

**Figure 1:**
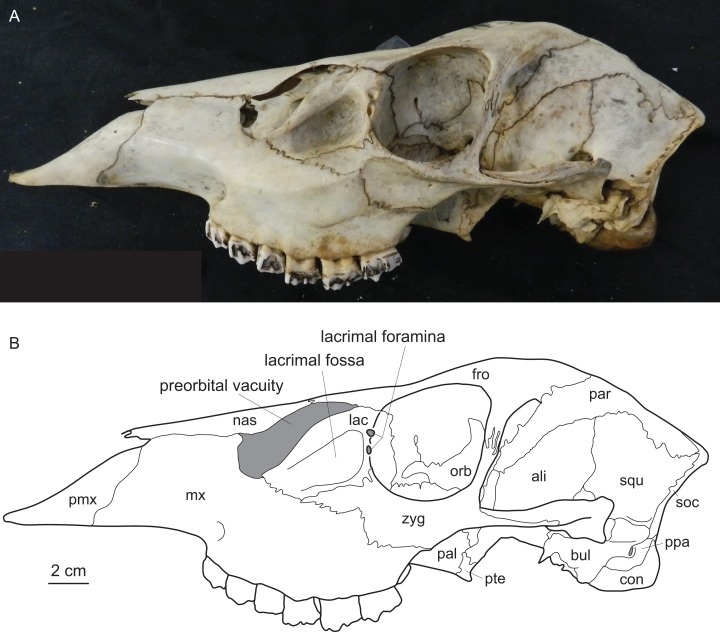
Diagnostic cranial characters of cervids. The most important diagnostic cranial features of cervids, with the exception of antlers, which almost exclusively occur in males, are outlined in this figure as (A) a photograph and (B) a drawing of the cranium of a female *Blastocerus dichotomus* (MNHN 1933-207). Note the brachyodont dentition, the preorbital vacuity, lacrimal fossa and lacrimal foramina. (Drawing by Nicola Heckeberg) pmx, premaxillary; mx, maxillary; nas, nasal; lac, lacrimal; zyg, zygomaticum; pal, palatine; pte, pterygoid; orb, orbitosphenoid; fro, frontal; par, parietal; ali, alisphenoid; squ, squamosal; soc, supraoccipital; ppa, paroccipital processes; bul, auditory bulla; con, condyles.

Diagnostic characters of cervids include, most importantly, the presence of antlers (Heckeberg, 2017b) and for example the presence of two lacrimal foramina, a lacrimal fossa, a preorbital vacuity and brachyodont dentition (Fig. 1; Janis & Scott (1987, 1988), Bouvrain, Geraads & Jehenne (1989) and Mickoleit (2004)). The first classification based on morphological characters split Cervidae into Telemetacarpi and Plesiometacarpi, which is equivalent to the Cervinae–Capreolinae split (Brooke, 1878). This split into Capreolinae and Cervinae was also confirmed by behavioural characters (Cap, Aulagnier & Deleporte, 2002; Groves, 2007). Further subdivision solely based on morphological features is difficult, because most cervid characters are highly conservative, partly phylogenetically uninformative and/or prone to convergence because of ecological adaptation (Groves & Grubb, 1987; Janis & Scott, 1987; Lister, 1996; Wada, Nishibori & Yokohama, 2007). However, there are a few morphological characters diagnosing cervid subclades (Bouvrain, Geraads & Jehenne, 1989; Cronin, 1991).

With molecular data outweighing morphological characters, morphology became less important in phylogenetic reconstructions (Huelsenbeck & Rannala, 2000). Discrepancies between morphological and molecular studies on ruminants demonstrated the need to continue combining fossil and extant species in order to reconstruct accurate phylogenies and to understand macro-evolutionary processes, which should yield better estimates than individual analyses (Hillis & Wiens, 2000; Hernández Fernández & Vrba, 2005). Several studies show the benefit of combining molecular and morphological data of fossil and living taxa in a TE analyses (Asher, 2007; De Queiroz & Gatesy, 2007; Geisler et al., 2011; Bibi, Vrba & Fack, 2012; Bibi, 2014). Complete species-level taxon sampling and extensive data sampling are required to reconstruct the ecological, biological and geographical patterns of cervid and ruminant evolutionary history (Price, Bininda-Emonds & Gittleman, 2005).

The aim of this work is to investigate the strength of morphological characters to reconstruct a cervid phylogeny, the systematic position of fossil cervids, and the influence of data partitioning and varying taxon sampling on the phylogenetic signal. To achieve this, compilation of the so far most extensive data set in terms of taxon and data sampling across Cervidae was necessary, including cranial and dental characters and five nuclear markers and the mitochondrial genome (mtG). In total, 79 fossil and living cervids were incorporated covering their entire evolutionary history from the early Miocene until today. With several analyses on different partitions and data combinations, analysing fossil and extant taxa separately and together, and under different optimality criteria, the systematic relationships of cervids were investigated. Phylogenetic hypotheses of fossil cervids in particular are tested with additional analyses including only one fossil at a time and the Fossil Placement Algorithm (FPA) approach (Berger & Stamatakis, 2010; Berger, Krompass & Stamatakis, 2011). The results provide new and intriguing insights into how fossil cervids are related to extant cervids.

## Methods

### Analyses of morphological data

#### Morphological data

**Figure 2:**
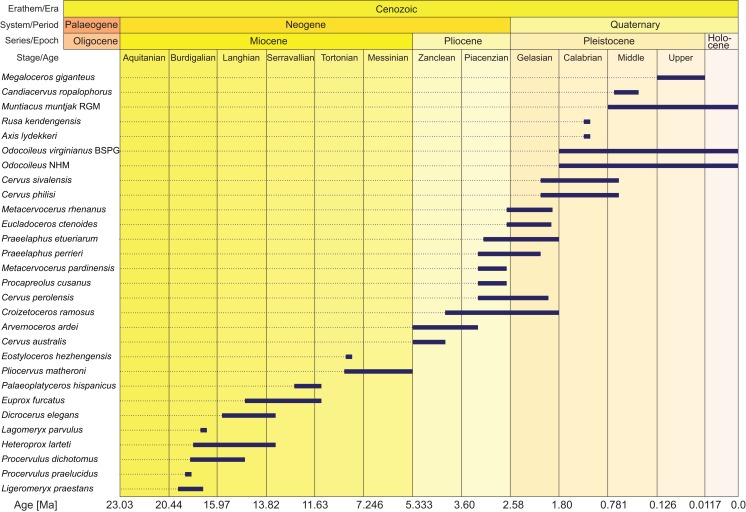
Age ranges of fossil cervids. Fossil cervids are arranged from the youngest first appearance datum to the oldest first appearance datum (left/top). The stage column widths are not to scale with time. The dates were compiled from the literature (Gentry, Rössner & Heizmann, 1999; Steininger, 1999; Böhme et al., 2012; Hilgen et al., 2012; Cohen et al., 2013; Croitor, 2014) and databases (NOW: www.helsinki/science/now/, PBDB: www.paleobiodb.org).

Morphological data were collected from 78 taxa, 41 extant and 29 fossil cervids, six non-cervid extant ruminant species, and two fossil non-cervid ruminants. Extant cervids were studied on 232 specimens, fossil cervids were studied on 504 specimens (see Table S1 for complete specimen lists). Most of the fossil cervid taxa consisted of fragments of several individuals. The fossils ranged from the Miocene to the Holocene and their temporal ranges are shown in Fig. 2. Measurements of each specimen were taken with a digital calliper with an accuracy of 0.1 mm. Distances larger than 15 cm were measured with a measuring tape with an accuracy of 0.5 mm. The 42 measuring distances are in the Supplemental File S1, the measurements are in Table S2. Ratios of the measurements served as source for discrete quantitative characters for the morphological matrix.

In total 168 morphological characters, 79 dental and 89 cranial, were scored, of which 19 were discrete quantitative characters and 34 were suitable for ordering. The morphological characters contain seven partitions; upper post-canine dentition (*n* = 35), lower post-canine dentition (*n* = 39), upper canines and lower incisors and canines (*n* = 5), mandible (*n* = 7), neurocranium (*n* = 25), viscerocranium (*n* = 40), antlers and pedicles (*n* = 17). The dental character set included eight discrete quantitative characters and 11 characters were suitable for ordering (6–8, 14, 21, 32, 40, 51, 59, 64 and 72). The cranial character set included 17 discrete quantitative characters and 23 characters were suitable for ordering (2, 4, 5, 8–12, 14, 15, 17–20, 23, 61, 74–79 and 89). Previous studies served as source for the decision on ordering selected characters (Gentry & Hooker, 1988; Bärmann & Sánchez-Villagra, 2012). Postcranial data could not be included in this study and is expected to be more useful in resolving interfamilial than intrafamilial relationships.

The character matrices, character and character state descriptions are available on morphobank (http://morphobank.org/permalink/?P1021) and in the Supplemental Files (Data Sets S1–S3).

#### Model choice and partitioning

The best fit of model distribution and partitioning scheme of the morphological character sets was tested using the efficient stepping stone (ss) sampling (Xie et al., 2011). The Bayes Factor (BF) was calculated as the ratio of the marginal likelihood of one model to the marginal likelihood of the competing model; BFs can then be used as the relative evidence in the data that favours one hypothesis in that respect that it predicts the observed data better than the competing hypotheses (Xie et al., 2011).

To test the combined morphological data set for the most suitable partitioning scheme, ordering scheme (unordered vs. ordered), and model distribution choice (gamma vs. not gamma), ss analyses were undertaken. In total, five ss sampling analyses were undertaken. The first three analyses were used to determine the partitioning scheme, running one analysis with an unpartitioned, unordered data set with the Γ distribution, one with a minimal partitioning scheme, dividing the data set into a cranial and dental character set. The third analysis was run with the maximal possible partitioning scheme, dividing the data set into upper post-canine dentition, lower post-canine dentition, other dentition, mandible, viscerocranium, neurocranium and antler characters. Afterwards, the data set, applying the resulting partitioning scheme, was tested for the gamma (Γ) distribution (Yang, 1994) with a fourth and the fifth analysis tested whether character state ordering is favoured over unordered character states (see Supplemental File S2).

Each ss analysis was run for 21.5 million generations, with a diagnostic frequency of 1,000 and a sample frequency of 500 and had 40 steps in total. The general settings are the same as for a normal BI analysis with MrBayes (Ronquist et al., 2012). The initial burnin of samples and the additional burnin in each step of the ss sampling were discarded. The aforementioned importance distributions are called power posterior distributions and were sampled via the Metropolis Coupled Monte Carlo Markov Chain run (Ronquist et al., 2012). In MrBayes this parameter is called alpha and was left as the default setting of 0.4, because in empirical studies it was found that the accuracy is maximal with an alpha value between 0.3 and 0.5 (Ronquist et al., 2012). After completion of the ss analyses the BFs of the summary of the marginal likelihoods of all 40 steps were calculated and compared with each other to decide for the favoured hypothesis. The decision for one hypothesis over another was based on the BF.

For the likelihood-based analyses on the morphological data, the Mk model was used (Lewis, 2001), which assumes that the rate of change from one character state to another is equal to the rate of reversal, that is the model is symmetrical. This is similar to the parsimony optimality criterion applied using an unweighted transition matrix for characters (Wright, Lloyd & Hillis, 2016).

#### Standard phylogenetic analyses

**Figure 3:**
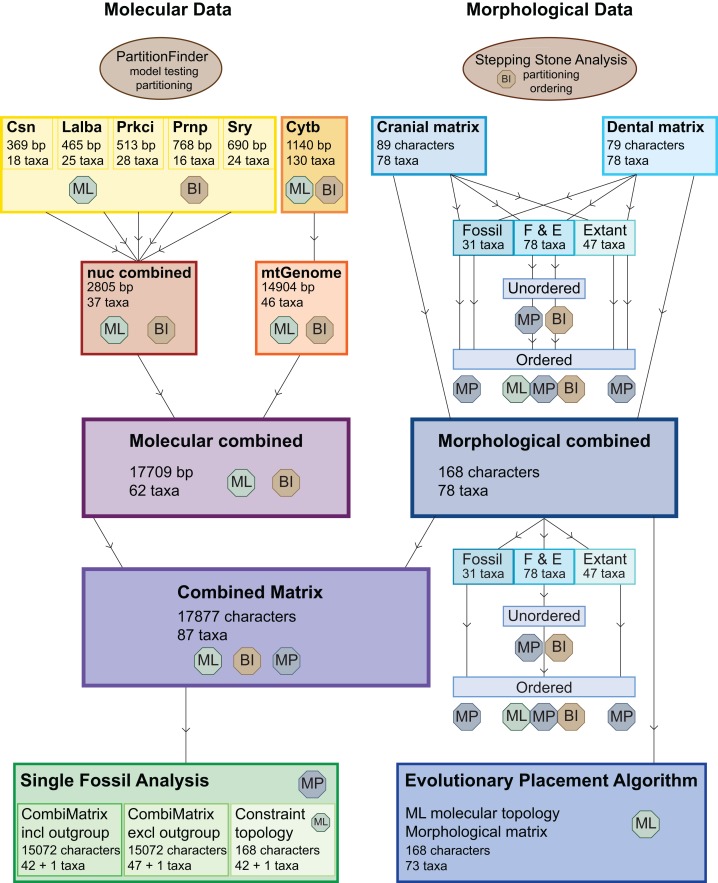
Overview of all analyses. This overview shows all analyses undertaken and the optimality criteria under which they were run. BI, Bayesian inference; ML, maximum likelihood; MP, maximum parsimony.

Figure 3 provides an overview of all (morphological and molecular) data sets and analyses undertaken. Tragulids were chosen as the outgroup for all analyses.

The dental, cranial and morphological combined data sets were analysed each with maximum parsimony (MP) with or without character ordering and varying taxon sampling, with Bayesian inference (BI) with and without character ordering, and with maximum likelihood (ML) without character ordering (Table 1; Fig. 3).

**Table 1:** Overview of all analyses undertaken.

Analysis ID	Opt. crit.	Data set	nchar	ntax
Dent_UnO_FE	MP	dental	79	78
Dent_O_FE	MP	dental	79	78
Dent_O_E	MP	dental	79	47
Dent_O_F	MP	dental	79	31
Dent_MB_UnO	BI	dental	79	78
Dent_MB_O	BI	dental	79	78
Dent_ML	ML	dental	79	78
Cran_UnO_FE^x^	MP	cranial	89	78
Cran_O_FE^x^	MP	cranial	89	78
Cran_O_E	MP	cranial	89	47
Cran_O_F^x^	MP	cranial	89	31
Cran_MB_UnO	BI	cranial	89	78
Cran_MB_O	BI	cranial	89	78
Cran_ML	ML	cranial	89	78
Combi_UnO_FE*	MP	morph. combined	168	78
Combi_O_FE*	MP	morph. combined	168	78
Combi_O_E	MP	morph. combined	168	47
Combi_O_F	MP	morph. combined	168	31
Combi_MB_UnO	BI	morph. combined	168	78
Combi_MB_O	BI	morph. combined	168	78
Combi_ML	ML	morph. combined	168	78
SFA_CombiMatrix^#^	MP	morph. mol. combined	15,072	48 (78)
SFA_CombiMatrix_noOut^#^	MP	morph. mol. combined	15,072	43 (73)
SFA_Backbone^#^	MP	morph. combined	168	43 (73)
FPA*	ML	morph. mol. combined	17,709 + 168	42 (73)
*Csn*	BI/ML	nuc molecular	369	20
*Lalba*	BI/ML	nuc molecular	465	25
*Prkci*	BI/ML	nuc molecular	513	29
*Prnp*	BI/ML	nuc molecular	768	21
*Sry*	BI/ML	nuc molecular	690	70
nucCombined	BI*/ML	nuc molecular	2,805	37
mtGenome	BI/ML	mt molecular	14,904	46
*Cytb*	BI/ML	mt molecular	1,140	130
mtCombined	BI*/ML	mt molecular	14,904	62
Molecular_Combined	BI*/ML	molecular combined	17,709	62
Mor_Mol_Combined*	BI/ML/MP	morph. mol. combined	17,877	87

**Notes:**

x Indicates analyses that were not successful, i.e. analyses which did not proceed in the tree search process after several attempts; this was most likely caused by a non-optimal proportion of characters and taxa.

* Indicates topologies that are figured in the main text.

# Only summarising topology figured in the main text; the topologies of all other analyses can be found in the Supplemental Material.

Dent, Dental; Cran, Cranial; Combi, Combined; UnO, unordered; O, ordered; E, Extant; F, Fossil; MP, maximum parsimony; BI, MB, Bayesian inference; ML, maximum likelihood; noOut, excluding most outgroup taxa; nuc, nuclear marker; mt, mitochondrial marker; Opt. Crit., Optimality Criterion; nchar, number of characters; ntax, number of taxa.

All MP analyses including bootstrap analyses were undertaken using PAUP* v.4.0b (Swofford, 2002). The analyses used a heuristic search running 1,000 replicates. Sequences were added randomly using the tree-bisection-reconnection (TBR) algorithm. Polymorphisms were treated as real polymorphisms. The strict consensus tree was calculated from all trees sampled in each analysis.

The BI analyses were undertaken using MrBayes 3.2.4 (Ronquist et al., 2012) under the Mk model (Lewis, 2001) and ran for 50 million generations with two runs à four chains at a temperature of 0.35; trees were sampled at every 5,000th generation until the standard deviation of split frequencies was below 0.01. A burnin of 0.25% was discarded after checking the convergence of the runs in Tracer v.1.6 (tree.bio.ed.ac.uk).

The ML analyses were undertaken using RAxML v.8.0.26 (Stamatakis, 2014). All ML analyses started at a random number seed and were run under the Mk model (Lewis, 2001) with the Γ model rate of heterogeneity without invariant sites. The analyses also included a rapid bootstrap search of 100 replicates starting at a random number seed.

#### Single fossil analyses

In order to reduce missing data in the data set, three sets of analyses were run, which included only one fossil taxon at a time. The three approaches to the SFA consisted each of 31 analyses including each one of the 31 fossil taxa. This adds up to 93 analyses in total. All SFA analyses were run using the PAUP* with the settings as specified above (Table 1). The first 31 analyses used the combined matrix of the complete mtG and the combined morphological data set (15,072 bp and characters) including outgroup taxa. In each of the 31 analyses 47 extant taxa and one fossil cervid were included. The second 31 analyses were undertaken using the same data set, but excluding five outgroup taxa. In each of the 31 analyses 42 extant taxa and one fossil cervid were included. *Hyemoschus aquaticus* was used to root the topologies. The third 31 analyses were undertaken based on the morphological character matrix and with a constraint topology as a backbone; The backbone topology was generated analysing the combined molecular data set including only those taxa, for which morphological data were available. Capreolinae, Muntiacini and Cervini were constraint as monophyletic polytomous to each other. In each of the third set of 31 analyses 42 extant taxa and one fossil cervid were included.

#### Fossil placement algorithm

Berger & Stamatakis (2010) and Berger, Krompass & Stamatakis (2011) introduced an algorithm implemented in RAxML, which improves accurate placement of morphology-based fossils in a tree. The FPA analysis is a two step process. The first step is a morphological weight calibration, where a molecular tree is provided alongside with the morphological matrix. All taxa have to entirely overlap in this step, therefore, only extant taxa were included. The second step invokes the actual FPA using the same molecular tree as in step one, the morphological matrix, including extant and fossil taxa, and the weight vector output from step one.

The molecular tree used here was specifically generated in RAxML based on a data set including only the 41 cervid species for which morphological data was available, 17,709 base pairs (nuc and mtDNA), and *Hyemoschus aquaticus* as outgroup. The morphological matrix for step one contained 42 species and 168 morphological characters (Table 1). In the second step of the FPA analysis, the same molecular tree was used, the morphological matrix now included extant and fossil cervids (73 in total), and the morphological weight vector from the first step was incorporated.

### Analyses of molecular data

#### Molecular data

Molecular data were compiled from GenBank (ncbi.nlm.nih.gov/genbank/). Five nuclear markers and the mtG were chosen for phylogenetic reconstructions based on their taxon sampling across cervids (*n* > 10). The GenBank accession numbers are in the Table S3.

The molecular data set included the nuclear non-coding markers, α-lactalbumin (*Lalba*), protein kinase C iota (*Prkci*), and the sex determining region on the Y-chromosome (*Sry*) and the nuclear coding markers κ-casein (*Csn*) and prion protein (*Prnp*) and the partially coding mtG. The coding markers were partitioned according to codon positions 1–3. Each gene was aligned using SeaView 4.2 (Gouy, Guindon & Gascuel, 2010) and Mesquite v.2.75 (Maddison & Maddison, 2011); alignments were carefully checked by eye for stop codons and/or unusual codon positions by translation into amino acids, where applicable, and were manually corrected if necessary. Some regions have been excluded from the alignment, for example the first and last couple of sites, which were not available for all taxa in the alignment (see Supplemental File S2).

Each nuclear gene was initially analysed separately, then all five nuclear genes were analysed in one matrix. The combined nuclear data set comprised 2,805 base pairs for 28 cervid species and nine non-cervid ruminant species (Table 1; Fig. 3). The complete mtG was available for 33 cervid species including 39 taxa and seven non-cervid ruminants, with a total of 14,904 base pairs Hassanin et al. (2012). The extensive *Cytb* data set from Heckeberg et al. (2016) was combined with the mtG. For the combined mtG–*Cytb* analyses, the original *Cytb* region of the mtG was replaced by the more taxon-rich *Cytb* alignment. The mitochondrial combined matrix included 51 cervid species across 56 cervid taxa and six non-cervid ruminants (Table 1). The combined molecular matrix consisted of 17,709 base pairs for 56 cervid taxa including 50 extant and 1 fossil cervids and six non-cervid ruminant species (Table 1).

#### Model choice

For each alignment PartitionFinder was used (Lanfear et al., 2012) to identify the appropriate substitution model and the optimal partitioning scheme. The Hasegawa–Kishino–Yano model (HKY; Hasegawa, Kishino & Yano, 1985), and the Generalised Time Reversible model (GTR; Tavaré, 1986) were predominantly applied to the molecular data.

All BI and ML analyses were run with a gamma distribution (Γ) without a proportion of invariant sites (*I*), where Γ or Γ + *I* was suggested, because combining Γ + *I* is known to cause convergence problems by creating two areas of equal probability in the tree landscape (Moyle et al., 2012). *I* was used when suggested as the sole analysis parameter.

#### Phylogenetic analyses

The partitioning scheme for the BI and ML analyses can be viewed in the Supplemental Information (Data Set S1). For the BI nuclear analyses two runs à four chains sampled the tree landscape at a temperature for the heated chain of 0.5 until the standard deviation of split frequencies was below 0.01. Trees were sampled every 1,000th generation. For the BI mitochondrial and combined molecular analyses were run two runs à four chains sampled the tree landscape at a temperature for the heated chain of 0.35 until the standard deviation of split frequencies was below 0.01. Trees were sampled every 5,000th generation. MrBayes v.3.2 (Ronquist et al., 2012) was used for all BI analyses. After completion, the statistics of all Bayesian analyses were checked in Tracer v.1.6 (tree.bio.ed.ac.uk) and convergence between runs was checked using the visualisation tool AWTY (Wilgenbusch, Warren & Swofford, 2004). A burnin of 0.25% was discarded.

The ML analyses for all molecular data sets were analysed with RAxML v.8.0.26 (Stamatakis, 2014) and included a rapid bootstrap search of 100 replicates starting at a random number seed.

### Combined molecular and morphology analyses

The TE matrix was compiled using the combined morphological and combined molecular data sets and consisted of 17,877 characters. The 87 taxa included two fossil and six extant non-cervid ruminant taxa and 29 fossil and 50 extant cervid taxa (Table 1; Fig. 3). The partitioning scheme can be viewed in the Supplemental Information (Data Set S1). For the BI analysis two runs à four chains sampled the tree landscape at a temperature for the heated chain of 0.35 until the standard deviation of split frequencies was below 0.01 using MrBayes v.3.2 (Ronquist et al., 2012). Trees were sampled every 5,000th generation. After completion, the statistics were checked in Tracer v.1.6 (tree.bio.ed.ac.uk) and convergence between runs was checked using the visualisation tool AWTY (Wilgenbusch, Warren & Swofford, 2004). The ML analysis for all molecular data sets were analysed with RAxML v.8.0.26 (Stamatakis, 2014) and included a rapid bootstrap search of 100 replicates starting at a random number seed. The MP analysis including a bootstrap analysis was undertaken using PAUP* v.4.0b (Swofford, 2002). The analysis used a heuristic search running 1,000 replicates. Sequences were added randomly using the TBR algorithm. The strict consensus tree was calculated from all trees sampled.

## Results

### Phylogenetic analyses of morphological data

#### Morphological data

**Figure 4:**
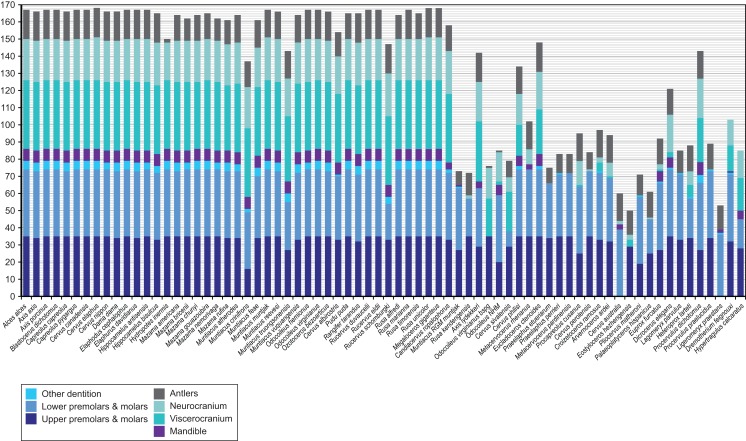
Overview of the characters available for each cervid species. Extant species are arranged in alphabetical order; fossil cervids and the two non-cervid fossils are arranged from the youngest to the oldest following the extant taxa. Morphological characters are subdivided into seven partitions indicated by the different colours of each bar. The *y*-axis represents the absolute number of present characters.

Figure 4 provides an overview of how well each species was sampled for morphological data. All fossil taxa are sampled for at least three of the seven partitions. The most incomplete fossil is *Eostyloceros hezhengensis* sampled from the literature with 70% missing data followed by *Ligeromeryx praestans* with 68% missing data. The most complete fossil cervids were *Megaloceros giganteus* with 0% missing data and *Candiacervus ropalophorus* with 6% missing data. Most of the other fossil taxa have around 50% missing data. All character scorings, character and character state descriptions are available on morphobank (http://morphobank.org/permalink/?P1021) and in the Supplemental Files (Data Sets S1–S3).

##### Cranium

All cervids share several anatomical features, such as two lacrimal foramina, a preorbital vacuity, and a lacrimal fossa (Fig. 1). In lateral view, the dorsal outline is convex at the braincase, concave at the fronto-nasal transition and straight at the nasals. The anterior extension of the snout is moderate depending on the overall size of the cervid species. The basicranial outline in lateral view is flexed. The preorbital vacuity varies in size and form, the lacrimal fossa can be deep and round, covering a large proportion of the facial aspect of the skull, shallow, or barely visible (particularly in females). The position of the two lacrimal foramina on the orbita rim (more internally or externally) and the position to each other is variable. A detailed description of the craniodental morphology for each cervid species investigated is in Heckeberg (2017a).

**Figure 5:**
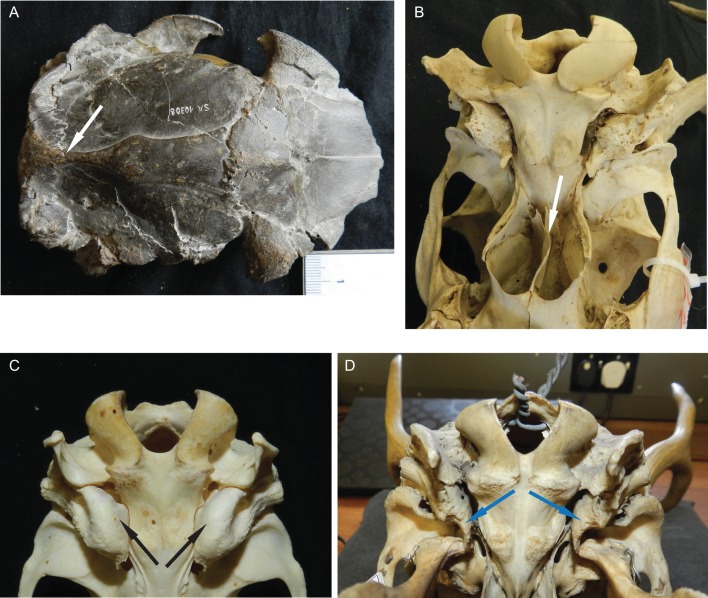
Details of the cervid cranial anatomy. (A) Cranium of *Dicrocerus elegans* (MNHN Sa 10308) in dorsal view. The arrow indicates the sagittal crest. (B) Basicranium of *Odocoileus hemionus* (MNHN AE724). The arrow indicates the vomerine septum typical for Capreolinae. (C) Basicranium of *Axis axis* (ZSM 1958-88). The arrows indicate the large inflated auditory bullae, rarely observed in cervids. (D) Basicranium of *Ozotoceros bezoarticus* (UMZC H.18781). The arrows indicate the small flattened auditory bullae with prominent processes.

Some Miocene cervids have a sagittal crest (e.g. *Dicrocerus* and *Procervulus*), which is absent in all other cervids (Fig. 5). The number and size of supraorbital foramina and presence and absence of the supraorbital sulcus are variable and could potentially be features to distinguish groups of cervids; however, more specimens per species need to be investigated to confirm this. The presence of an extended vomerine septum and the division between the temporal foramina is characteristic for Capreolinae (Fig. 5). Most cervids have small, oval auditory bullae, some species have large inflated bullae (e.g. *Axis*) (Fig. 5).

**Figure 6:**
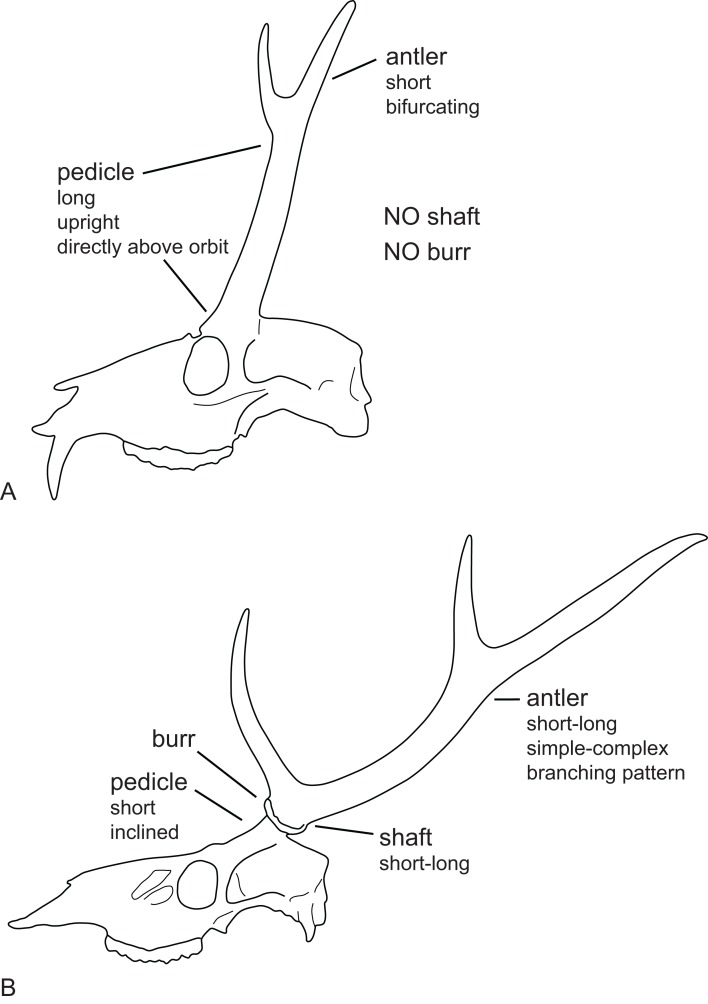
Cervid antler evolution. (A) Cranium of a typical Miocene cervid showing the characteristics of early pedicles and antlers. (B) Cranium of an extant cervids showing features of the pedicles and antlers seen in modern cervids (Drawing by Nicola Heckeberg).

Most Miocene cervids have long pedicles, the insertion point of the pedicle is directly above the orbit and the pedicle is upright (Fig. 6). Muntiacini, *Euprox* and *Eostyloceros* have long strongly inclined pedicles. In most other cervids the pedicles originate more posteriorly to the orbit, are inclined at 45–60, and short. *Mazama* and *Pudu* have strongly inclined and short pedicles.

##### Antlers

Based on evidence from comparative anatomy (Heckeberg, 2017b), the cranial appendages of early Miocene cervids, including lagomerycines, were shed and followed the same principles of the antler cycle as extant cervids and are therefore to be considered as antlers. Even though antlers are species-specific, they have a high variability, intraspecifically and ontogenetically. No antler looks exactly the same, not even the left and the right antler of the same individual are identical. Also, antlers change from one year to the next; in addition pathologies, abnormal growth, and other phenomena occur.

While cervid genera and most species can be qualitatively distinguished based on antler morphology, translation of these distinctions into discrete characters for quantitative or phylogenetic analyses is difficult. Convergence, which can be distinguished by eye, but is sometimes too subtle to be scored differently in the character matrix is the reason for this. Three morphotypes can be distinguished in cervids.

#### Morphotype 1

This morphotype includes all cervids with single-tined or bifurcating antlers; *Mazama* and *Pudu* have single-tined antlers (*Pudu* antlers rarely develop a bifurcation). *Elaphodus cephalophus* has minute, single-tined antlers. All *Muntiacus* species have bifurcating antlers on elongated inclined pedicles. *Hippocamelus* has a bifurcating antler morphology with an open angle between the brow tine and main tine; the main tine can have additional small tines. Fossil cervids with a bifurcating antler morphology include *Procervulus*, *Dicrocerus*, *Heteroprox*, *Euprox* and presumably *Cervus australis*.

#### Morphotype 2

This morphotype includes all cervids with antlers showing exactly three tines, e.g. *Rusa*, *Axis*, *Capreolus* and *Ozotoceros*. The three tines are organised either in a way, where the brow tine forms a more acute angle to the main beam with the tip of the brow tine pointing posteriad (*Axis* and *Rusa*), or where it forms an open angle with the tip of the brow tine pointing more upwards or forwards (*Capreolus* and *Ozotoceros*).

Fossil cervids of the “Morphotype 2” include *Axis lydekkeri*, *Rusa kendengensis*, *Metacervocerus pardinensis*, *‘Cervus’ philisi*, and *Metacervocerus rhenanus* with the brow tines pointing posteriad, *Procapreolus cusanus* with the brow tines pointing upwards. *Pliocervus matheronis* antler remains are too fragmentary to infer the direction of the brow tine unambiguously. It was also suggested that this species had presumably four tines (Croitor, 2014); however, as this could not be observed on the studied specimens and literature, it was scored as possessing three tines.

#### Morphotype 3

This morphotype contains the more complex or palmated antlers and is present in *Alces*, *Blastocerus*, *Cervus*, *Dama*, *Elaphurus*, *Odocoileus*, *Rangifer* and *Rucervus*. *Blastocerus dichotomus*, *Cervus albirostris* and *Cervus nippon* have an antler bauplan, which produces not more than four tines in adults (accessory smaller tines not included). In *Elaphurus* it is difficult to distinguish between main tines and accessory tines. Characteristic for *Cervus elaphus* are paired lower tines, called brow tine and bez tine and trez tine (Lister et al., 2010). *Dama dama* and *Rangifer tarandus* have a ramified palmated morphology, while *Alces alces* has a palmated morphology without ramification, and thus form a subgroup within “Morphotype 3”. The remaining eight extant cervid species develop more complex antlers with an increasing number of tines from year to year, which is erroneously widely assumed to happen in all cervids.

Fossil cervids of the “Morphotype 3” include *Croizetoceros ramosus*, *Eucladoceros ctenoides*, *Lagomeryx parvulus*, *Ligeromeryx praestans*, *Arvernoceros ardei*, *Praeelaphus perrieri*, *Megaloceros giganteus* and *Palaeoplatyceros hispanicus*. Lagomerycines possess coronate antlers without a shaft, while *Palaeoplatyceros* has palmated antlers without any other tines, and *Croizetoceros ramosus* shows a serial organisation of small tines on the main beam. *Praeelaphus perrieri* has a distally trifurcating main beam with a basal brow tine, which is similar to the condition in *Arvernoceros ardei*, where the branching part of the main beam sometimes forms a palmation. The antler morphology of *Eucladoceros ctenoides* resembles that of *Cervus elaphus* with several short proximal tines, similar to the bez and trez tine. *Megaloceros giganteus* has enormous ramified palmated antlers similar to those of *Dama*. Also characteristic for Megacerini are flattened basal brow tines similar to *Rangifer* (Lister et al., 2010).

##### Dentition

Some dental characters are highly variable and thus difficult to score unambiguously. Despite convergent modifications depending on dietary requirements, a species-specific pattern underlies these adaptations in most species (N. Heckeberg, 2017, personal observation), particularly in the lower premolars and upper molars. The difficulty is to score these species-specific patterns without scoring the convergent adaptations and the intraspecific variability.

##### Upper premolars and molars

**Figure 7:**
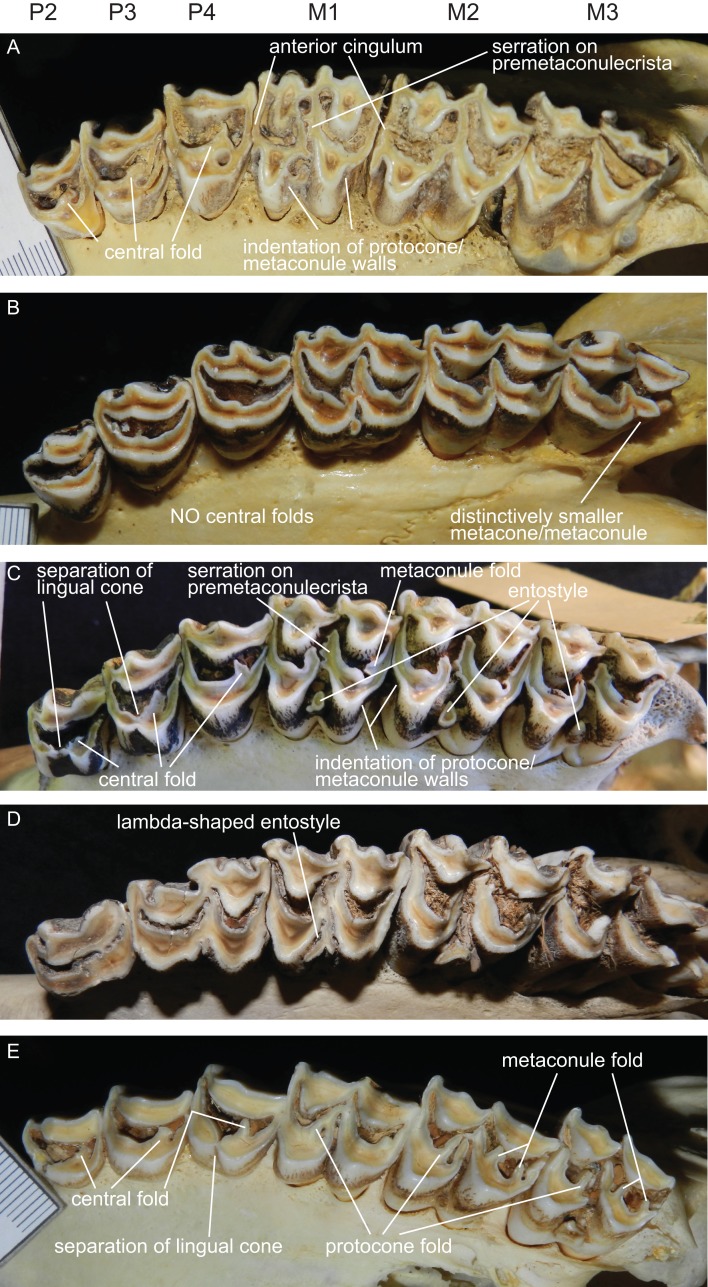
Details of the upper dentition. Close ups of the upper dentition of selected cervids showing the most striking features. (A) *Rucervus duvaucelii* (ZSM 1957-60), (B) *Rangifer tarandus* (ZSM 1959-211), (C) *Rucervus eldii* (UMZC H16194), (D) *Elaphurus davidianus* (UMZC H16235) and (E) *Odocoileus hemionus* (ZSM 1971-720).

The upper incisors and the P1 are absent in cervids. The upper premolar row is characterised by robust, compact, predominantly horseshoe-shaped teeth. P3 and P4 are less variable, P2 can have more rectangular or triangular outlines, particularly in early fossil cervids. All premolars have at least one prominent central fold, except for *Rangifer*, in which central folds are missing (Fig. 7). Sometimes there are tiny additional folds, or the main central fold is serrated. A separation of the lingual cone into an antero- and posterolingual cone is relatively common (Fig. 7). In all Miocene cervids the P2 is longer than the P4, while in extant taxa the P4 is most often longer than the P2. Several fossil cervids have a well developed lingual cingulum (Fig. 7).

The upper molars are all two-lobed and quadrangular with only little variation in morphology. The posterior lobe of the M3 is distinctively smaller than the anterior one in most species. The entostyles are variably present. In some species the entostyle(s) has/have a λ-shaped morphology, especially in later wear (*Axis*, *Rusa*, *Rucervus* and *Elaphurus*) (Fig. 7). Metaconule folds are variably present within Cervinae and Capreolinae and are mostly small. Protocone folds are usually absent in Cervinae, while they are regularly present Capreolinae, often well developed on all molars (Fig. 7). The same applies to fossil cervids, where tiny metaconule folds are much more common than protocone folds. Only in Miocene cervids protocone folds are common. However, in these species it often looks more like a bifurcation of the postprotocrista than a fold originating from the crista, particularly when the internal part of this bifurcation is longer than the external as on M2 in *Dicrocerus*. It is not entirely evident, whether these are two independent structures or the same structure with variable characteristics. Several species have an anterior cingulum and some fossil cervids have a lingual cingulum. The protocone and metaconule folds are variably present. In a few species, for example *Rucervus eldii*, the premetaconulecrista is serrated (Fig. 7) More details are in Heckeberg (2017a).

##### Lower premolars and molars

**Figure 8:**
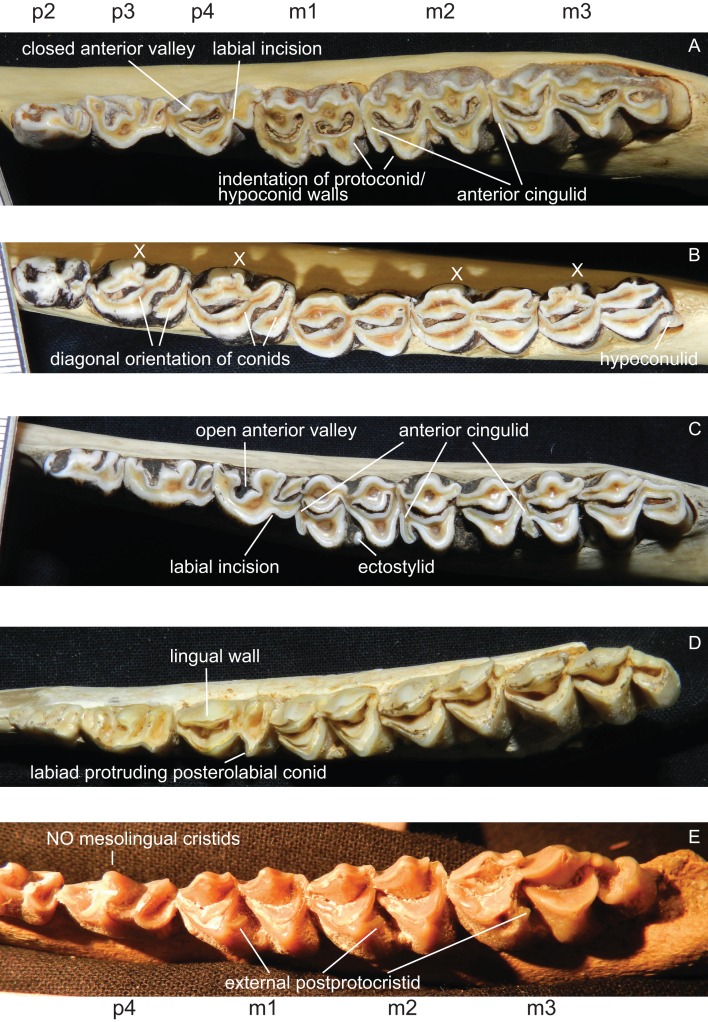
Details of the lower dentition. Close ups of the lower dentition of selected cervids showing the most striking features. (A) *Rucervus duvaucelii* (ZSM 1957-60), (B) *Rangifer tarandus* (ZSM 1959-211), (C) *Rucervus eldii* (UMZC H16194), (D) *‘Cervus’ philisi* (NMB St.V. 605) and (E) *Procervulus* (MNHN LRM 114).

**Figure 9:**
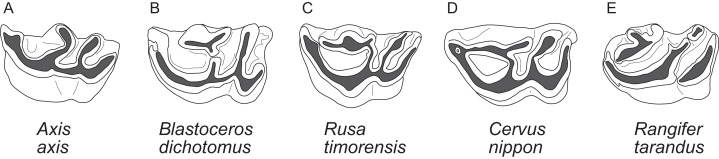
Variability of p4 in cervids. This sequence of the lower left p4 shows different degrees of modification of tooth elements, starting with an open anterior valley (A), development of mesolingual cristids (B), connexion of mesolingual cristids to other tooth elements (C), closing of the anterior valley (D) and re-arrangement of tooth elements with a diagonal orientation (E). The premolars with modifications shown in (D) and (E) resemble the molars (Drawing by Nicola Heckeberg).

p1 is usually absent in cervids, it was present in individual *Lagomeryx parvulus* specimens. The p2 has a simpler morphology with fewer elements compared to p3 and p4. A strong reduction in p2 length could be observed in *Mazama* and particularly in *Ozotoceros*. In a few specimens the p2 is missing. Mesolingual cristids were variably present in p3 and p4 (absent in *Axis*, often absent in early Miocene species) (Fig. 8). p3 and p4 often show modifications of tooth elements, which make them more similar to molars to a different extent. While the p3 shows these modifications only in a few species and not to the same extent as p4, the p4 is modified in many species, especially in *Rangifer* and *Alces* (Fig. 9). The labial incision on premolars is rarely and weakly developed in p2; it is more often developed on p3, and most often occurs on p4 (Fig. 8). p4 is the most variable tooth in cervids.

Some species show a spike like extension of the posterolabial conid of the p4 towards labiad; these species are *Capreolus capreolus*, *Capreolus pygargus*, *Blastocerus dichotomus*, *Hippocamelus* spp., *Hydropotes inermis*, *Ozotoceros bezoarticus*, *Croizetoceros ramosus*, *Procapreolus cusanus* and *‘Cervus’ philisi* (Fig. 8). Whether this feature can be used as a phylogenetic character and whether it is indicative of affiliation to a certain subclade has to be investigated in the future.

All lower molars have a similar morphology; m1 and m2 are two-lobed, m3 is three-lobed. The orientation of the lingual conids and cristids may be more diagonal in some species. Ectostylids are variably present on one to three molars. never high, nevertheless they become involved in wear in aged individuals (Fig. 8). In most Miocene cervids and in *Cervus australis* external postprotocristids are present on all molars (Fig. 8). Anterior cingulids are present in several species, usually more prominent on the anterior molar position(s). In *Rucervus* and *Rusa* the anterior cingulids are particularly prominent (Fig. 8). In *Rucervus* and also to a lesser extent in *Rusa* and *Axis* the anterior and posterior labial walls of the lobes of the lower molars are indented (Fig. 8). The metastylids can be bent labiad in some species, for example *Alces*. The third lobe on m3 is variable; most often the hypoconulid and entoconulid are connected via the postento- and posthypoconulidcristids and form a crescent-shaped structure. Sometimes the third lobe is reduced to one of these elements or has an additional fold on the posthypoconulidcristid. In a few individuals the third lobe is missing entirely. More details are in Heckeberg (2017a).

##### Lower incisors and canines, upper canines

All Miocene cervids have enlarged upper canines, which are curved posteriad. From the Pliocene onwards, the upper canines become reduced in size and are lost in some species. Extant muntiacines have enlarged upper canines, similar to those of Miocene cervids. *Hydropotes* has strongly elongated sabretooth-like upper canines, which differ in morphology from those in muntiacines and early fossil cervids. In all other extant species upper canines are reduced in size or missing entirely. Most cervines possess small upper canines. Adult capreolines rarely have upper canines, while some capreoline juveniles have deciduous upper canines.

The lower incisors, i1–i3, have a simple spatulate morphology. The crown width decreases from i1 to i3, that is i1 typically is distinctively broader than i2 and i3. Exceptions are *Alces*, *Hippocamelus*, and *Pudu*, where i1 is only a little broader than i2. All lower canines in Cervidae are incisiviform. More details are in Heckeberg (2017a).

#### Standard phylogenetic analyses

**Figure 10:**
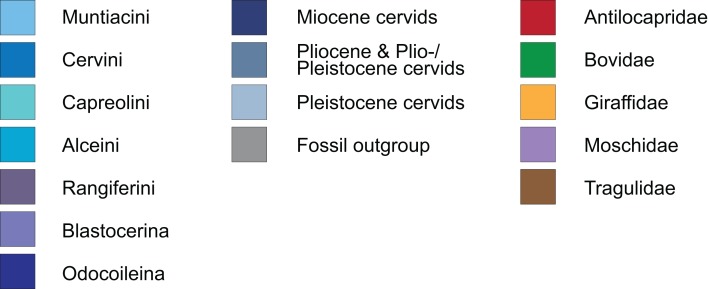
Colour code. The colour code provides the key to taxonomic groups studied here and applies to all topologies within the present work.

The results of the ss analyses (Supplemental File S2) showed that the data set is best analysed unpartitioned, using the Γ distribution and using character state ordering. However, BI and MP analyses were run unordered and ordered for each character set for comparison. See Table 1 for details. Figure 10 provides a key to the colour coding of the taxonomic groups.

**Figure 11:**
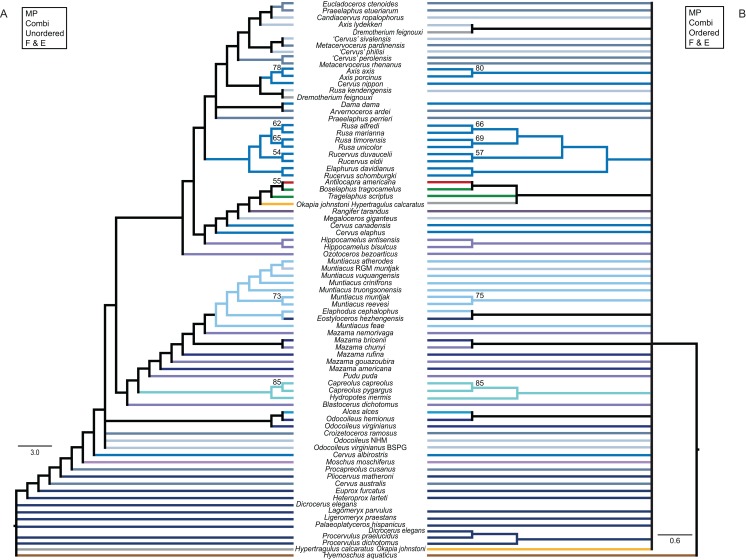
Topologies from the morphological analyses. The topologies of the maximum parsimony analyses of the combined morphological data set are shown. (A) Topology based on the unordered data set, (B) topology based on the analysis using character state ordering. Node support values are given as bootstrap support values.

The unordered and ordered MP topologies support monophyletic Capreolini, a sister taxon relationship of *Axis axis* and *Axis porcinus* and *Muntiacus muntjak* and *Muntiacus reevesi*, and an *Elaphurus*-clade (Fig. 11). The *Elaphurus* was always recovered in the analyses based on the dental and combined data set, in most topologies fully resolved. It consists of the *Rusa*-clade, which often has *Rusa alfredi* as the sister taxon to the other three *Rusa*-species, of *Rucervus duvaucelii* and *Rucervus eldii* as the sister taxa to each other and to the *Rusa*-clade, and *Elaphurus davidianus* and *Rucervus schomburgki* as the sister taxa to each other and to the latter taxa. Cervini were never monophyletic in the analyses here based on the morphological data sets. The sister taxon relationships of *Rusa alfredi* and *Rusa marianna* and *Rusa timorensis* and *Rusa unicolor* are the only consistently recovered cervine clades in all topologies based on the cranial matrix. The higher hierarchical clades could not be recovered. The positions of *Dremotherium feignouxi*, *Okapia johnstoni*, *Hypertragulus calcaratus* and *Dicrocerus elegans* differed in both topologies.

#### Single fossil analyses

**Figure 12:**
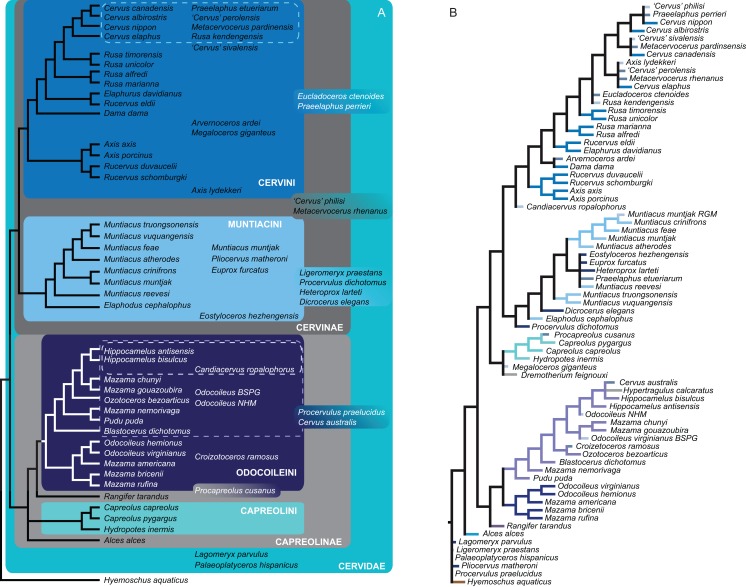
Topologies from the SFA and FPA approaches. (A) The topology summarises the systematic position of the fossils based on the SFA (see Supplemental File S3 for individual topological placements). The gradually shaded boxes indicate different observed positions, e.g. *Procapreolus cusanus* was placed within Capreolinae in one analysis and within Odocoileini in a different analysis. (B) The topology is the result of the FPA analysis.

The results from the three sets of 31 analyses including each only one fossil at a time are in the Supplemental File S3 and summarised in Fig. 12.

#### Fossil placement algorithm

The FPA analysis resulted in a resolved topology (Fig. 12). Cervinae, Cervini, Muntiacini, Capreolini and Odocoileini were monophyletic. Many positions of fossil cervids were as expected from qualitative observations, for example those included in Cervini, whereas some were unexpected, for example the sister taxon position of Capreolini–Cervinae and placements of some fossil cervids, for example *Praeelaphus etueriarum*, *Megaloceros giganteus*, *Cervus australis*. Some Miocene cervids were included in Muntiacini, some were placed between the outgroup and all other cervids.

### Phylogenetic analyses of molecular data

#### Molecular data

##### Nuclear genes

**Figure 13:**
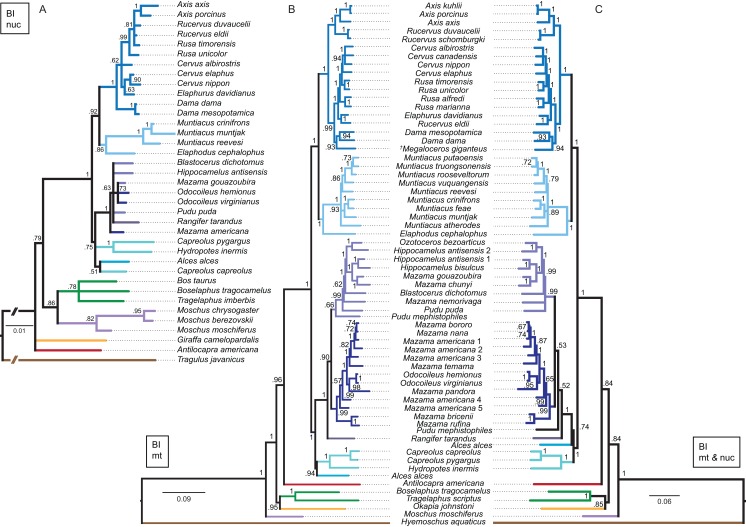
Topologies resulting from the molecular data sets. The topologies of the Bayesian inference analyses of the combined nuclear data set (A), the combined mitochondrial data set (B) and the combined molecular data set (C) are shown. Nuclear markers were available for fewer taxa than mitochondrial markers. The mitochondrial and molecular combined topologies are congruent except for the position of *Alces alces* and *Pudu mephistophiles*. Node support values are given as Bayesian posterior probabilities.

Although interpretations of the systematic relationships on genus and species level was difficult in the single gene topologies due to low taxon sampling and/or lack of resolution, the combined nuclear topology was well resolved and supports the higher hierarchical clades. The BI and the ML topologies were largely congruent (Fig. 13). There was no split into Odocoileina and Blastocerina as observed in the topologies based on the mitochondrial markers. The unexpected placement of *Capreolus capreolus* in this topology may be caused by the possibly contaminated *Sry* sequence of this species.

##### Combined mitochondrial genes

The BI topology of the combined mitochondrial analysis showed higher support values for the majority of nodes than the *Cytb* only topology, but lower support values for some nodes than for the mtG analysis. The ML topology differed in generally lower support values for most nodes, but was otherwise largely congruent (Fig. 13). The placement of non-cervid ruminants differed in both topologies. The main difference concerning cervid taxa is the position of *Pudu mephistophiles* (based on the correct *Cytb* sequence (Heckeberg et al., 2016)), which was the sister taxon to Blastocerina in the BI topology and the sister taxon to *Rangifer* and Odocoileini in the ML topology. This combined topology includes the polyphylies for *Rucervus*, *Hippocamelus*, *Odocoileus*, *Mazama* and *Pudu*.

##### Combined molecular analyses

The BI and ML topologies of the combined nuclear and mitochondrial analyses were largely congruent, the support values were partly lower, particularly in the ML topology, in comparison to the topologies based on the mitochondrial markers (Fig. 13). Both topologies differed in the position of non-cervid ruminants, and the positions of *Alces alces* and *Pudu mephistophiles*, which remain uncertain. The split of Odocoileini into Blastocerina and Odocoileina was supported.

#### Combined molecular and morphological analyses

##### Bayesian inference

**Figure 14:**
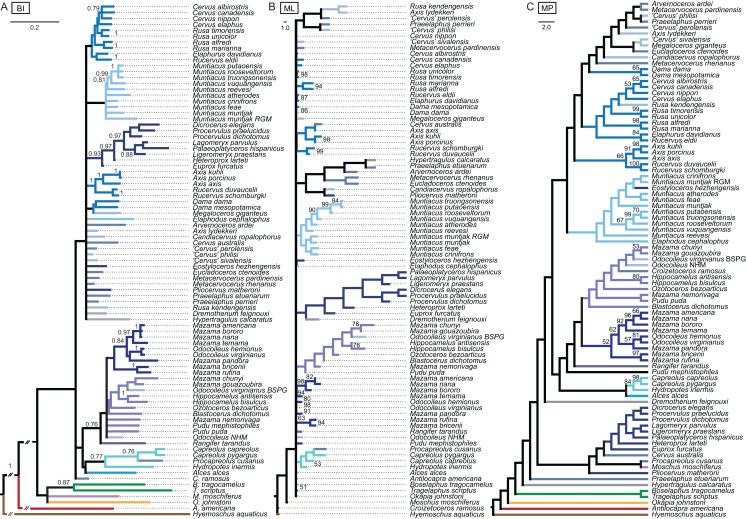
Topologies from the combined molecular and morphological analyses. The topologies of the combined molecular and morphological analyses using Bayesian inference (A), maximum likelihood (B) and maximum parsimony (C) are shown. Node support values are Bayesian posterior probabilities (BI) and bootstrap support values (ML and MP).

The BI combined topology was largely unresolved (Fig. 14). Most extant cervids formed clades; the three *Axis* species and two *Rucervus* species formed a well supported clade. There was also an supported clade including eight Miocene cervids.

##### Maximum likelihood

In the ML combined topology the nodes were poorly or not at all supported (Fig. 14). Some extant clades were recovered, for example Muntiacini, Odocoileina, Capreolini. Eight Miocene cervids formed a clade.

##### Maximum parsimony

The nodes in the MP combined topology are largely unsupported (Fig. 14). *Procapreolus cusanus* was unexpectedly placed as the sister taxon to *Moschus*. Cervinae, Cervini, Muntiacini, and Odocoileini form unsupported clades. Capreolini is a supported clade. All Miocene cervids except for *Eostyloceros hezhengensis* form a clade.

**Figure 15:**
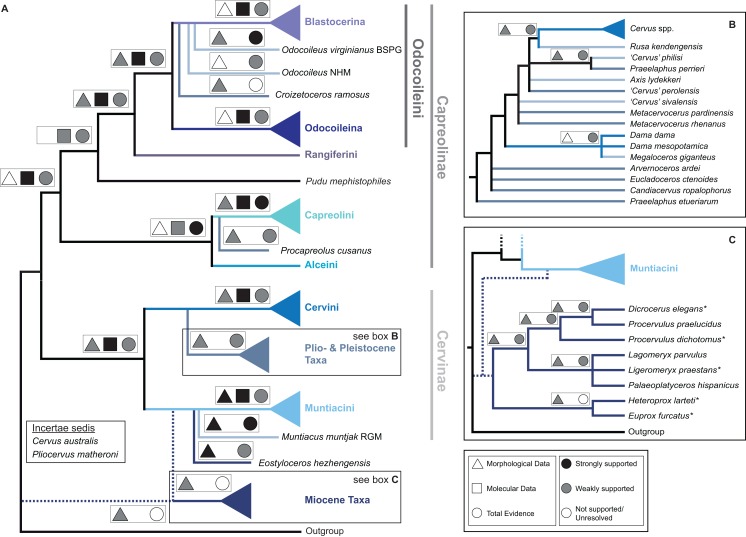
The qualitative summary topology of all analyses is shown. (A) Represents the overview of the systematic relationships of higher cervid taxa including the positions of some fossil cervids, (B) shows the systematic relationship of several Plio- and Pleistocene cervids and (C) shows the systematic relationships of Miocene cervids.

Figure 15 qualitatively summarises the topologies from all analyses undertaken. The topology was not generated by an analysis but was drawn to show the consensus of all topologies and which character sets support the respective nodes.

Supplemental Information on the phylogenetic analyses and topologies can be found in the Suppemental Files (S2; Data Sets S1 and S3).

## Discussion

For the first time, fossil and extant cervids were combined in the so far most extensive data set including molecular and morphological data. Various data sets and partitions were analysed under different optimality criteria. In addition, the alternative approaches SFA and FPA were undertaken to investigate the systematic positions of fossils. The results provided new insight into the systematic relationships of fossil cervids and extant cervids. Many hypotheses about the systematic relationships of extant taxa could be confirmed; however, known controversies persisted, but could be specified in more detail. For most fossil cervids, affiliations to extant relatives were found, which have not been quantitatively tested previously.

Combining different data types helped to investigate the systematic relationships in detail and to reconstruct the evolutionary history of cervids. The initial separate analyses of the different data sets provided insights into the phylogenetic signal of the respective data. Some areas of the morphological topologies were congruent with the molecular topologies, some were not. However, the support of the morphological topologies did not contradict the molecular hypotheses.

Genotypic data partitions usually contain proportionally more characters than osteological data, which is assumed to be crucial for accuracy. On the other hand, osteological data partitions can be sampled for many more taxa, which partly cannot be sampled for molecular data, that is fossils (O’Leary, 1999). Thus, morphological characters still have relevance in times of genomic analyses and serve as an independent test for molecular data, because of the relative distance between phenotype and genotype and different evolutionary dynamics of both types of data. Because selection targets on the phenotype, the resulting topology could potentially provide information on the selective history of taxa (Lee & Camens, 2009; Groves, 2014). If the same topology is supported by different data sources and reflects biological evidence at all scales (principle of consilience), it is more likely that the topology is ‘correct’ (Bibi, Vrba & Fack, 2012).

The challenges of the data sets here were the high levels of homoplasy, particularly in the morphology, and the rapid radiations of some lineages, for example Odocoileini. Consensus might be difficult to achieve, because short branch lengths and/or lack of resolution potentially represent a genuine rapid diversification of clades, which may not be further solved just by increasing the sequence length or the taxon sampling. Markers that are less influenced by convergent evolution, such as rare genomic changes or cytogenomics may be useful additions in the future (Rokas & Holland, 2000; Price, Bininda-Emonds & Gittleman, 2005; Hernández Fernández & Vrba, 2005).

### Data partitioning

Partitions of morphological data have different capabilities to fossilise, for example teeth are more resistant to diagenetic processes than bones; this may influence the phylogenetic signal (Sansom & Wills, 2017). The phylogenetic signal of dental and cranial traits were tested separately before combining both data sets to account for this phenomenon. Even though the morphological characters can be assigned to seven partitions according to different anatomical regions of the cranium and dentition (Fig. 4), the combined data set was run unpartitioned based on the outcomes of the ss ananlyses (see Supplemental Information 2). The phylogenetic signal of the cranial data sets differed from that of the dental character set, but did not result in conflicting hypotheses (Fig. 11; Supplemental Information 3), which is contrary to the observations made by Sansom, Wills & Williams (2017). Some splits from the dental or morphological combined topologies were supported by the molecular topologies, and no conflicting hypotheses were found. The combined morphological topologies are likely to be dominated by the phylogenetic signal from the dental data set. Pattinson et al. (2015) showed that combining data sets nevertheless leads to a better performance.

### Models of evolution

Since it is not fully understood how the standard models of molecular evolution (e.g. HKY, GTR) translate variable rate frequencies and substitution rates to morphological data (Spencer & Wilberg, 2013), the only model of morphological evolution, which is widely used in model-based phylogenetic algorithms (BI and ML), is the Markov k (Mk) model by Lewis (2001).

Morphological character states do not mean the same across different characters, that is state ‘1’ in character X is differnet from state ‘1’ in character Y. Therefore, it is challenging to allow for asysmmterical rates of character change (Wright, Lloyd & Hillis, 2016). So far, there is no appropriate evolutionary model for morphological characters in model-based approaches such as BI and ML to account for this (O’Reilly et al., 2016).

Spencer & Wilberg (2013) noted that, although topologies from model-based approaches, particularly ML, are typically better resolved than strict consensus topologies from parsimony analysis, the better resolution is not necessarily meaningful. The apparent better resolution may simply be a result of an incorrect model of morphological evolution.

However, in BI analyses it is possible to allow for asymmetrical character state changes by using priors on the equilibrium state frequencies of characters and specifying a distribution hyperprior (Wright, Lloyd & Hillis, 2016). Several recent studies showed that the Mk model outperforms parsimony and that it is the most accurate model for phylogenetic reconstructions on multistate morphological character sets (Wright & Hillis, 2014; Wright, Lloyd & Hillis, 2016; Puttick et al., 2017).

### Comparison of mitochondrial vs. nuclear vs. TE topologies

Previous studies demonstrated that combining mitochondrial and nuclear markers increases robustness of higher hierarchical cervid clades (Randi et al., 1998). The topologies resulting from nuclear markers often agree with morphology, but often contradict topologies resulting from mitochondrial markers (Bibi, 2014). There are few phylogenetic reconstructions for cervids based on nuclear markers (Cronin et al., 1996; Gilbert, Ropiquet & Hassanin, 2006). Analyses of nuclear markers have the potential to characterise the distribution of genetic variation (Balakrishnan et al., 2003). Combining and interpreting nuclear and mitochondrial markers can help to uncover recent hybridisation events, as in *Elaphurus davidianus*, which takes up different positions when analysed with mitochondrial markers compared to nuclear markers (Fig. 13).

Incorporating more nuclear DNA is crucial to test relationships in ruminant systematics based on mitochondrial DNA and should be sequenced for a broader range of taxa than is available to date.

### Aspects of the evolution of Cervidae

#### Morphological evolution

##### Cranium

The cranial morphology of cervids is highly conservative (Lister, 1996; Merino & Rossi, 2010). Also, some morphological characters in ruminants likely are the results of convergent evolution and thus are homoplastic, which may cause difficulties in reconstructing phylogenetic relationships (Bouvrain, Geraads & Jehenne, 1989; Douzery & Randi, 1997). Despite the homoplasy, some clades were well defined and re-occurring across different data sets in the topologies here.

Differences in the size of the praeorbital vacuity are primarily species specific, but have also an ontogenetic component, since they are often smaller in aged individuals. Similarly, the lacrimal fossa varies in size and depth in different species, presumably depending on the presence, size, and usage of the lacrimal gland and sexual dimorphism. The position of the lacrimal foramina to each other and on the orbita rim can potentially be used to distinguish groups of cervids. The consistent presence of two lacrimal foramina is typical for cervids, but is also present in some bovid species. In *Dremotherium feignouxi* sometimes only one lacrimal foramen is present (Costeur, 2011). The contact of the lacrimal and the frontal at the orbita rim without interlocking sutures was first observed in Rössner (1995). This trait is most likely an intraspecific variability and could be an effect of ageing.

Evolutionary trends observed in Pliocene cervids include an increase of the overall body size, a decrease of the pedicle length relative to the antler length and an associated increase of the antler length (Heintz, 1970). The degree of inclination of the pedicles changes through time and is presumably a result adapting to rich vegetation. With the stronger inclination the insertion point of the pedicle on the skull moved posteriad. The pedicle in early Miocene cervids is entirely above the supraorbital process and not in contact with the braincase; the pedicles are vertical in lateral view, parallel or converging in frontal view. The shortening of the pedicles could be related to the increasing size of antlers, because a longer and heavier set of antlers would put a biomechanically unfavourable leverage on long pedicles.

Basicranial and ear region characters were not yet widely used when inferring morphological phylogenies, but were assumed to have strong potential to provide characters, which are less prone to convergent evolution caused by climatic change (Janis & Theodor, 2014). Recently, it has been shown that traits of the inner ear provide useful characters with phylogenetic signal (Mennecart et al., 2016, 2017).

##### Antlers

There is broad consensus that antlers originated only once (Loomis, 1928; Azanza & Morales, 1989; Azanza, 1993a, 1993b; Azanza, DeMiguel & Andrés, 2011; Heckeberg, 2017b). The antlers of most Miocene cervids have a simple bifurcating pattern, sometimes with an additional tine, or are coronate (Azanza, DeMiguel & Andrés, 2011). These antlers are relatively short, do not have a shaft and the bifurcation originates directly from a broad antler base. From the late Miocene onwards, more complex branching patterns developed, the length of antlers increased and antlers developed a shaft below the first bifurcation. Evolution of size and complexity of antlers is associated with reduction or loss of upper canines (Scott, 1937; Beninde, 1937; Geist, 1966; Brokx, 1972).

In extant cervids, short and simple antlers and long and more complex or palmated antlers are present. Many extant cervids develop exactly three tines (Heckeberg, 2017b). The three antler morphotypes have previously been associated with ecological habitats: simple antlers for the tropics, a three-tined antler plan for woodland areas typical in East Eurasia or India, and the large and complex display organs in temperate regions (Pitra et al., 2004). The simple antlers in *Mazama* and *Pudu* are considered as a secondary adaptation to dense vegetation.

There is a lot of inter- and intraspecific variation in antlers (Goss, 1983; Heckeberg, 2017b). The high variability of antlers is a problem particularly in fossil taxa, where the entire intraspecific variation cannot always be observed due to the lack of a sufficient number of specimens or the incompleteness of ontogenetic stages. The taxonomy of fossil cervids is often based on antler morphology, because antlers are easy to identify and numerous in the fossil record and antler morphology is more distinctive than other anatomical characters (Kurtén, 1968; Fry & Gustafson, 1974; Lister et al., 2010; Merino & Rossi, 2010). Thus, the validity of some fossil cervid taxa is doubtful. To base classifications just on antler morphology is problematic for the given reasons.

In contrast to Loomis (1928), Gentry, Rössner & Heizmann (1999) stated that cranial appendage morphology proved to be more suitable than tooth morphology to distinguish species of horned Pecora. It is true that different cervid species can be easily identified based on their antler morphology (branching pattern, orientation, size). Antler characters were often used to solve intra-subfamily relationships, but they are problematic because of convergent development and subsequent homoplasy in antler characters (Pitra et al., 2004).

Since Cervidae is diagnosed by the presence of antlers (Janis & Scott, 1987; Pitra et al., 2004), the reason for the absence of antlers in *Hydropotes inermis* species was controversially discussed; a primitive condition and secondary loss have been suggested (Bouvrain, Geraads & Jehenne, 1989; Hernández Fernández & Vrba, 2005; Hassanin et al., 2012; Schilling & Rössner, 2017). The robust placement of *Hydropotes inermis* as the sister taxon to *Capreolus* proves the secondary-loss hypothesis. However, the process of antler loss is not known, neither is the process(es), which trigger(s) the growth of the first set of antlers in antler-bearing species. *Hydropotes inermis* might be the key to investigate these processes.

##### Dentition

Variations of accessory dental elements in combination with the degree of modifications of tooth elements of premolars can be used to identify genera or species. Widely accepted evolutionary trends in cervids concerning the dentition are increasing hypsodonty, the reduction of the premolar row length and the reduction or loss of upper canines (Heintz, 1970; Dong, Pan & Liu, 2004). However, the hypsodonty index, although widely used in ruminant phylogeny, has been considered to be a misleading character due to its ambiguous definition and convergent evolution among all large herbivorous mammals (Janis & Scott, 1987; Hassanin & Douzery, 2003).

The first deer had brachyodont dentition and were considered as leaf-eaters; recent dental analyses generally support these findings, but also showed that *Procervulus ginsburgi* likely was a seasonal mixed feeder. Based on this a facultative leaf-grass mixed feeding strategy with preference for leaf-eating is likely the primitive dietary state in cervids and ruminants (DeMiguel et al., 2008).

Ginsburg & Heintz (1966) regarded the bifurcation of the postprotocrista into an internal and external crista as a derived cervid character based on its presence in *Dicrocerus* and *Euprox*. *Amphimoschus* is the only other non-cervid pecoran species that shows this trait (Janis & Scott, 1987). The bifurcated postprotocrista was regarded as an advanced cervoid character in Janis & Scott (1987), while later this character is referred to as ‘primitive presence of bifurcated protocone’. In extant cervids, this feature is present in *Odocoileus*, *Blastocerus*, *Alces*, *Mazama*, *Pudu* and *Capreolus* (Janis & Scott, 1987). These observations could be confirmed here by morphological comparisons. One specimen of *Palaeoplatyceros hispanicus* (MNCN 39181) shows both a bifurcating postprotocrista and a tiny protocone fold on the preprotocrista. This indicates that both structures may in fact be developmentally independent, however, as this could only be observed in one specimen, it remains speculation.

Throughout the evolutionary history of cervids the lingual cingulum, regularly present on molars and sometimes even on premolars of fossil cervids, becomes reduced and eventually lost in extant cervids. In *Rucervus*, *Rusa*, and *Axis* the anterior and posterior lingual walls of the molars tend to be indented; this is also observed in *Axis lydekkeri*, *Rusa kendengensis* and *‘Cervus’ sivalensis*.

The lower p2 is the tooth with the fewest changes in occlusal morphology throughout cervid evolution; only a shortening is observed in most extant taxa and in a few individuals p2 was lost. The lower p3 and p4 are more variable (Fig. 9) and sometimes become more similar to molars in modern cervids.

The elongated upper canines in *Hydropotes inermis* are used in intraspecific fights. It is likely that the presence and/or size of upper canines is somehow genetically linked with the antlers, which leads to the question, why female deer have upper canines, too (Brokx, 1972). Even though they are often much smaller, especially in species, where males have enlarged upper canines, they are present without any obvious function. In other ungulates, where males use their canines in intraspecific fights, for example in equids, upper and lower canines are lost in almost all females. Much more research is needed to find this link and associated interactions and effects on behaviour.

#### Evolutionary history

During the Eocene, selenodont artiodactyls diversified and ruminants were the only successful descendants from this radiation. Subsequent rapid radiations of ruminants resulted in the most diverse group of large mammals today (Hernández Fernández & Vrba, 2005).

Collision of the African and Indian continents with Eurasia around 40 mya caused drastic environmental changes triggering artiodactyl evolution. The expansion and diversification of grasslands at the Eocene-Oligocene-boundary (34 mya) coincided with climate changes from warm and humid to colder and drier conditions (Prothero & Heaton, 1996; Meng & McKenna, 1998; Hassanin & Douzery, 2003). The divergence of major ruminant lineages has occurred within a very short period of time since their origination and ruminant evolution rates were not constant through time (DeMiguel, Azanza & Morales, 2014). From the Oligocene to the mid Pliocene global climatic and vegetational changes led to several successive rapid radiations within Pecora with additional short-termed diversification events within Bovidae and Cervidae (Hernández Fernández & Vrba, 2005). This rapid cladogenesis and parallel evolution may explain the lack of resolution or taxon instability in ruminant topologies and the plethora of convergent morphological developments (Hernández Fernández & Vrba, 2005; Janis & Theodor, 2014).

From the Oligocene to the Miocene cooler and more arid climate led to the replacement of forest habitats with open grasslands in Asia favouring the diversification and dispersal of many pecoran groups (Meijaard & Groves, 2004; Lorenzini & Garofalo, 2015). C3 grass dominated habitats occurred around 22 mya, C4 grass expanded around 17.5 mya (DeMiguel, Azanza & Morales, 2014). These conditions were perfect for the origin and diversification of Cervidae and other ruminant groups. The resulting competition of overlapping habitats of grazers and browsers must have played a crucial role in the evolution of Cervidae (Gilbert, Ropiquet & Hassanin, 2006).

At the Oligocene-Miocene boundary, the first cervoids appeared diverging from Oligocene taxa like *Dremotherium* or *Bedenomeryx* (Ludt et al., 2004). The antlerless *Dremotherium* from the early Miocene of Europe has been suggested as the earliest member of cervids (Brooke, 1878; Ginsburg & Heintz, 1966; Vislobokova, 1983). It shares morphological traits with cervids and moschids (Pomel, 1853; Costeur, 2011). *Dremotherium* was consistently found to be more similar to cervids and together with *Amphitragulus* is now widely considered to be a stem-cervoid or belonging to the so called Cervidomorpha (Heintz et al., 1990; Gentry, Rössner & Heizmann, 1999; Sanchez, Domingo & Morales, 2010; Sánchez et al., 2015). In the analyses here, *Dremotherium feignouxi* was most often placed in an unresolved position, confirming its controversial affinities.

Although Central Asia/Eastern Eurasia has been long regarded as the centre of origin and evolution of Cervidae (Vislobokova, 1990; Groves, 2006), evidence from the fossil record indicated that the origin of cervids may be in Europe (Heckeberg, 2017b). Their past diversity is known from around 26 fossil genera (Dong, 1993). Gilbert, Ropiquet & Hassanin (2006) reconstruction of the ancestral cervine, which was reconstructed to have had antlers with three tines, sexual dimorphism, moderately sized upper canines (smaller than in muntjacs), and a deep lacrimal fossa, cannot be confirmed by the fossil record.

The earliest cervids are from the mid early Miocene (MN3) represented by *Procervulus*, *Ligeromeryx* and *Acteocemas* and became more numerous and widely distributed during the Miocene. In the late early and early middle Miocene *Stephanocemas*, *Heteroprox*, *Lagomeryx* and *Dicrocerus* appeared (Ginsburg & Azanza, 1991; Dong, 1993). A low cervid diversity is assumed during the late Miocene and all typical Miocene cervids became extinct before the late Miocene (Ginsburg & Azanza, 1991; Böhme et al., 2012).

In the early Miocene geographical changes played an important role by opening migration routes in Europe, Asia, and Africa. This had an rapid increase of ungulate diversity as a consequence, which remained like that during the warm climate of the Miocene Climatic Optimum throughout the middle Miocene. During the Miocene forest habitats were replaced by grasslands, which favoured the greatest radiation of ruminants (Hassanin & Douzery, 2003). Stadler (2011) showed that there was a slight but not significant increase in the diversification rate of mammals 15.85 mya. Around 15 mya, the sea-levels fell due to cooling climate in the high latitudes and forming ice sheets in the Eastern Antarctic; the fallen dry areas became grasslands (Haq, Hardenbol & Vail, 1987; Flower & Kennett, 1994; Miller, Wright & Fairbanks, 1991; Ludt et al., 2004).

The climate further cooled causing colder winters and drier summers when the circulation of warm deep water between the Mediterranean and the Indo-Pacific was interrupted. Subsequently grasslands spread over Europe and Asia between 8 and 7 mya providing perfect conditions for ruminants to further diversify (Ludt et al., 2004).

The cooling climate and increased seasonality in the late Miocene likely played a crucial role in the decline of large mammal diversity and causing endemism to occur in the climate belts. The lower diversity and the endemism of today may have originated already in the late Miocene (12 mya) and may be more complex than assumed (to lay in the Quaternary Climatic Cycles) (Costeur & Legendre, 2008). In the late Miocene the temperature gradient from equator to pole was weak and higher latitudes were warmer than today (Micheels et al., 2011).

During the Late Miocene of Asia environmental changes and uplift of the Tibetan plateau (11–7.5 mya; Amano & Taira (1992)) coincided with a global increase in aridity, seasonality and subsequent spread of grassland in Asia (Flower & Kennett, 1994; Gilbert, Ropiquet & Hassanin, 2006). A glaciation period at the Miocene/Pliocene boundary caused a drop in sea levels triggering further diversification particularly within cervids (Ludt et al., 2004). A crucial factor for South East Asian cervid evolution was the split of the Indochinese and Sundaic faunistic subregions caused by high sea levels, which cut through the Thai/Malay Peninsula during the Early Pliocene separating faunas for the duration of around 1 my (Woodruff, 2003; Meijaard & Groves, 2004). After the warm Middle Pliocene, the Pliocene–Pleistocene boundary was characterised by drastic cooling (2.4–1.8 Ma) (Meijaard & Groves, 2004).

There is broad consensus that ancestral odocoileine cervids entered America from Siberia via the Bering Strait in the late Miocene/early Pliocene (Gustafson, 1985; Webb, 2000; Merino, Milne & Vizcaíno, 2005). The Bering land bridge disappeared around 9,000 years ago with rising sea levels and the formation of the Bering Sea ending the faunal exchange between American and North Asia (Ludt et al., 2004). It is assumed that their ancestors were Eurasian Pliocene deer with three-tined antlers, such as *Cervavitus* (Fry & Gustafson, 1974; Gustafson, 1985). The first (presumed) odocoileine taxa were *Eocoileus* from Florida and *Bretzia* from Nebraska (around 5 my old), which are similar to *Pavlodaria* from Northeastern Kazakhstan (Fry & Gustafson, 1974; Vislobokova, 1980; Gustafson, 1985; Webb, 2000; Gilbert, Ropiquet & Hassanin, 2006).

The split between Odocoileini and *Rangifer* was suggested to have occurred in the middle Miocene between 15.4 and 13.6 mya, although their origins and relationships are unknown; the presence of close relatives of *Rangifer* among South American odocoileine fossils from the Pleistocene has been suggested (Groves & Grubb, 1987; Douzery & Randi, 1997). Cervids migrated from North to South America via the Panamanian bridge 2.5 mya (Plio-Pleistocene boundary) (Webb, 2000; Merino, Milne & Vizcaíno, 2005). The split of Odocoileini into Blastocerina and Odocoileina was dated to around 3.4 mya. It was hypothesised that there was a diversification within Odocoileini in North America 5.1 mya, which is also supported by the fossil record (Vrba & Schaller, 2000; Gilbert, Ropiquet & Hassanin, 2006; Hassanin et al., 2012). The first unambiguous adult antler fragment of *Odocoileus* is from 3.8 to 3.4 mya (Gustafson, 1985). The polyphyletic split of the *Mazama* species into the two subclades, Blastocerina and Odocoileina, led to the interpretation that South America was colonised at least twice. First, by the ancestor of Blastocerina in the Early Pliocene (4.9–3.4 mya), although this cannot yet be confirmed by the fossil record nor by a certain presence of a connexion between North and South America. However, a much earlier closure of the Panama Isthmus between 15 and 13 mya was recently suggested (Montes et al., 2015). The second colonisation was by the ancestor of *Mazama americana* and *Odocoileus virginianus* around the Plio-/Pleistocene boundary Gilbert, Ropiquet & Hassanin (2006). Stadler (2011) reported a significant rate shift of speciation to a decreasing diversification rate at 3.35 mya, which coincides with high tectonic activity.

Hershkovitz (1982) assumed a small odocoileine ancestor living in North, Central, or South America during the Miocene–Pliocene-boundary from which *Mazama* and *Pudu* diverged. This hypothesis suggested an increase in body size over time in other odocoileines, which is in contrast to the traditional view of secondarily dwarfed *Mazama* and *Pudu*. As a logical consequence, the existence of medium sized forms during the late Miocene and Pliocene of Asia and North America was assumed, which would be the ancestors of the small odocoileines. This is also supported by the fossil record (Webb, 2000). Slightly differently, Merino & Rossi (2010) hypothesised that the first deer entering South America were medium sized with branched antlers; these presumably diverged into *Mazama* and *Pudu* with simpler antlers, most likely independently from each other.

Six fossil cervid genera are known from South America; they include *Agalmaceros* (1.8–0.8 mya), *Charitoceros* (1.8–subrecent), *Antifer* (1.2–subrecent), *Epieuryceros* 1.2–subrecent, *Morenelaphus* 0.5–subrecent, and *Paraceros* (0.5–0.2 mya) (Hoffstetter, 1952; Tomiati & Abbazzi, 2002; Merino, Milne & Vizcaíno, 2005; Merino & Rossi, 2010; Gonzalez et al., 2014). Their fossil record is scarce and thus, the validity of some of the species is doubtful (Alcaraz & Zurita, 2004; Menegaz, 2000; Merino & Rossi, 2010). So far, there are only few studies on extinct neotropical cervids and even fewer attempting to reconstruct the phylogeny of fossil and extant neotropical deer (Gutiérrez et al., 2015; Escobedo-Morales et al., 2016; Gutiérrez et al., 2017).

Neotropical cervids diversified after migration into South America, where they filled niches, which are occupied by bovids on other continents, making them the most diverse group of ungulates in South America (Gilbert, Ropiquet & Hassanin, 2006; Merino & Rossi, 2010). The low resolution among Odocoileini haplotypes also suggests a radiation event dating to about 2.5 mya, which coincides with the land mammal invasion from North to South America (Webb, 2000; Gilbert, Ropiquet & Hassanin, 2006). Today’s South American cervids are adapted to a wide range of ecological habitats (Merino, Milne & Vizcaíno, 2005). The radiation most likely was influenced by the absence of other ruminant artiodactyls and appears to be the opposite scenario as in Africa, where bovids dominated. Morphology, physiology, adaptation of the digestive system, temporal and spatial distribution of vegetation, and physicochemical properties of plants triggered the diversification, thus making the evolutionary patterns very complex (Merino & Rossi, 2010).

High diversification rates could be one reason for the difficulties in resolving their relationships. After decades of research, the taxonomy and evolutionary history of South American cervids remains enigmatic, partly because of the scarce Plio- and Pleistocene fossil record (Fry & Gustafson, 1974; Webb, 2000).

### Systematics of Fossil Cervids

#### Miocene Cervids

It was suggested to put *Lagomeryx*, *Procervulus*, *Heteroprox*, *Euprox*, *Dicrocerus*, *Stephanocemas* into a subfamily as a ‘primitive’ clade within Cervidae (Azanza, 1993b; Ginsburg, 1985; Rössner, 1995). Miocene cervids were usually considered to be distant from crown cervids representing a distinct group of stem cervids. They were subdivided into Lagomerycinae(/-dae), Procervulinae(/-dae) and Dicrocerinae(/-ini). All of them were regarded as sister clades to Cervidae (Mennecart et al., 2016). It was suggested that *Lagomeryx*, *Ligeromeryx*, and *Paradicrocerus* form the lagomerycines (lagomerycids in the original), *Heteroprox* and *Procervulus* form the procervulines, and *Acteocemas*, *Stehlinoceros*, and *Dicrocerus* form the dicrocerines (Gentry, Rössner & Heizmann, 1999). In none of the analyses here this split into three groups was distinctive. So far, not many attempts to reconstruct the phylogeny of Miocene cervids have been made (Azanza Asensio, 2000). Recently, Mennecart et al. (2016, 2017) presented the first phylogenetic analyses based on inner ear characters for several fossil cervids.

In the phylogenetic analyses here, Miocene cervids were most often placed either between the outgroup and all other cervids, mostly unresolved; sometimes a few taxa formed a clade. The placement between the outgroup and other cervids was expected from their temporal distribution and their shared higher proportion of plesiomorphic characters. The systematic relationships within early Miocene cervids have been and still are controversial. (Rössner, 1995; Azanza, Rössner & Ortiz-Jaureguizar, 2013).

##### Lagomeryx parvulus and ligeromeryx praestans

Qualitative morphological comparisons, especially on antler morphology, suggest that *Lagomeryx parvulus* and *Ligeromeryx praestans* and also *Paradicrocerus* (not included here) are closely related to each other. Only one analysis (cranial data set) here supports the sister taxon relationship of the former two taxa. Based on external comparative morphology of the cranial appendages, it was found that lagomerycines had antlers, which were shed (Heckeberg, 2017b). Therefore, they are included in Cervidae and a subfamily Lagomerycinae would be justified based on morphological qualitative comparisons, but is not strongly supported in the topologies. Data completeness or presence of specific characters that are absent in the other taxon could be the reasons for this. Also, whether lagomerycines(-ids) form a family as the sister taxon to Cervidae could not be entirely ruled out, but the tendency of *Ligeromeryx*, *Lagomeryx*, and *Palaeoplatyceros* to form a clade within a clade consisting of Miocene taxa (Figs. 14 and 15) indicates that lagomerycines form a subfamily of Cervidae in a stem position.

The systematic position of lagomerycines, has always been controversial. They have been considered as a family between Giraffidae and Cervidae (De Chardin, 1939), as part of the superfamily Cervoidea (Romer, 1966; Viret, 1961; Young, 1964), as a separate subfamily within Cervidae (Vislobokova, Changkang & Sun, 1989), as a family of aberrant giraffoids, as a junior synonym of Palaeomerycidae (Pilgrim, 1941; Simpson, 1945; Young, 1964), as junior synonym of Muntiacini/-ae (Chow & Shih, 1978), as more closely related to Antilocapridae (Ginsburg, 1985; Solounias, 1988), or as representing an entirely independent clade (Bubenik & Bubenik, 1986; Azanza, 1993b; Azanza & Ginsburg, 1997).

The discussions on the taxon in the literature and the new insights resulting from the analyses here clearly show that the systematic position of Lagomerycinae represents one of the most controversial of ruminant families, so far without unambiguous consensus. However, cranial and postcranial morphology and particularly the presence of antlers support the affiliation as stem Cervidae (Chow & Shih, 1978; Leinders & Heintz, 1980; Vislobokova, Changkang & Sun, 1989; Azanza & Ginsburg, 1997; Heckeberg, 2017b; Mennecart et al., 2017).

##### Procervulus dichotomus and procervulus praelucidus

In most analyses here, *Procervulus* was placed in a stem position and *Procervulus* and *Dicrocerus* were more closely related to each other than to other cervids. A sister taxon relationship of *Procervulus* and *Heteroprox* was not observed. In the combined morphological and TE analyses, a close relationship of *Procervulus dichotomus* and *Procervulus praelucidus* to *Dicrocerus elegans* was confirmed.

*Procervulus* was assumed to be the Miocene descendant of *Amphitragulus* and *Dremotherium* (Gentry, 1994; Rössner, 1995). Presumably, transitional forms existed, which were not documented in the fossil record (Rössner, 1995). *Procervulus* has often been hypothesised to be the sister taxon to all other cervids (Janis & Scott, 1987; Groves, 2007). In previous studies, *Procervulus* was placed as the sister taxon to *Heteroprox* Azanza Asensio (2000), Mennecart et al. (2016) and both were the sister taxon to the clade containing *Dicrocerus elegans*. In Mennecart et al. (2017) *Procervulus dichotomus* was the sister taxon to *Heteroprox larteti* and *Procervulus praelucidus* the sister taxon to both of them; this clade was placed between *Lagomeryx parvulus* and all other cervids, which is similar to the results here.

##### Heteroprox larteti

In the analyses here, *Heteroprox larteti* was most often placed in an unresolved position, between the outgroup and cervids, as the sister taxon to *Euprox furcatus* or *Dicrocerus elegans*, or in a clade with other Miocene taxa (morphology, TE). Some topologies indicated a potential closer relationship to Muntiacini based on apomorphic characters, similar to *Euprox furcatus*.

*Heteroprox* was assumed to be the descendant of *Procervulus* (Rössner, 1995). In Azanza Asensio (2000) *Heteroprox* was most often placed as the sister taxon to *Procervulus* or as an (unresolved) stem lineage. Similarly, in Mennecart et al. (2017) *Heteroprox larteti* was the sister taxon to *Procervulus dichotomus*.

##### Dicrocerus elegans

In the analyses here, *Dicrocerus elegans* was most often placed closely related to *Procervulus*, sometimes as the sister taxon to *Heteroprox larteti*, or between the outgroup and cervids. Based on the results here and discussions in the literature, *Dicrocerus* is most certainly a stem cervid with affinities primarily to *Procervulus* and secondarily to other Miocene cervids. In a few analyses a potentially closer relationship to Muntiacini was observed.

Azanza, DeMiguel & Andrés (2011) suggested that *Dicrocerus* is a transitional form between the Procervulinae and crown Cervidae, which had also been hypothesised by Vislobokova (1990). In Azanza Asensio (2000) *Dicrocerus elegans* was placed as the sister taxon to *Acteocemas* and *Stehlinoceros* (=*Paradicrocerus*) and this clade was the sister taxon to all burr-bearing antlered cervids. In Mennecart et al. (2017) *Dicrocerus elegans* was the sister taxon to *Eostyloceros hezhengensis* in a sister taxon position to the crown cervids.

##### Euprox furcatus

In the TE analyses here, *Euprox furcatus* was most often placed in an unresolved position or as the sister taxon to *Heteroprox larteti*; in the TE analyses it was placed in a clade with other Miocene cervids. The results indicate that *Euprox furcatus* shares characters with other Miocene cervids. However, antler and pedicle morphology is apomorphic and resembles that of extant Muntiacini.

It was suggested that modern *Muntiacus* and fossil muntiacines such as *Eostyloceros*, *Metacervulus* and *Paracervulus* diverged from *Euprox* (Vislobokova, 1990; Croitor, 2014). *Euprox* was the first cervid with burr-bearing antlers and a pedicle inclination similar to that of muntjacs. Therefore, it has been suggested in several studies that *Euprox* may be the earliest representative of crown cervids (Azanza, 1993b; Gentry, Rössner & Heizmann, 1999; Dong, 2007; Azanza, Rössner & Ortiz-Jaureguizar, 2013; Mennecart et al., 2016, 2017). It was often considered as a member of Muntiacini, which would imply that Muntiacini is the sister taxon to all other cervids. In Azanza Asensio (2000), *Euprox* is variably placed closely related to *Amphiprox*, to extant *Muntiacus* and *Elaphodus*, to *Eostyloceros*, or to *Metacervulus*, or as the sister taxon to a clade containing all five of the above species or a subset thereof. In Mennecart et al. (2016), *Euprox furcatus* was placed as the sister taxon to *Cervus elaphus*. They further stated that *Dicrocerus elegans*, *Euprox furcatus* and *Cervus elaphus* differ from the other Miocene cervids, that is Procervulinae, in certain inner ear characters; *Euprox furcatus* had the most derived characters among them. In Mennecart et al. (2017) *Euprox furcatus* was placed as the sister taxon to all crown cervids.

There is a large temporal gap in the early putative fossil muntjac-like cervid lineage between the first representatives, *Euprox*, and the presumed direct ancestors of muntiacines, for example *Eostyloceros* (Azanza Asensio & Menendez, 1989; Azanza, 1993b), and additionally an even larger gap between those early fossils and the first members of extant *Muntiacus*, which appear in the Pleistocene. For more certainty of the systematic relationships it would be crucial to find more fossil material that would link the early presumed muntiacines with the crown muntiacines.

##### Palaeoplatyceros hispanicus

In most analyses here *Palaeoplatyceros hispanicus* was placed between the outgroup and cervids, as the sister taxon to *Lagomeryx parvulus* or as the sister taxon to most other Miocene taxa. *Palaeoplatyceros* is highly incomplete and has a combination of plesiomorphic traits and apomorphic traits, such as ‘presence of a burr’.

*Palaeoplatyceros hispanicus* can be distinguished from all other contemporaneous cervid species based on the palmation of antlers; however, its systematic position is problematic (Azanza Asensio, 2000). In Azanza Asensio (2000), *Palaeoplatyceros* was mostly placed as the sister taxon to all other cervids with burr-bearing antlers. Unless more material becomes available, its systematic position will remain controversial. Based on the analyses here, *Palaeoplatyceros* is likely a stem cervid with burr-bearing antlers.

##### Pliocervus matheronis

*Pliocervus matheronis* is known from the Messinian (upper Turolian, MN13). In the analyses here, *Pliocervus matheronis* was most often placed in an unresolved position, mostly between the outgroup and cervids and sometimes related to other Miocene taxa.

Although Simpson (1945) included Pliocervinae, comprising *Cervocerus*, *Cervavitus*, *Procervus*, and *Pliocervus*, which were regarded as the immediate crown Cervini precursors (Gentry, 1994; Groves, 2007), in Cervinae, others could not find any phylogenetic relationship of *Pliocervus* with Cervini/Cervinae (Petronio et al., 2007). Gentry, Rössner & Heizmann (1999) placed *Cervavitus* and *Pliocervus* among Cervoidea, whereas Azanza & Montoya (1995) and Azanza Asensio (2000) classified *Pliocervus* as Cervinae. It was suggested to be closely related to the holometacarpal *Cervavitus* within Pliocervini, which was included in Cervinae (Czyżewska, 1968; Vislobokova, 1990; Azanza Asensio, 2000).

The high morphological similarity of *Pliocervus matheronis* to the late Miocene *Pavlodaria orlovi* implies that these two genera could be closely related or possibly even synonymous. It was suggested that the subfamily Pliocervinae Symeonidis (1974), containing *Pliocervus* and *Pavlodaria* is a synonym of Capreolinae. In Azanza Asensio (2000) *Pliocervus matheronis* was variably placed and seems to have the highest proportion of apomorphic characters compared to other Miocene cervids. In most recent studies *Pliocervus* was regarded as incertae sedis (Croitor, 2014, 2018).

A definite morphological characterisation of *Pliocervus* is still missing and its systematic position remains controversial (Godina et al., 1962; Czyżewska, 1968; Korotkevich, 1970; Azanza Asensio, 2000; Petronio et al., 2007; Croitor, 2014). More and new morphological and biometric data are needed to solve the systematic relationships of ‘pliocervines’ (Di Stefano & Petronio, 2002).

##### Eostyloceros hezhengensis

*Eostyloceros hezhengensis* from the late Miocene of China was used for scoring characters (Deng et al., 2014). In the analyses here, *Eostyloceros hezhengensis* was most often placed in an unresolved position or within Muntiacini, suggesting that it is probably more closely related to muntjacs than to other cervids, which would support results from comparative morphology. Thus, *Eostyloceros hezhengensis* can be considered as a direct ancestor of muntjacs.

*Euprox* is considered as the direct ancestor of *Eostyloceros*, *Metacervulus*, and *Paracervulus*; after a change from subtropical to more temperate climate and *Euprox*-like cervids were replaced by representatives of *Eostyloceros* (Azanza Asensio & Menendez, 1989; Azanza, 1993b; Pitra et al., 2004). This lineage is assumed to lead to extant *Muntiacus* (Vislobokova, 1990; Croitor, 2014). In Azanza Asensio (2000), *Eostyloceros* was always closely related to *Muntiacus* and *Metacervulus*, while in Mennecart et al. (2017) *Eostyloceros hezhengensis* was not placed within Muntiacini but was the sister taxon to *Dicrocerus elegans*.

##### Pliocene and Plio-Pleistocene Cervids

There is no generally accepted classification of Plio- and Plio-Pleistocene cervids Pfeiffer (1999); however, for Villafranchian cervids (MN16) the following classifications were suggested: *Croizetoceros ramosus*, *Metacervocerus pardinensis*, *‘Cervus’ philisi*, *‘Cervus’ perolensis*, *Eucladoceros ctenoides* were considered as Cervini, *Arvernoceros ardei* as Megacerini, and *Libralces gallicus* (not included here) and *Procapreolus cusanus* were considered as Capreolinae.

In most morphological topologies here, Plio- and Pleistocene cervids were placed within crown cervids, sometimes forming subclades. Some Plio- and Pleistocene cervids were placed more closely related to extant Cervini. Most of them were nested in a clade together with Pleistocene cervids. In a few topologies the majority of Pliocene cervids were in an unresolved sister taxon position to all other Cervinae.

##### Cervus australis

In the phylogenetic analyses here, *Cervus australis* was most often placed in an unresolved position, sometimes closer to Muntiacini than to other cervids; it was also placed between the outgroup and cervids, as the sister taxon to *Eostyloceros hezhengensis* and *Praeelaphus etueriarum*, to *Hippocamelus bisulcus*, or *Muntiacus muntjak*. Based on qualitative morphological comparisons it is most likely a stem cervid, potentially closer to Muntiacini.

This species was originally described by De Serres (1832) and all known specimens are from Montpellier, France (Gervais, 1852; Czyżewska, 1959). Little further information is available in the literature concerning this species. Many entries point to muntiacines, for example *Paracervulus australis* (Gentry, 2005); however, there are no obvious similarities to muntiacines in the investigated specimens. Croitor (2018) also confirms an incertae sedis status for this cervid based on comparative morphology. Thus, the systematic position of *Cervus australis* remains uncertain.

##### Arvernoceros ardei

In the analyses here, *Arvernoceros ardei* was placed in an unresolved position, often close to or within Cervini. In some topologies it was placed as the sister taxon to *Metacervocerus pardinensis*, *Praeelaphus perrieri*, and *Metacervocerus rhenanus*. It was placed as the sister taxon to *Dama dama* in several topologies.

*Arvernoceros* was part of the first radiation of Cervinae/-i together with *Metacervocerus*, *Praeelaphus*, *Axis* and *Rucervus* (Croitor, 2014). The systematic position of *Arvernoceros ardei* has been subject to speculation for decades, its definition is still incomplete and affinities to other cervids unclear. Depéret (1884) found similarity to *Axis*, but no affiliation to *Dama*; it was suggested that it is most similar to Megacerini (Heintz, 1970; Vislobokova, 1990, 2012). *Arvernoceros ardei* was considered to be closely related to modern *Elaphurus* (De Chardin & Piveteau, 1930), declared as incertae sedis genus by Lister (1987), closely related to *Axis* Di Stefano & Petronio (2002), closely related to *Rucervus* (Croitor, 2009; Croitor, 2018). Despite some uncertainties in the morphological analyses, a closer relationship to *Dama dama* than to other cervids was suggested here.

##### Croizetoceros ramosus

In most of the analyses here, *Croizetoceros ramosus* was placed in an unresolved position; it was sometimes the sister taxon to *Procapreolus cusanus*, *Alces alces*, *Ozotoceros bezoarticus*, or *Odocoileus*. The results suggest a placement within Capreolinae and most likely within Odocoileini.

The antler morphology of *Croizetoceros ramosus* does not share similarities with any extant cervid species or with other cervid species from the Villafranchian (Heintz, 1970). Unfortunately, there is not much known about its skull morphology (Croitor, 2014). In Mennecart et al. (2017) *Croizetoceros* was placed as the sister taxon to Capreolinae.

##### ‘Cervus’ perolensis

In the analyses here, *‘Cervus’ perolensis* was placed in an unresolved position and as the sister taxon to several cervine taxa. Repeated placements within Cervini suggest that *‘Cervus’ perolensis* almost certainly belongs to Cervini and is likely closely related to and/or an ancestor of *Cervus*.

*‘Cervus’ perolensis*, *Metacervocerus rhenanus*, and *‘Cervus’ philisi* were found to be similar to each other and *‘Cervus’ perolensis* and *Metacervocerus pardinensis* were classified as *Pseudodama* Azzaroli (1953); Azzaroli & Mazza (1992a). Later, *‘Cervus’ perolensis* was considered as a descendant of *‘Cervus’ philisi* by Stefaniak & Stefaniak (1995). Spaan (1992), however, concluded that *‘Cervus’ philisi* and *‘Cervus’ perolensis* are junior synonyms of *Metacervocerus rhenanus* and should be renamed as such, which was supported by Pfeiffer (1999). If this were true, *‘Cervus’ philisi* and *‘Cervus’ perolensis* should come out in a similar systematic position as *Metacervocerus rhenanus*.

##### Procapreolus cusanus

In the analyses here, *Procapreolus cusanus* was placed between the outgroup and cervids, within Capreolinae, sometimes within Odocoileini, and as the sister taxon to both *Capreolus*. Thus, *Procapreolus cusanus* most likely belongs to Capreolinae and the previously suggested close relationship to *Capreolus* was confirmed in some analyses.

Despite the widely accepted assumption that *Procapreolus cusanus* is closely related to or even a direct ancestor of *Capreolus*, the origin of *Capreolus* within *Procapreolus* is still under debate (Lechner-Doll et al., 2001). Some authors hypothesise that it may be assigned to *Capreolus* rather than *Procapreolus* (Valli, 2010). Others place it in an intermediate position between lower Pliocene and Pleistocene *Procapreolus* species and extant *Capreolus* (Czyżewska, 1968; Heintz, 1970; Lechner-Doll et al., 2001).

##### Metacervocerus pardinensis

In the analyses here, *Metacervocerus pardinensis* was most often closely related to or within Cervini, which suggests that *Metacervocerus pardinensis* is a member of Cervini and probably a close relative and/or ancestor of *Cervus*.

The temporal distribution of *Metacervocerus pardinensis* suggests that it could be an ancestor of *‘Cervus’ philisi*. *Metacervocerus pardinensis* and *Metacervocerus rhenanus* have enough morphological differences to justify two different species (Spaan, 1992). Dietrich (1938) proposed that *Metacervocerus pardinensis* is synonymous with *etueriarum*, *perrieri*, *issiodorensis* and *rhenanus*. Based on similarities to *Rusa* deer, the genus *Metacervoceros* was erected to represent European rusine deer (Croitor, 2006a). However, their systematic position remained controversial. *Metacervocerus pardinensis* was classified as *Pseudodama* by Azzaroli & Mazza (1992a), while De Vos & Reumer (1995) assigned *Metacervocerus pardinensis* and *Metacervocerus rhenanus* to *Cervus*, Pfeiffer (1999) to *Dama*, and Di Stefano & Petronio (2002) to *Rusa*. Differences in the skull morphology suggest that *Metacervocerus* does not belong to the *Cervus–Rusa* evolutionary lineage, which needs stronger evidence from the fossil record. Croitor (2014) suggested it is more likely that *Metacervocerus pardinensis* represents an ancestor of *Dama*.

##### Praeelaphus perrieri

In the analyses here, *Praeelaphus perrieri* was placed close to or within Cervini, which suggests that *Praeelaphus perrieri* is a member of Cervini and probably closely related to and/or the ancestor of *Cervus*.

The teeth and postcranial material from *Praeelaphus perrieri* and *Eucladoceros* are indistinguishable; however, *Praeelaphus perrieri* and *Eucladoceros ctenoides* do not coexist in any of the known localities, although they occupy the same niches. The systematic relationships remained uncertain (Croitor, 2014). Already Portis (1920) proposed a new subgenus *Praeelaphus* for ‘*Cervus*’ *perrieri*, as well as for *C*. *avernensis*, *C*. *etueriarum* from the early Villafranchian (Croitor, 2014). *Praeelaphus perrieri* was considered as the earliest representative of *Cervus* in Europe by Di Stefano & Petronio (2002), however, even though it is an early cervine, there is no clear evidence that it is directly related to *Cervus* and it more likely represents an extinct lineage within the early cervine evolution (Croitor, 2014).

##### Praeelaphus etueriarum

In the analyses here, *Praeelaphus etueriarum* was placed between *Eostyloceros hezhengensis* and *Eucladoceros ctenoides*, as the sister taxon to *Metacervocerus rhenanus*, *Eostyloceros hezhengensis*, or *Eucladoceros ctenoides*. Placements as the sister taxon to the *Cervus*-clade and within Muntiacini suggest that *Praeelaphus etueriarum* belongs to Cervinae and most likely to Cervini.

There is consensus that *Praeelaphus* is a member of the early radiation of Cervini and *perrieri*, *warthae*, and *lyra* may be synonyms as they represent similar and contemporaneous cervids (see above) (Croitor, 2014). Heintz (1970) suggested that *Praeelaphus etueriarum* was established based on a juvenile *Praeelaphus perrieri*, which is yet to be proven.

##### Eucladoceros ctenoides

Here, *Eucladoceros ctenoides* was most often placed within Cervinae and/or Cervini. which also indicate a potentially close relationship to *Cervus*.

Most of the previously defined *Eucladoceros* species were synonymised with *Eucladoceros ctenoides* (Azzaroli & Mazza, 1992a; De Vos & Reumer, 1995; Pfeiffer, 1999; Croitor & Bonifay, 2001; Valli & Palombo, 2005). ‘*E*. *senezensis*’ has been suggested to be an ancestor of *Megaceroides* or *Megaloceros giganteus* in particular (Azzaroli & Mazza, 1992a, 1992b; Kuehn et al., 2005). Pfeiffer (2002) proposed that *Eucladoceros*, *Megaloceros* and *Cervus* form a group. Flerov (1952) suggested that *Eucladoceros* is an ancestor of *Alces*, which is not supported by others (Heintz, 1970; Croitor, 2014). The comb-shaped antler morphology is unique and more similar to *Cervus elaphus* or *Cervus albirostris* than to any other living cervid (N. Heckeberg, 2017, personal observation). Because upper canines in *Eucladoceros ctenoides* are absent it was interpreted that the genus most likely does not belong to the *Cervus*-*Rusa*-lineage (Croitor, 2014); instead, *Eucladoceros ctenoides* was hypothesised as a descendant of an early three-tined ancestor of *Axis* or *Metacervocerus* (Croitor, 2014). In Mennecart et al. (2017) *Eucladoceros ctenoides* was placed as the sister taxon to the *Cervus*-*Rusa*-clade, which confirms the results from the analyses here.

##### Metacervocerus rhenanus

In the analyses here, *Metacervocerus rhenanus* was mostly placed as the sister taxon to Cervini and/or within Cervinae, which suggests that *Metacervocerus rhenanus* is a member of Cervini and potentially is either a close relative and/or ancestor of *Cervus* or *Axis*.

The genus *Metacervocerus* was established by Dubois (1904) as *Cervus* (*Axis*) *rhenanus* for the small sized deer from Tegelen. Spaan (1992) synonymised *‘Cervus’ philisi* from Senèze with ‘*C*’. *rhenanus* based on dentition and antler morphology. Croitor & Bonifay (2001) assigned it to the genus *Metacervocerus*. Several three-tined cervids were described from the early Pleistocene of Europe (De Vos & Reumer, 1995); *Metacervocerus rhenanus* was considered to include ‘*C*’. *philisi*, ‘*C*’. *perolensis*, *C*. *ischnoceros* and *Pseudodama lyra* and *‘Cervus’ philisi* was suggested to be a junior synonym of *Metacervocerus rhenanus* (Azzaroli et al., 1988; Spaan, 1992). *Metacervocerus rhenanus* was hypothesised to be an ancestor of *Dama dama* (Pfeiffer, 1999; Di Stefano & Petronio, 2002); however, this hypothesis was ruled out by the coexistence of both genera in the early Pleistocene (Croitor, 2014).

From the analyses based on the present data sets, the synonymy of *‘Cervus’ philisi* and *‘Cervus’ perolensis* with *Metacervocerus rhenanus* could not be confirmed. All analyses placed the three taxa differently and not closely related to each other. This may be caused by the differing availability of characters for each taxon and should be tested based on exclusively overlapping characters.

#### Pleistocene Cervids

In the early Pleistocene, Pliocene forms were successively replaced by more modern cervids. By the middle Pleistocene, most Pliocene and some early Pleistocene cervids became extinct, while extant representatives appeared (Dong, 1993).

Pleistocene cervids are more similar to extant forms. In the morphological topologies, similarly to the Plio- and Plio-/Pleistocene cervids, the Pleistocene cervids were distributed across crown group clades, sometimes forming subclades. The majority of Pleistocene cervids were placed closely related to Cervini.

##### ‘Cervus’ philisi

In the analyses here, *‘Cervus’ philisi* was most often placed within Cervinae or Cervini sometimes within the extant *Cervus*-clade, which suggests that *‘Cervus’ philisi* belongs to Cervini with a potentially closer relationship to *Cervus*. The results further support previous findings that *‘Cervus’ philisi* cannot be assigned to any extant cervid (except maybe *Cervus nippon*). *‘Cervus’ philisi* together with *Praeelaphus perrieri* potentially represents an extinct clade leading to *Cervus*. The suggested synonymy of *Metacervocerus rhenanus*, *‘Cervus’ philisi*, and *‘Cervus’ perolensis* could not be supported in the analyses.

In the past, *‘Cervus’ philisi* was considered to be related to *Axis* (Depéret & Mayet, 1911), to *Rusa* (Stehlin, 1923; Viret, 1954), and to *Cervus nippon* (Schaub, 1941). Heintz (1970) suggested an evolutionary *Metacervocerus pardinensis*-*‘Cervus’ philisi*-*‘Cervus’ perolensis*-lineage. However, the temporal occurrence of these species in the fossil record contradicts this hypothesis. It was suggested that *‘Cervus’ perolensis* is the descendant of *‘Cervus’ philisi* (Stefaniak & Stefaniak, 1995; Croitor, 2006a, 2014) and that *Metacervocerus rhenanus* from Tegelen and *‘Cervus’ philisi* from Senèze are synonymous and that *‘Cervus’ philisi* and *‘Cervus’ perolensis* are junior synonyms of *Metacervocerus rhenanus* (Spaan, 1992). Later, *‘Cervus’ philisi* was included in the genus *Metacervocerus* (Croitor & Bonifay, 2001; Croitor, 2006a). In Mennecart et al. (2017) *‘Cervus’ philisi* was placed closely related to *Axis* and *Rucervus duvaucelii*.

##### ‘Cervus’ sivalensis

The remains of *‘Cervus’ sivalensis* resemble *Rucervus duvaucelii* in morphology and size and *Rucervus eldii* in antler morphology (Azzaroli, 1954). Here, *‘Cervus’ sivalensis* was placed as the sister taxon to *Megaloceros giganteus* to a clade consisting of *Axis lydekkeri*, *Rusa kendengensis* and *Metacervocerus pardinensis* to *Metacervocerus pardinensis*, to the *Elaphurus*, or in a polytomy with *Metacervocerus pardinensis* and *Cervus canadensis* within the *Cervus*-clade. The placements within Cervini and close to the *Cervus*-clade show that *‘Cervus’ sivalensis* belongs to Cervini and is most likely closely related to *Cervus*, *Rusa*, and/or *Rucervus*. Together with *Axis lydekkeri* it could belong to the ancestral group of cervids that leads to *Axis*, *Cervus*, *Rusa* and *Rucervus*. Although the tooth morphology of *‘Cervus’ sivalensis* resembles that of *Rucervus* (N. Heckeberg, 2017, personal observation), a placement closely related to *Rucervus* could not be found. There is still a lot of confusion concerning the taxonomy and systematics of this taxon and a revision is needed (Lydekker, 1884; Azzaroli, 1954; Arif, Shah & Vos, 1991; Samiullah & Akhtar, 2007).

##### Axis lydekkeri

Even though *Axis lydekkeri* is a fairly complete fossil and despite the morphological similarities to *Axis*, *Axis lydekkeri* was not placed as closely related to extant *Axis* in the analyses here, instead it was mostly placed as the sister taxon to or within Cervini, or within the *Cervus*-clade, which shows that *Axis lydekkeri* belongs to Cervini.

*Axis lydekkeri* was suggested to be more closely related to the smaller *Axis* species of today (‘*Hyelaphus*’) than to *Axis axis* (Zaim et al., 2003; Meijaard & Groves, 2004; Gruwier, De Vos & Kovarovic, 2015), but a clear systematic relationship to any of them could not yet be confirmed.

##### Rusa kendengensis

In the analyses here, *Rusa kendengensis* was most often placed within Cervini and sometimes as the sister taxon to the *Cervus*-clade, which shows that *Rusa kendengensis* belongs to Cervini. Even though based on comparative anatomy it is more similar to *Rusa*, the analyses placed it more closely to *Cervus*. *Rusa kendengensis* potentially belongs to an extinct group of ancestors including also *Axis lydekkeri* and *‘Cervus’ sivalensis*, which gave rise to modern *Axis*, *Cervus*, and *Rusa*.

There is little information about *Rusa kendengensis* in the literature; it was suggested that it belongs to *Rusa* and not to *Cervus* as previously assumed for most Pleistocene cervids from Java (Dubois, 1908; Zaim et al., 2003). Recently, this was confirmed by morphometric analyses (Gruwier, De Vos & Kovarovic, 2015). More material of this species is needed to further investigate its systematic relationships.

##### Candiacervus ropalophorus

In the analyses here, *Candiacervus ropalophorus* was often placed close to several fossil cervine taxa and/or within Cervinae; in the SFA it was placed within Odocoileini. The investigated *Candiacervus ropalophorus* specimens were fairly complete; therefore, it was unexpected that this taxon was difficult to place. Frequent placements as the sister taxon to Cervini or within Cervini indicated that *Candiacervus ropalophorus* belongs to Cervini. The often hypothesised close relationship to megacerine/damine deer could only be found in one topology.

For *Candiacervus ropalophorus*, up to six different size groups representing six taxonomic units, sometimes even eight morphotypes have been suggested, but with differing views on the actual taxonomic affiliations Simonelli (1907, 1908*)*, Kuss (1975), Kotsakis & Palombo (1979), De Vos (1979, 1984, 2000) and Van der Geer et al. (2006). *Candiacervus ropalophorus* is the smallest species of the eight morphotypes. Since no cranial material can be unambiguously assigned to *Candiacervus cretensis* or *Candiacervus rethymnensis*, only *Candiacervus ropalophorus* can be considered as clearly recognisable species based on cranial and postcranial elements (De Vos, 1984).

The systematic position of *Candiacervus* is controversial; a close relationship to *Megaceros*, *Praemegaceros*, *Eucladoceros*, *Cervus*, or *Croizetoceros*, as has been suggested before (Kuss, 1975; De Vos, 1984). It remains difficult to determine the ancestor of the Greek island deer, and data are still insufficient to establish robust phylogenetic relationships of Cretan deer (Van der Geer et al., 2006).

##### Megaloceros giganteus

In the morphological analyses here, *Megaloceros giganteus* was placed in varying positions, within Cervinae, as the sister taxon to *Dama dama*, and often closely related to *Rangifer tarandus* (presumably due to similarities in antler morphology) . A close relationship to *Dama*, as strongly suggested by molecular analyses (Lister et al., 2005), is also supported in the TE BI and ML topologies. Together with the evidence from comparative morphology a close relationship of *Megaloceros giganteus* to *Dama* is almost certain.

There is a broad consensus today that *Megaloceros* consists of only one species, *Megaloceros giganteus* (Vislobokova, 1990, 2012, 2013; Azzaroli & Mazza, 1993; Croitor, Bonifay & Bonifay, 2006; Croitor & Bonifay, 2001; Croitor, 2014). All recent phylogenetic analyses consistently placed *Megaloceros giganteus* within Cervinae (Lister et al., 2005; Hughes et al., 2006; Vislobokova, 2009). In some studies *Megaloceros giganteus* was placed closely related to *Cervus elaphus* based on molecular data (Kuehn et al., 2005) and morphological data (Geist, 1998; Pfeiffer, 1999, 2002; Vislobokova, 2009). Lönnberg (1906) put it close to *Rangifer* because of a completely ossified vomer and palmated brow tines; however, it was found that the division of the nasal cavity is only ossified in the anterodorsal part of the vomerine septum, which is different from the condition in Capreolinae and presumably is a side effect of the cranial pachyostosis (Lister, 1994; Croitor, 2006b, 2014). Already Lydekker (1898) suggested an affiliation of *Megaloceros giganteus* to the damine group, which was supported in several subsequent studies using morphological, molecular or both types of data (Gould, 1974; Kitchener, 1987; Lister, 1994; Lister et al., 2005; Vislobokova, 2009). In the topology of Marcot (2007) *Megaloceros giganteus* was the sister taxon to all cervine taxa, and in Pfeiffer (2002) it was the sister taxon to two extant *Cervus*. In Mennecart et al. (2017) *Megaloceros giganteus* was the sister taxon to *Dama*.

##### Odocoileus

In the analyses here, both fossil *Odocoileus* specimens were most often placed as the sister taxon to odocoileine taxa, within Blastocerina, and sometimes to the other fossil *Odocoileus*.

The results for both fossil *Odocoileus* suggest that they are included within Capreolinae and within Odocoileini. However, only a few analyses placed them as sister taxa or closely related to their presumed living descendants *Odocoileus virginianus* and *Odocoileus hemionus*. Particularly the BSPG specimen was more often placed closely related to *Mazama* species. In Mennecart et al. (2017) the fossil *Odocoileus* BSPG specimen was placed in a trichotomy with the extant *Odocoileus* species.

##### Muntiacus

The fossil *Muntiacus muntjak* was often placed within Muntiacini, mostly as the sister taxon to *Muntiacus atherodes*. The results show that the fossil *Muntiacus* is certainly a member of Muntiacini.

### Systematics of extant cervidae

#### Cervid systematics in context of ruminant families

Decades of research demonstrated the difficulties of resolving the systematic relationships of the six ruminant families, especially among the pecoran families (Kraus & Miyamoto, 1991; Cronin et al., 1996; Randi et al., 1998; Cap, Aulagnier & Deleporte, 2002; Hassanin & Douzery, 2003; Hassanin et al., 2012). Particularly, the position of Moschidae, Antilocapridae and Giraffidae were problematic. Hassanin & Douzery (2003) and Price, Bininda-Emonds & Gittleman (2005) presented an overview of the systematic relationships of ruminants dating back to 1934.

However, recent molecular studies relatively consistently showed that the clade consisting of Moschidae plus Bovidae was the sister taxon to Cervidae, which was the sister taxon to Giraffidae, then Antilocapridae; Tragulidae was the sister taxon to all of them (Kuznetsova, Kholodova & Danilkin, 2005; Marcot, 2007; Agnarsson & May-Collado, 2008; Hassanin et al., 2012; Bibi, 2014; Zurano et al., 2019). The most recent study on ruminant genomics supports a sister taxon relationship of Antilocapridae & Giraffidae (Chen et al., 2019).

In the molecular topologies here, the systematic relationships among the six ruminant families varied. Most variation was observed in the nuclear markers. In most analyses, however, Moschidae and Bovidae were sister taxa to each other with Cervidae as the sister taxon, and Antilocapridae and Giraffidae as sister taxa to that clade, either unresolved or as clade.

One caveat that affects the apparent consensus on the systematic relationships among ruminant families is that it is often based on topologies repeatedly analysing the same types of data (e.g. mitochondrial DNA) with similar parameters. The results of Chen et al. (2019) partially support the consensus. Further work on morphological traits is needed to investigate the impact of inclusion of fossil taxa (Agnarsson & May-Collado, 2008; O’Leary & Gatesy, 2008).

Until recently, there were no comprehensive studies investigating the phylogenetic relationships of extant cervids based on morphology. Due to the highly conservative craniodental features of cervids, implications from the topologies based on morphology alone were limited. In the molecular topologies here, the systematic relationships of most clades above genus level were consistently recovered and well supported by different data sets. Many systematic relationships at genus- and/or species-level were also stable and were consistently placed on the same positions in topologies based on various molecular data sets. However, even though molecular data contributed to delimiting cervid clades and helped understanding the morphological evolution, some nodes remain unresolved or unstable. In the molecular and combined topologies, apart from a very few exceptions, Cervidae, Capreolinae and Cervinae were monophyletic; Cervini, Muntiacini, Odocoileini including *Rangifer* most often were monophyletic, too. The unstable position of Capreolini and Alceini questioned the monophyly of Capreolinae.

#### Cervini

The phylogenetic relationships of Cervini here, were similar to the results of recent molecular studies including Cervini; (Randi et al., 1998, 2001; Meijaard & Groves, 2004; Pitra et al., 2004; Hernández Fernández & Vrba, 2005; Gilbert, Ropiquet & Hassanin, 2006; Hughes et al., 2006; Marcot, 2007; Ouithavon et al., 2009; Hassanin et al., 2012; Heckeberg et al., 2016; Hu et al., 2019). The relationships within the subclades vary slightly depending on the taxon and character sampling.

There has been a long ongoing discussion about the genus and subgenus status of cervine taxa. In this study and in most of the recent literature (IUCN, 2019; Mattioli, 2011) six genera were distinguished: *Axis*, *Cervus*, *Dama*, *Elaphurus*, *Rucervus* and *Rusa*. *Przewalskium* was often listed as a seventh separate genus; however, extensive morphological investigation did not find enough difference for a separate genus status (N. Heckeberg, 2017, personal observation). *Elaphurus*, *Rucervus*, and *Rusa* are often considered as subgenera (Meijaard & Groves, 2004; Pitra et al., 2004; Gilbert, Ropiquet & Hassanin, 2006; Hassanin et al., 2012; Gruwier, De Vos & Kovarovic, 2015), but have many morphological distinctive features that justify separate genera (N. Heckeberg, 2017, personal observation).

##### Axis

The study of Meijaard & Groves (2004) was so far the only one to include the three species, *Axis porcinus* and *Axis kuhli*, for which molecular data were available. In the supertree analysis of Hernández Fernández & Vrba (2005) all four *Axis* species were included. *Axis* was not monophyletic in some studies (Pitra et al., 2004; Marcot, 2007; Agnarsson & May-Collado, 2008). This is most likely caused by re-analysing the same misidentified sequences (see discussion in Gilbert, Ropiquet & Hassanin (2006)).

In the analyses here *Axis* formed a well supported clade. *Axis axis* was always the sister taxon to the other two *Axis* species. Based on craniometrics and morphological similarities *Axis calamianensis*, *Axis kuhli*, *Axis porcinus* were considered to be closely related to each other and distinct from *Axis axis* (Meijaard & Groves, 2004). This was confirmed by the molecular and combined topologies here. In most of the topologies here *Axis* was closely related to *Rucervus*, which differs from the results in Pitra et al. (2004) and the supertree analysis in Hernández Fernández & Vrba (2005).

##### Cervus

The morphological analyses here, resulted in varying positions for the four *Cervus* species. All of them have a very similar cranial and dental morphology (N. Heckeberg, 2017, personal observation). In the nuclear analyses, *Cervus elaphus*, *Cervus canadensis* and *Cervus nippon* were more closely related to each other than to *Cervus albirostris*. In the mtG analyses *Cervus albirostris* and *Cervus nippon* formed a clade and *Cervus elaphus* was the sister taxon to them; if *Cervus canadensis* was included it was the sister taxon to *Cervus nippon* (and *Cervus albirostris*, if it was a trichotomy) and *Cervus elaphus* was the sister taxon to all of them. The same was found in the combined molecular and TE analyses. This is also confirmed in recent studies using mtDNA (Marcot, 2007; Hassanin et al., 2012; Heckeberg et al., 2016; Zurano et al., 2019).

In previous studies, *Cervus elaphus* was the sister taxon to *Cervus nippon* (Lister, 1984; Randi et al., 1998), or *Cervus nippon* was the sister taxon to *Cervus canadensis*, with *Cervus elaphus* and *Rusa* as the sister taxa to them (Randi et al., 2001; Pitra et al., 2004; Hughes et al., 2006). *Cervus canadensis* was the sister taxon to *Cervus nippon* with *Cervus albirostris* and *Cervus elaphus* as the sister taxon to all of them in Kuwayama & Ozawa (2000), Groves (2006) and Zachos et al. (2014). This contradicts results from traditional morphology, where *Cervus elaphus* and *Cervus canadensis* were usually sister taxa (Kuwayama & Ozawa, 2000). However, Polziehn & Strobeck (2002) stated that the divergence of mtDNA noted for *Cervus nippon*, *Cervus canadensis*, and *Cervus elaphus* is congruent with geographical, morphological, and behavioural distinctions.

In some studies, *Cervus albirostris* was the sister taxon to the other *Cervus* species (Hernández Fernández & Vrba, 2005; Hu et al., 2019); it was the sister taxon to *Cervus nippon*, with *Cervus canadensis* as the sister taxon to both and *Cervus elaphus* the sister taxon to all of them . In Agnarsson & May-Collado (2008) *Cervus albirostris* was the sister taxon to *Cervus elaphus*, and *Cervus nippon* to both of them. In contrast to this, Flerov (1952) suggested that *Cervus albirostris* diverged from *Rusa* in the late Pliocene and Koizumi et al. (1993) considered it more closely related to *Rucervus*. However, all recent molecular studies placed it closer to the *Cervus* species (Leslie, 2010). *Cervus albirostris* almost certainly evolved in temperate northern Eurasia; *Epirusa hilzheimeri* or *Eucladoceros* may have been its Pleistocene ancestors (Di Stefano & Petronio, 2002; Flerov, 1952; Zdansky, 1925; Geist, 1998; Grubb, 1990; Leslie, 2010).

The difference between mitochondrial and nuclear genes may indicate an ancient hybridisation event. It is known that hybridisation between *Cervus nippon* and *Cervus elaphus* (mainly *Cervus elaphus* females and *Cervus nippon* males) occurs and that hybrids are fertile. Hybridisation may lead to extensive introgression (Zachos & Hartl, 2011). Studies on population genetics and subspecies of red deer exclusively used mtDNA, which may suggest relationships that are not reproducible when using paternal genes. Hu et al. (2019) provide another insight into *Cervus* phylogeny using single nucleotide polymorphisms (SNP). Hybridisation could have occurred frequently in *Cervus*. The topologies here suggested varying sister taxon relationships across the four *Cervus* species.

##### Dama

In the analyses here, *Dama dama* and *Dama mesopotamica* were always sister taxa to each other and in most cases placed as the sister taxon to a clade consisting of *Cervus*, *Rusa*, *Elaphurus davidianus*, and *Rucervus eldii*. In previous studies, both *Dama* species were also sister taxa to each other (Randi et al., 2001; Lister et al., 2005; Hughes et al., 2006; Hassanin et al., 2012; Heckeberg et al., 2016; Zurano et al., 2019).

##### Elaphurus

In the nuclear analyses here, *Elaphurus davidianus* was mostly placed close to *Cervus*, while it was consistently placed as the sister taxon to *Rucervus eldii* in all mitochondrial, molecular combined, and TE analyses. In the morphological analyses it was placed closer to *Cervus* based on cranial characters and closer to *Rucervus* and *Rusa*, particularly *Rucervus schomburgki*, based on the dentition and the morphological combined data set.

The oldest known fossils of the *Elaphurus davidianus* lineage are known from the late Pliocene or slightly earlier (Taru & Hasegawa, 2002) and the first certain *Elaphurus davidianus* fossils date from the mid Pleistocene (Ji, 1985). The speciation of *Elaphurus* has been discussed as an ancient (late Pliocene or earlier) hybridisation event (Meijaard & Groves, 2004). *Cervus canadensis* or a closely related ancestor supposedly was the male parent and *Rucervus eldii* or a very close ancestral relative the female parent (Taru & Hasegawa, 2002; Meijaard & Groves, 2004; Pitra et al., 2004; Groves, 2006). The unique antler morphology and the overall phenotype of *Elaphurus davidianus* is distinct from all other cervids (Lydekker, 1898; Emerson & Tate, 1993; Meijaard & Groves, 2004; Pitra et al., 2004). Although some similarities to *Rucervus eldii* were stated (Meijaard & Groves, 2004), morphological scrutiny does not necessarily support that. The morphology of *Elaphurus* contains apomorphic character states and is not intermediate between its two parent taxa (Groves, 2014; N. Heckeberg, 2017, personal observation). This phenomenon is called transgressive segregation and the new phenotypes may be favoured in the new hybridogenetic population (Rieseberg, Archer & Wayne, 1999; Groves, 2014).

Because of this hybridisation molecular phylogenetic analyses result in conflicting systematic positions as clearly shown here, but also in earlier studies. Analyses of mitochondrial data placed *Elaphurus davidianus* as the sister taxon to *Rucervus eldii* (Randi et al., 2001; Pitra et al., 2004), while Electrophoretic patterns of 22 proteins and κ-casein DNA, and the karyotype placed *Elaphurus* closer to *Cervus* (Emerson & Tate, 1993; Cronin et al., 1996; Meijaard & Groves, 2004).

##### Rucervus

*Rucervus* species have a unique antler morphology and their teeth are uniquely folded indicating a specialisation for graminivory (Grubb, 1990; Meijaard & Groves, 2004); both provide useful morphological characters. The hypothesis that *Rucervus* is more closely related to *Rusa* than to *Cervus* was partly supported in the nuclear analyses and the morphological analyses here, while in the mitochondrial, molecular combined, and TE analyses *Rucervus* was polyphyletic with *Rucervus eldii* more closely related to *Elaphurus davidianus* and the other two species more closely related to *Axis*. Based on this it was suggested that *Rucervus eldii* may represent a different evolutionary lineage than the other two *Rucervus* species (Meijaard & Groves, 2004) and was sometimes put into a separate genus *Panolia* (Pocock, 1943; Groves, 2006). It is now widely regarded as *Rucervus eldii* (Wilson & Reeder, 2005; Timmins et al., 2008; Angom & Hussain, 2013). This is also supported by the topologies here, particularly the morphological topologies show the close relationship to the other two *Rucervus* species. The placement of *Rucervus eldii* separate from its two congeners in molecular topologies (especially mtDNA) is most likely artificially caused by the hybridisation of *Rucervus eldii* and *Cervus canadensis* in the past.

*Rucervus duvaucelii* and *Rucervus schomburgki* were sister taxa to each other in the analyses here and were mostly the sister taxon to *Axis*. The last specimen of *Rucervus schomburgki* became extinct in 1938. The first accounts on the species were by Blyth (1863), who noted the distinctive antler pattern. According to Gühler (1936), the geographical distribution of *Rucervus schomburgki* was restricted to Siam. It was assumed to be closely related to *Rucervus duvaucelii* and potentially interbreeding with *Rucervus eldii* in its natural habitat. The earliest fossils of *Rucervus* date back to 2.9 mya (Azzaroli et al., 1988; Meijaard & Groves, 2004).

##### Rusa

In the morphological analyses here, *Rusa* was more closely related to *Rucervus* (rarely to *Axis*). In the nuclear analyses, it was close to *Rucervus* or within Cervini, while it was more closely related to *Cervus* in the mitochondrial, combined molecular, and TE analyses. When all four *Rusa* were included, *Rusa timorensis* and *Rusa unicolor* were sister taxa to each other and to Cervus, and *Rusa marianna* and *Rusa alfredi* were sister taxa to each other and to all of the above. This was also found in recent studies (Hassanin et al., 2012; Heckeberg et al., 2016; Zurano et al., 2019) and confirms the controversial monophyly of *Rusa* (Meijaard & Groves, 2004; Hernández Fernández & Vrba, 2005; Randi et al., 2001; Leslie, 2011).

Thus, despite some new insights into the systematic relationships of *Rusa*, uncertainties remain. The Philippine *Rusa alfredi* and *Rusa marianna* share morphological similarities, and are distinct from the other two *Rusa* because of the overall smaller size. *Rusa unicolor* and *Rusa timorensis* from the mainland and Indonesia were considered to be more derived (Groves & Grubb, 2011), which is in contrast to the assumption that based on the high similarity of *Rusa unicolor* to pliocervines, an extinct lineage of Pliocene cervids, it is the most ancestral of the four extant rusine deer (Petronio et al., 2007; Leslie, 2011).

The first appearance of *R*. *unicolor* was recorded from the middle Pleistocene (Zong, 1987; Dong, 1993; Meijaard & Groves, 2004). The oldest *R*. *timorensis* is reported from the late Pleistocene (Van Mourik & Stelmasiak, 1986; Dong, 1993) and suggested to have then dispersed south-eastwards to Taiwan and Java (Meijaard & Groves, 2004).

#### Muntiacini

In the recent literature, muntiacines have been included in phylogenetic reconstructions to a different extent (Randi et al., 1998; Wang & Lan, 2000; Randi et al., 2001; Pitra et al., 2004; Hernández Fernández & Vrba, 2005; Gilbert, Ropiquet & Hassanin, 2006; Hughes et al., 2006; Marcot, 2007; Ouithavon et al., 2009; Hassanin et al., 2012). The systematic relationships within Muntiacini vary mostly depending on the taxon sampling, but do not contradict each other. The monophyly of Muntiacini uniting *Muntiacus* and *Elaphodus* has never been questioned Gilbert, Ropiquet & Hassanin (2006) and is supported by the analyses here.

##### Elaphodus

*Elaphodus cephalophus* was always the sister taxon to the other muntiacine species in all molecular and TE analyses presented here, which is also widely supported in the literature (Wang & Lan, 2000; Hernández Fernández & Vrba, 2005; Agnarsson & May-Collado, 2008; Hassanin et al., 2012). In contrast, in Marcot (2007) *Elaphodus cephalophus* is the sister taxon to all cervids.

*Elaphodus cephalophus* has the smallest known antlers, which are completely covered by tufts (Leslie, Lee & Dolman, 2013). Groves & Grubb (1990) considered *Elaphodus cephalophus* as the most primitive representative of living muntiacines. However, this is in contrast to the absence of fossils with such diminutive antlers. The first *Elaphodus* fossils are known from the Pleistocene of China, which were larger than *Elaphodus cephalophus*; therefore, the decrease in size can be considered as evolutionary trend in this species (Leslie, Lee & Dolman, 2013).

##### Muntiacus

All muntjacs have long pedicles, facial crests, and bifurcating antlers (N. Heckeberg, 2017, personal observation; e.g. Ma, Wang & Xu, 1986). In the morphological analyses here, muntiacine taxa were placed as the sister taxa to most other cervids or in an unresolved position. In most of the combined morphological analyses Muntiacini was monophyletic except for the BI analyses. In the MP analyses, Muntiacini were placed more closely related to other small cervids, such as *Mazama* and *Pudu*.

The earliest fossil of the *Muntiacus* lineage is *Muntiacus leilaoensis* from Yunnan, China and was dated to the late Miocene 9–7 mya (Dong, Pan & Liu, 2004). All *Muntiacus* species consistently formed a clade as the sister taxon to Cervini in the mitochondrial, molecular combined, and TE analyses here. A clade consisting of *Muntiacus crinifrons*, *Muntiacus feae*, and *Muntiacus muntjak* and a clade consisting of *Muntiacus putaoensis*, *Muntiacus truongsonensis*, *Muntiacus rooseveltorum*, *Muntiacus vuquangensis*, and *Muntiacus reevesi* were recovered in the mitochondrial and combined molecular analyses. *Muntiacus atherodes* was placed in a polytomy with these clades. In the TE analyses *Muntiacus reevesi* was placed between *Elaphodus cephalophus* and the other muntjacs and *Muntiacus atherodes* was the sister taxon to *Muntiacus feae*.

Several new muntiacine species have been discovered in the 1990s; subsequently, five to possibly six new muntjac species were established, *Muntiacus gongshanensis*, *Muntiacus crinifrons*, *Muntiacus feae*, *Muntiacus reevesi*, *Muntiacus muntjak* (Lan, Wang & Shi, 1995). Ma, Wang & Xu (1986) stated that *Muntiacus crinifrons* and *Muntiacus rooseveltorum* derived from *Muntiacus reevesi*, whereas *Muntiacus feae* and *Muntiacus muntjak* derived from a different lineage. The species status of *Muntiacus rooseveltorum* has been controversial for decades (Amato et al., 1999b); for example Groves & Grubb (1990) suggested that *Muntiacus rooseveltorum* is the synonym of *Muntiacus feae* and that *Muntiacus feae* is the sister taxon to *Muntiacus muntjak* and *Muntiacus crinifrons*. This is supported by most molecular studies and the topologies of this work. Sometimes, *Muntiacus crinifrons* and *Muntiacus gongshanensis* are considered as a single species (Amato et al., 1999b). It was proposed that *Muntiacus atherodes* should be included in *Muntiacus muntjak* based on morphological evidence, because the holotype of *Muntiacus atherodes* is a subadult male with single-tined antlers (Ma, Wang & Xu, 1986). The two specimens investigated here were indeed subadult individuals with not yet fully developed antlers (N. Heckeberg, 2017, personal observation). However, molecular topologies here and in the literature indicate a separate species status for *Muntiacus atherodes* (Heckeberg et al., 2016). The genus status of *Megamuntiacus* is not justified demonstrated by the sequence divergence estimated for the mitochondrial variation and by morphological comparisons; therefore, it is referred to as *Muntiacus* (Schaller, 1996; Giao et al., 1998; Amato, Egan & Rabinowitz, 1999a, Rabinowitz et al., 1999; Wang & Lan, 2000). Apart from the larger size, there are no morphological features that would justify a separate genus (N. Heckeberg, 2017, personal observation).

#### Alceini

##### Alces

*Alces* has a highly derived skull morphology with an elongated viscerocranial proportion and antlers that protrude horizontally. The dentition, particularly the lower premolars, shows similar modifications as in *Rangifer*. In the morphological analyses here, *Alces alces* was in an unresolved position or placed as the sister taxon to *Odocoileus hemionus*, *Mazama chunyi*, *Ozotoceros bezoarticus* or *Cervus canadensis*. In the mitochondrial, combined molecular and TE analyses *Alces alces* was consistently placed as the sister taxon to Capreolini, except for the BI combined molecular topology, where it was placed between Capreolini and Odocoileini plus *Rangifer*.

In most recent studies, *Alces* was placed as the sister to Capreolini (Randi et al., 1998; Pitra et al., 2004; Hughes et al., 2006; Agnarsson & May-Collado, 2008; Hassanin et al., 2012) or as the sister taxon to *Capreolus* (Hernández Fernández & Vrba, 2005). In Marcot (2007) *Alces* was the sister taxon to Capreolini and Odocoileini and Rangiferini, while it was in a polytomy with Odocoileini plus *Rangifer* and Capreolini or the sister taxon to Odocoileini plus *Rangifer* in Gilbert, Ropiquet & Hassanin (2006). More controversial positions included *Alces* as the sister taxon to Cervini or *Dama dama* in Kuehn et al. (2005) and the sister taxon position to *Rangifer* in Pfeiffer (2002). *Alces* was in a polytomy with Odocoileini and Rangiferini in Lister (1984) and took up variable positions in previous studies as summarised in Lister (1998). Thus, the systematic position of *Alces* remains unresolved.

The first *Alces alces* is known from the Riss glaciation 200–100 kya; those late Pleistocene moose were larger than their extant representatives (Franzmann, 1981).

##### Capreolini

Most analyses based on the combined morphological data set supported monophyletic Capreolini. However, the systematic position of Capreolini varied and could not be determined with certainty using morphological data only. In the molecular analyses here, Capreolini was always monophyletic and mostly placed closely related to or in most cases as the sister taxon to Odocoileini plus *Rangifer*.

Miyamoto, Kraus & Ryder (1990) suggested that Capreolini probably originated in the late Miocene in the Old World. The assumption of a late Miocene Old World origin of Capreolinae is in congruence with the findings here considering the placement of *Procapreolus*. Cronin (1991) hypothesised that *Alces* and *Rangifer* split earlier than the *Capreolus* lineage, but after the separation of Cervinae and Capreolinae.

##### Capreolus

In the morphological, molecular, and TE topologies *Capreolus capreolus* and *Capreolus pygargus* both species were consistently placed as sister taxa. In the mitochondrial, molecular combined and TE topologies, *Capreolus* was always the sister taxon to *Hydropotes* with strong support. Molecular studies of the past decades support the consistent placement of *Hydropotes* as the sister taxon to *Capreolus* forming monophyletic Capreolini (Douzery & Randi, 1997; Randi et al., 1998; Hassanin & Douzery, 2003; Pitra et al., 2004; Hughes et al., 2006; Gilbert, Ropiquet & Hassanin, 2006; Marcot, 2007; Agnarsson & May-Collado, 2008; Hassanin et al., 2012; Heckeberg et al., 2016; Zurano et al., 2019).

##### Hydropotes

Here, *Hydropotes* and *Capreolus* were sister taxa in the morphological combined, nuclear, mtDNA, molecular combined and TE analyses. In the past, *Hydropotes* was considered as a separate subfamily Hydropotinae as the sister taxon of all other cervids (Groves & Grubb, 1987; Janis & Scott, 1987; Hernández Fernández & Vrba 2005; Kuznetsova, Kholodova & Danilkin, 2005). Schilling & Rössner (2017) extensively reviewed the taxon. Already Bouvrain, Geraads & Jehenne (1989) favoured the hypothesis that *Hydropotes* and *Capreolus* are sister taxa. The first molecular studies indicated that *Hydropotes* is included in monophyletic Cervidae (Kraus & Miyamoto, 1991). From this follows that *Hydropotes* lost the antlers secondarily and developed enlarged upper canines as compensation (Douzery & Randi, 1997; Randi et al., 1998; Hassanin & Douzery, 2003).

Randi et al. (1998) demonstrated that the two *Capreolus* species and *Hydropotes* share a G at position 525 of *Cytb*, which occurs only rarely in other mammal species, and stated that ‘this replacement represents a nearly exclusive synapomorphy for the *Hydropotes*-*Capreolus*-clade’. Further, the telemetacarpal condition and a large medial opening of the temporal canal are morphological features that *Hydropotes* shares with other Capreolinae (Bouvrain, Geraads & Jehenne, 1989; Douzery & Randi, 1997; Randi et al., 1998). Behavioural characters also suggested that *Hydropotes inermis* is closely related to *Capreolus* (Cap, Aulagnier & Deleporte, 2002). Thus, increasing evidence from mitochondrial and nuclear DNA, morphology, and behaviour confirm a sister taxon relationship of *Hydropotes* and *Capreolus*.

#### Rangiferini

##### Rangifer

The systematic position of *Rangifer* was variable in the morphological analyses here. *Rangifer* has some apomorphic characters, not shared by other cervids, which is likely the cause of the difficulties to place the taxon based on morphology only. In the molecular and TE topologies *Rangifer tarandus* was consistently placed as the sister taxon to Odocoileini. This is supported by the most recent literature (Randi et al., 1998; Hassanin & Douzery, 2003; Pitra et al., 2004; Hernández Fernández & Vrba, 2005; Gilbert, Ropiquet & Hassanin, 2006; Hughes et al., 2006; Agnarsson & May-Collado, 2008; Duarte, González & Maldonado, 2008; Hassanin et al., 2012; Gutiérrez et al., 2017).

*Rangifer* appeared in the fossil record in the Pleistocene; based on its arctic specialisations it is hypothesised that it dispersed to America during the Pleistocene contemporaneously with *Alces* (Gilbert, Ropiquet & Hassanin, 2006).

#### Odocoileini

In the morphological topologies here most odocoileine taxa were in unresolved and/or variable positions; in some topologies the small odocoileine cervids were in a clade with muntiacine taxa. In the nuclear topologies, systematic relationships within Odocoileini were partly or entirely unresolved. In the mitochondrial, combined molecular, and TE topologies here, Odocoileini split into the two subclades Blastocerina and Odocoileina (Heckeberg et al., 2016).

In previous phylogenetic studies, the taxon sampling for Odocoileini varied greatly, therefore, it is difficult to compare the topologies (Douzery & Randi, 1997; Randi et al., 1998; Pitra et al., 2004; Hernández Fernández & Vrba, 2005; Hughes et al., 2006; Gilbert, Ropiquet & Hassanin, 2006; Marcot, 2007; Agnarsson & May-Collado, 2008; Duarte, González & Maldonado, 2008; Hassanin et al., 2012). In these studies, Odocoileini usually formed a monophyletic group with Rangiferini as the sister taxon to them. *Blastocerus dichotomus*, *Ozotoceros bezoarticus*, and *Pudu puda* were particularly unstable across studies with comparable taxon sampling. In the topologies here, they were sensitive to changes in the analysis parameters. Odocoileina and Blastocerina were sister taxa in several recent studies (Pitra et al., 2004; Hughes et al., 2006; Gilbert, Ropiquet & Hassanin, 2006; Marcot, 2007; Agnarsson & May-Collado, 2008; Hassanin et al., 2012; Heckeberg et al., 2016; Gutiérrez et al., 2017). This is also the case in Duarte, González & Maldonado (2008), but *Pudu puda* was in a polytomy to those clades. In addition, the results here and those of previous studies showed polyphylies for three odocoileine genera *Hippocamelus*, *Mazama* and *Pudu* and for both species of *Odocoileus* (Pitra et al., 2004; Gilbert, Ropiquet & Hassanin, 2006; Agnarsson & May-Collado, 2008; Duarte, González & Maldonado, 2008; Hassanin et al., 2012; Heckeberg et al., 2016; Gutiérrez et al., 2017). It remains uncertain, whether *Pudu* is monophyletic, polyphyletic within Blastocerina or polyphyletic with one species in Blastocerina and one species in Odocoileina. More morphological and molecular, particularly nuclear markers, and cytogenetic data are needed to reconstruct the complex evolutionary history of Odocoileini (Duarte, González & Maldonado, 2008; Hassanin et al., 2012; Gutiérrez et al., 2017).

##### Blastocerus

In the analyses here, *Blastocerus dichotomus* was positioned in an unresolved position based on morphological data and consistently placed within Blastocerina in the molecular and TE analyses. Most often it was positioned between *Pudu puda* (sometimes also *Mazama nemorivaga*) and the other Blastocerina. In previous studies *Blastocerus* took up variable positions, most likely depending on the taxon sampling. for example as the sister taxon to *Hippocamelus bisulcus* plus *Mazama gouazoubira* (Duarte, González & Maldonado, 2008), as the sister taxon to *Mazama gouazoubira* (Agnarsson & May-Collado, 2008), in a polytomy with *Mazama gouazoubira*, *Pudu puda*, *Hippocamelus antisensis* (Gilbert, Ropiquet & Hassanin, 2006), as the sister taxon to *Pudu puda* (Hughes et al., 2006), and as sister taxon to *Mazama nemorivaga* (Hassanin et al., 2012). Studies with a more extensive taxon sampling (Heckeberg et al., 2016; Gutiérrez et al., 2017) and the analyses of this work indicated a systematic position of *Blastocerus* as the sister taxon to most blastocerine species, with *Mazama nemorivaga* as the sister taxon to them and *Pudu puda* as the sister taxon to all other Blastocerina. A few analyses placed *Blastocerus* as the sister taxon to all other Blastocerina. These differing placements of *Blastocerus* most likely resulted from a differing taxon sampling.

The first *Blastocerus* fossils are known from the Pleistocene of Brazil and Paraguay. The populations in central Brazil most likely expanded between 28 and 25 kya and it was assumed that there were no geographical barriers until about 300 years ago (Merino & Rossi, 2010).

##### Hippocamelus

In several of the morphological topologies, both *Hippocamelus* species were monophyletic, sometimes with *Ozotoceros* as the sister taxon. Two of the four sequences for *Hippocamelus antisensis* formed a clade with *Hippocamelus bisulcus*, while the other two formed a clade with *Ozotoceros bezoarticus* (Heckeberg et al., 2016). This makes it almost certain that two of the four sequences are misidentified or mislabelled; a less likely possibility is that this polyphyly represents a valid split within the genus. Without knowing the exact provenance of the samples it cannot be determined which sequences are truly *Hippocamelus antisensis*. In the molecular combined and TE analyses here, those *Hippocamelus antisensis* mt-sequence(s) were included, with which the genus is monophyletic (Heckeberg et al., 2016). *Hippocamelus* was the sister taxon to *Mazama gouazoubira* (plus *Mazama chunyi*, if included).

Duarte, González & Maldonado (2008) stated that it is surprising that members of morphologically cohesive genera such as *Hippocamelus*, *Mazama*, or *Pudu* were not monophyletic based on molecular data. *Hippocamelus antisensis* and *Hippocamelus bisulcus* were found to be osteologically nearly indistinguishable (Flueck & Smith-Flueck, 2011; N. Heckeberg, 2017, personal observation). Based on this, a monophyly for *Hippocamelus* is more likely than a polyphyly as suggested by some of the molecular data. Thus, the potential polyphyly within *Hippocamelus* cannot be confirmed or ruled out yet; new sequences and more investigations are needed to clarify this phenomenon (see also discussion in Gutiérrez et al. (2017)).

The first *Hippocamelus bisulcus* is known from the late Pleistocene of Chile, Argentina, and Bolivia (Canto, Yáñez & Rovira, 2010; Merino & Rossi, 2010). *Odocoileus lucasi* is considered to be the ancestor of *Hippocamelus bisulcus*.

##### Mazama

Only little is known about the rarer *Mazama* species (and neotropical cervids in general), which represent the least studied organisms and many aspects of their life history are poorly understood (Duarte et al., 2012a, 2012b, 2012d, 2012e, 2012f; Lizcano et al., 2010; Gutiérrez et al., 2015, 2017). The first fossil *Mazama* are known from the Pleistocene of Argentina, Ecuador, Peru and Brasil (Merino & Rossi, 2010).

While the monophyly of *Mazama* has never been questioned based on morphological characters, previous molecular studies and the topologies here repeatedly showed polyphyletic relationships (Gilbert, Ropiquet & Hassanin, 2006; Duarte, González & Maldonado, 2008; Gutiérrez et al., 2015; Escobedo-Morales et al., 2016; Heckeberg et al., 2016; Gutiérrez et al., 2017).

Recent molecular studies showed that *Mazama americana* is a polyphyletic species, splitting into several lineages (Heckeberg et al., 2016; Gutiérrez et al., 2017). The genetic distance between the two *Mazama americana*-clades was higher than the genetic difference of *Mazama bororo* and *Mazama nana*; therefore, at least two species were assumed to be within the *Mazama americana*-complex, with a separate evolution of the two clades starting 1 mya and 2 mya, respectively (Duarte, González & Maldonado, 2008; Abril et al., 2010). The topology Gutiérrez et al. (2017) also shows the geographic distribution of the different *Mazama americana*-linegaes. *Mazama bororo* and *Mazama nana* were sister taxa to each other and are closely related to (this study) or nested within *Mazama americana* (Heckeberg et al., 2016; Gutiérrez et al., 2017). *Mazama temama* is the sister taxon to the *Mazama americana* group 1 (*sensu* Gutiérrez et al. (2017)), which was confirmed here. *Mazama pandora* was the sister taxon to *Odocoileus* here, which has also been found previously (Escobedo-Morales et al., 2016); a closer relationship to the *columbianus*-group of *Odocoileus hemionus* was found in (Gutiérrez et al., 2017), who suggested to assign *Mazama pandora* to the genus *Odocoileus*. *Mazama chunyi*, *Mazama gouazoubira*, and *Mazama nemorivaga* were consistently placed within Blastocerina, which confirms previous findings (Escobedo-Morales et al., 2016; Heckeberg et al., 2016; Gutiérrez et al., 2017). Already Duarte, González & Maldonado (2008) suggested that *Mazama gouazoubira* and *Mazama nemorivaga* should be assigned to a different genus. *Mazama chunyi* was independently found to be the sister taxon to *Mazama gouazoubira* based on two different *Mazama chunyi* sequences (Heckeberg et al., 2016; Gutiérrez et al., 2017), which was confirmed here. Therefore, Gutiérrez et al. (2017) suggested that *Mazama chunyi* and *Mazama gouazoubira* should be assigned to a genus different from *Mazama*. *Mazama nemorivaga* was the sister taxon to all other blastocerine taxa except for *Pudu* or nested unresolved within Blastocerina. Similar findings in Gutiérrez et al. (2017) led them to suggest that *Mazama nemorivaga* should be assigned to a different genus.

The low morphological diversity among *Mazama* is not correlated with the genotypic diversification, which leads to the problematic taxonomy; thus, a varying number of species were established based on different types of data (Groves & Grubb, 1987, 1990; Duarte & Merino, 1997; Duarte, González & Maldonado, 2008).

In the morphological analyses here most *Mazama* species were placed as closely related to each other most likely because of their small size and because they are morphologically almost indistinguishable (González et al., 2009; N. Heckeberg, 2017, personal observation). Gutiérrez et al. (2015) tested whether the degree of concavity of the dorsal outline in lateral view and the shape of the lacrimal fossa can distinguish *Mazama bricenii* and *Mazama rufina*, but found that these characters are too variable to discriminate species. In the molecular and TE analyses here, *Mazama bricenii* was placed as the sister taxon to *Mazama rufina*, while in Gutiérrez et al. (2015) and Gutiérrez et al. (2017) *Mazama bricenii* was nested within *Mazama rufina* and the opposite was the case in Heckeberg et al. (2016). Gutiérrez et al. (2015, 2017) stated based on their results that *Mazama bricenii* is not a valid taxon, but a junior synonym of *Mazama rufina*. Further, Gutiérrez et al. (2017) suggest to assign *Mazama rufina* and the *Mazama americana* group 2 to two different genera.

As discussed in Gutiérrez et al. (2017), the complex taxonomy of *Mazama* clearly needs a thorough revision, taking into account not only molecular data, but also (palaeo)biogeography, karyotypic and morphological data.

##### Odocoileus

In the morphological analyses based on the combined data set here *Odocoileus hemionus* was the sister taxon to , and in several topologies *Odocoileus virginianus* was the sister taxon to them. In all other morphological topologies, odocoileine taxa were placed in unresolved or varying positions. In the analyses including mitochondrial markers and a broad taxon sampling, both species were polyphyletic. In the analyses based on the nuclear markers, *Odocoileus* was not monophyletic in the topology based on the *Prnp* and *Prkci* marker and the combined nuclear analyses (Fig. 13; Supplemental Information 3).

Despite all the research undertaken on the genus, the taxonomy remains difficult. There are numerous subspecies (8–10 for *O*. *hemionus*, 37–38 for *O*. *virginianus*; Wilson & Reeder, 2005; Mattioli (2011)), which possibly, at least partly, represent separate species (Groves & Grubb, 2011; Gutiérrez et al., 2017).

Latch et al. (2009) demonstrated that there are two different morphotypes of *O*. *hemionus*, the mule deer and black-tailed deer, which is supported by a strong genetic discontinuity across the spatial distribution. Early investigations of mtDNA data demonstrated that *O*. *hemionus* is polyphyletic because the sequences of the mule deer (*O*. *hemionus*) and *O*. *virginianus* are more similar than the DNA of the black-tailed deer (*O*. *hemionus columbianus*) is to both of them (5–7% different) (Carr et al., 1986; Cronin, Vyse & Cameron, 1988; Cronin et al., 1996; Latch et al., 2009; Gutiérrez et al., 2017).

Similarly, the genetic divergence within *O*. *virginianus* is remarkably high, even higher than the genetic distance between other subspecies and between *O*. *virginianus* and mule deer. This led to the classification of white tailed deer into two distinct groups, the *cariacou*-division and the *virginianus*-division (Wilson, Carlson & White, 1977; Smith et al., 1986; Groves & Grubb, 1987; Grubb, 1990). *Odocoileus virginianus* is a highly plastic species occupying a great variety of geographically and ecologically extensive habitats between Canada and Peru, however, extreme habitat differences do not necessarily lead to large morphological divergence (Smith et al., 1986; Moscarella, Aguilera & Escalante, 2003; Merino & Rossi, 2010; Duarte et al., 2012c). Introgression seems to be the likely explanation because natural hybridisation and interbreeding between both species of *Odocoileus* have been documented (Groves & Grubb, 2011; Hassanin et al., 2012).

The first *Odocoileus* is from the early Pliocene (3.5 mya) of North America, where they were the most common cervids until the Pleistocene. *Odocoileus virginianus* appeared 2 mya presumably as the descendant of *O*. *brachyodontus*, which originated in Central America and dispersed to higher latitudes only recently (Hershkovitz, 1972; Smith, 1991; Merino & Rossi, 2010). It has been assumed that *Odocoileus virginianus* evolved in North America; it was further suggested that all South American cervid fossils belong to *Odocoileus* and that *Mazama* later diverged as a consequence of isolation within South America (Smith et al., 1986; Moscarella, Aguilera & Escalante, 2003). This is in contrast with the most recent molecular topologies (Escobedo-Morales et al., 2016; Heckeberg et al., 2016; Gutiérrez et al., 2017) and this work (Figs. 13 and 14), from which it appears that *Odocoileus* originated from the odocoileine *Mazama*-clade.

##### Ozotoceros

Similar to *Blastocerus*, the systematic position of *Ozotoceros* varied with the taxon sampling. With an extensive taxon sampling *Ozotoceros bezoarticus* was relatively consistently placed as the sister taxon to *Hippocamelus*, *Mazama gouazoubira* and *Mazama chunyi* (if included) in the analyses here. Other studies also demonstrated a variable position of *Ozotoceros bezoarticus* (Agnarsson & May-Collado, 2008; Duarte, González & Maldonado, 2008; Hassanin et al., 2012, Escobedo-Morales et al., 2016; Heckeberg et al., 2016; Gutiérrez et al., 2017; Zurano et al., 2019), which presumably is caused by varying taxon sampling, data analysed, and parameter settings.

The origin of *Ozotoceros bezoarticus* possibly dates back to 2.5 mya coinciding with a substantial cooling event; fossils are known from the late Pleistocene and Holocene of Brazil, the late Pleistocene of Uruguay, and the Holocene of Argentina (Gonzalez et al., 1998; Merino & Rossi, 2010).

##### Pudu

Both *Pudu* species are almost indistinguishable based on morphology, but do not evidently form a monophyletic group based on molecular data (Heckeberg et al., 2016; Gutiérrez et al., 2017; Zurano et al., 2019). *Pudu puda* was placed as the sister taxon to all Blastocerina in almost all of the analyses here and in previous studies with a sufficient taxon sampling. The systematic position of its congener, unfortunately, is much less certain. Here, *Pudu mephistophiles* was most often placed as the sister taxon to all Odocoileini plus *Rangifer* or to Odocoileini. Only in one topology *Pudu mephistophiles* was included within Blastocerina. In Zurano et al. (2019) it was the sister taxon to all other blastocerine taxa, while in Gutiérrez et al. (2017) it was placed in a polytomy with *Mazama nemorivaga* and all other blastocerine taxa.

The spatial and chronological origin of *Pudu* is unknown. *Pudu* most likely diverged from an odocoileine lineage, which existed in America since the Miocene-Pliocene-boundary (Merino & Rossi, 2010; Gonzalez et al., 2014). *Pudu* was probably restricted to South America since the Pliocene (Escamilo et al., 2010).

## Conclusion

The comprehensive data collection and results from the phylogenetic analyses provided new insights into the systematic relationships of fossil and extant cervids. These relationships were investigated using molecular and morphological characters separately and combined.

The morphological data sets were partly informative for extant taxa and gave new insights into the systematic relationships of fossil taxa. There were some consistent splits within the morphological topologies, for example the *Elaphurus*, Muntiacini, and Capreolini. The SFA and FPA approaches were particularly useful for investigating the placement of fossil taxa.

In most of the molecular and combined analyses, extant clades on subfamilial and tribal level were monophyletic. While systematic relationships within Cervinae were relatively stable, with many consistently recovered subclades, systematic relationships within Capreolinae were more variable. Even the monophyly of this subfamily could not be confirmed in all topologies.

No link between particularly incomplete taxa and phylogenetic instability was observed. For the Miocene cervids, a placement in a stem position between the outgroup and all other cervids, or in a sister position to Muntiacini was suggested in the analyses here. Most of the Miocene cervids were more closely related to each other than to other cervids. Plio- and Pleistocene cervids, were most often placed within or close to extant cervids and the majority of them within Cervini, some within Capreolinae. or Muntiacini.

I extensively tested the systematic positions of extant and especially fossil cervids for the first time under a comprehensive phylogenetic approach. Inclusion of more fossil cervids, postcranial characters, soft anatomy and life history data, and cytogenetics would be useful in future analyses. Further, rare genomic changes, such as gene duplication and genetic code changes, intron indels, and mitochondrial gene order changes, and SNP chips have become more popular as complementary markers and should be included as addition to the molecular partition in cervids.

## Supplemental Information

10.7717/peerj.8114/supp-1 Supplemental Information 1 List of extant and fossil specimens.

10.7717/peerj.8114/supp-2 Supplemental Information 2 Craniodental Measurements.

10.7717/peerj.8114/supp-3 Supplemental Information 3 GenBank Accession Numbers.

10.7717/peerj.8114/supp-4 Supplemental Information 4 Character Matrix.

10.7717/peerj.8114/supp-5 Supplemental Information 5 Dental Characters.

10.7717/peerj.8114/supp-6 Supplemental Information 6 Cranial Characters.

10.7717/peerj.8114/supp-7 Supplemental Information 7 Cranial and dental measuring distances. Drawings by Nicola Heckeberg.

10.7717/peerj.8114/supp-8 Supplemental Information 8 Parameters of Phylogenetic Analyses.

10.7717/peerj.8114/supp-9 Supplemental Information 9 Topologies.

## Additional Information and Declarations

### Data Availability

The following information was supplied regarding data availability: Molecular data are available at GenBank (Accession Numbers in Table S3). The character matrices, character and character state descriptions are available on Morphobank (https://morphobank.org/index.php/Projects/ProjectOverview/project_id/1021) and in Data Sets S1–S3. Specimen accession numbers and a list of specimens are available in Table S1. The specimens are deposited in the following collections: Museo Nacional de Ciencias Naturales Madrid, Spain (MNCN); National d’Histoire Naturelle Paris, France (MNHN); Natural History Museum London, UK (NHM/NHMUK PV); Naturhistorisches Museum Wien, Austria (NHMW); Naturhistorisches Museum Basel, Switzerland (NMB); National Museum Scotland Edinburgh, UK (NMS); Naturalis Leiden, The Netherlands (RGM); Staatliches Museum für Naturkunde Stuttgart, Germany (SMNS); Bayerische Staatssammlung für Paläontologie und Geologie, Munich, Germany (SNSB-BSPG); University Museum of Zoology Cambridge, UK (UMZC); Museum für Naturkunde Berlin, Germany (ZMB_MAM); Zoologisches Museum Hamburg, Germany (ZMH); Zoologische Staatssammlung Munich, Germany (ZSM).
