# Supplementary material for: The systematics of the Cervidae: a total evidence approach
Source: PeerJ. 2020 Feb 18;8:e8114. doi: 10.7717/peerj.8114 (PMC7034380; doi:10.7717/peerj.8114)
Supplement: Supplemental Information 7 — Drawings by Nicola Heckeberg. [file peerj-08-8114-s007.pdf]

Supplementary Information – Measurements

The systematics of the Cervidae: a total evidence approach

Nicola S. Heckeberg[1,2,3]

[1]Department of Earth and Environmental Sciences, Palaeontology & Geobiology, Ludwig-Maximilians-Universität München, Munich, Germany
[2]SNSB – Bayerische Staatssammlung für Paläontologie und Geologie, Munich, Germany
[3]Current address: Museum für Naturkunde, Leibniz Institute for Evolution and Biodiversity Science, Berlin, Germany

Table 1: List of measurements taken on the mandible including the dentition. Abbreviations correspond to those in Figures 1.

**Abbr.**	**Measuring distances - Mandibula**
pxL	Length of lower premolar
WAB	Total width of lower antepremolar tooth arch at base
WAT	Total width of lower antepremolar tooth arch at tips
TLL	Total length of lower premolars & molars
LLP	Length of lower premolars
LLM	Length of lower molars
LSY	Length of symphysis
TLM	Total length of mandibula
HRM	Height of ramus mandibulae
DCC	Distance: dorsal end of processus coronoideus - processus condylaris
LIM	Length of incisura mandibulae (parallel to tooth row at level of ventral rim of articulation surface of pr. condylaris)
LMD	Length of diastema
LCO	Length of processus coronoideus (at level of articular surface of pr. condylaris)

Table 2: List of measurements taken on the cranium including the dentition. Abbreviations correspond to those in Figures 2 and 3.

**Abbr.**	**Measuring distances - Cranium**
PxL	Length of upper premolar
LUP	Length of upper premolars
LUM	Length of upper molars
ICD	Distance between cristae on diastema on maxilla (smallest)
WMX	Width of maxilla at diastema
TLP	Total length of palate
TWP	Total width of palate between tooth rows
IPF	Distance between foramina palatina
TLS	Total length skull (ventral)
LBU	Length of bulla
WBU	Width of bulla
WBC	Width of braincase (widest)
NFM	Distance: crista nuchae - dorsal rim foramen magnum
NBO	Distance: crista nuchae - ventral end of basioccipital
OPX	Distance: anterior rim of orbita - anterior end of praemaxilla
LOR	Length of orbita
HOR	Height of orbita
LPV	Length of praeorbital vacuity
WPV	Width of praeorbital vacuity
LNA	Length of nasal (along midline)
TWS	Total width of skull (usually at zygomatic arch/orbita)
LLA	Length of lacrimal (facial portion)
HLA	Height of lacrimal (facial portion)
LPM	Medial length of pedicle
LPL	Lateral length of pedicle
PAP	Pedicle diameter anterior - posterior
PML	Pedicle diameter medial - lateral
LAN	Length of antler (main beam)

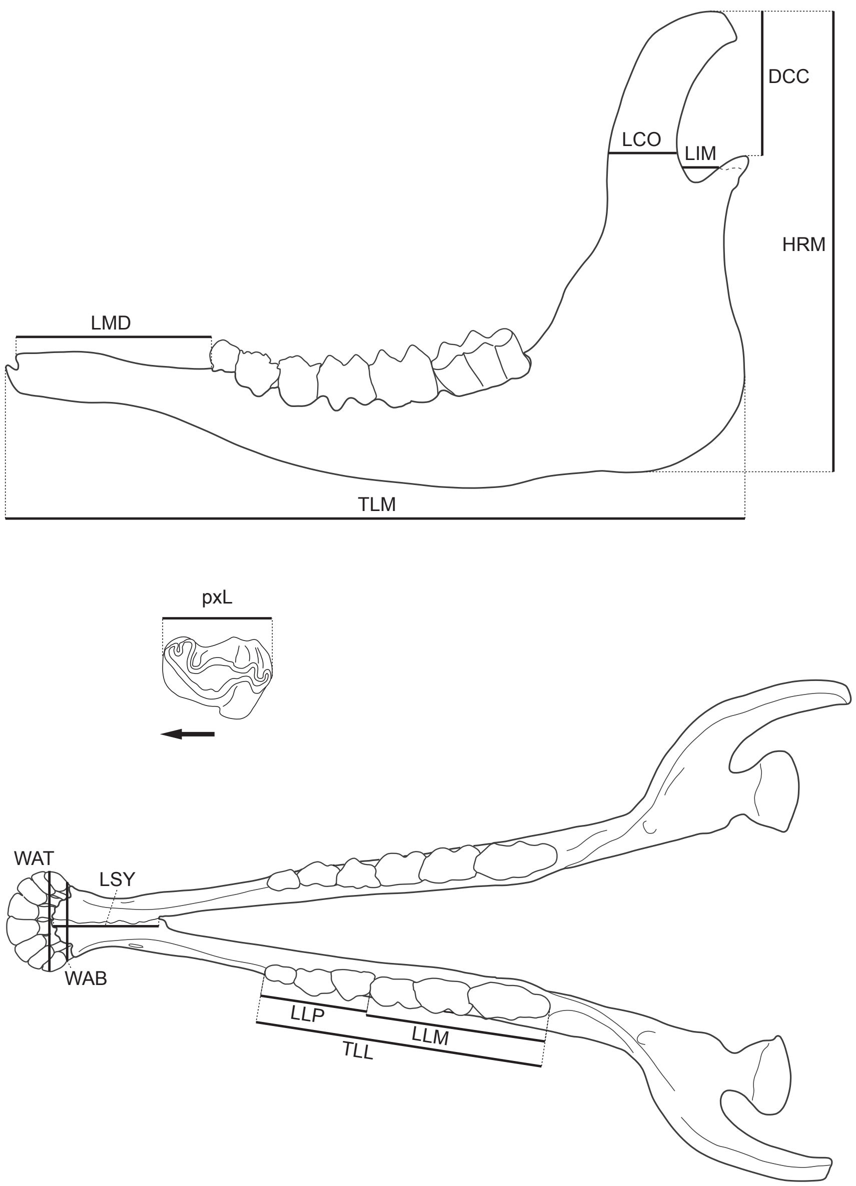

Figure 1: All measuring distances of the mandible in lateral and dorsal view and of the lower premolar. (Drawing by Nicola Heckeberg)

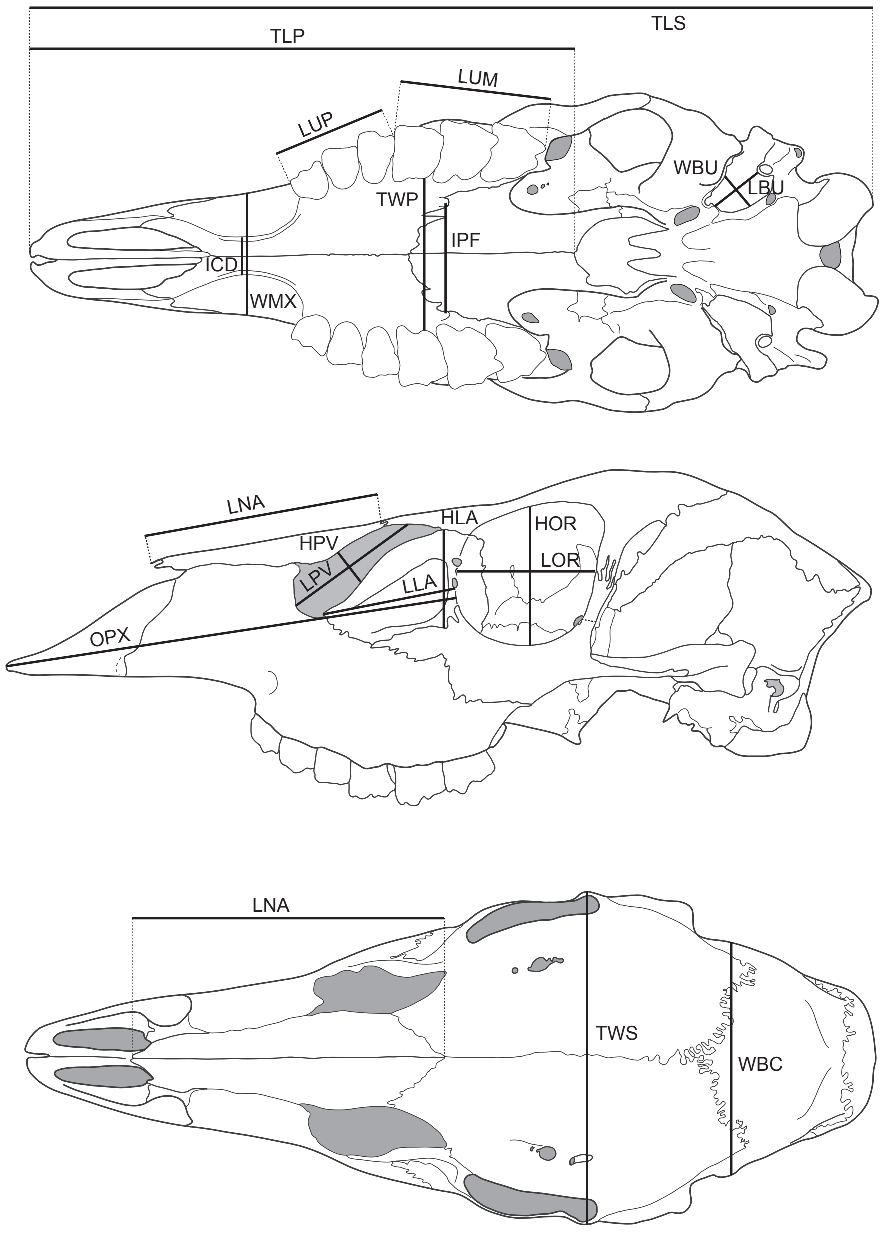

Figure 2: All measuring distances of the skull in ventral, lateral and dorsal view. (Drawing by Nicola Heckeberg)

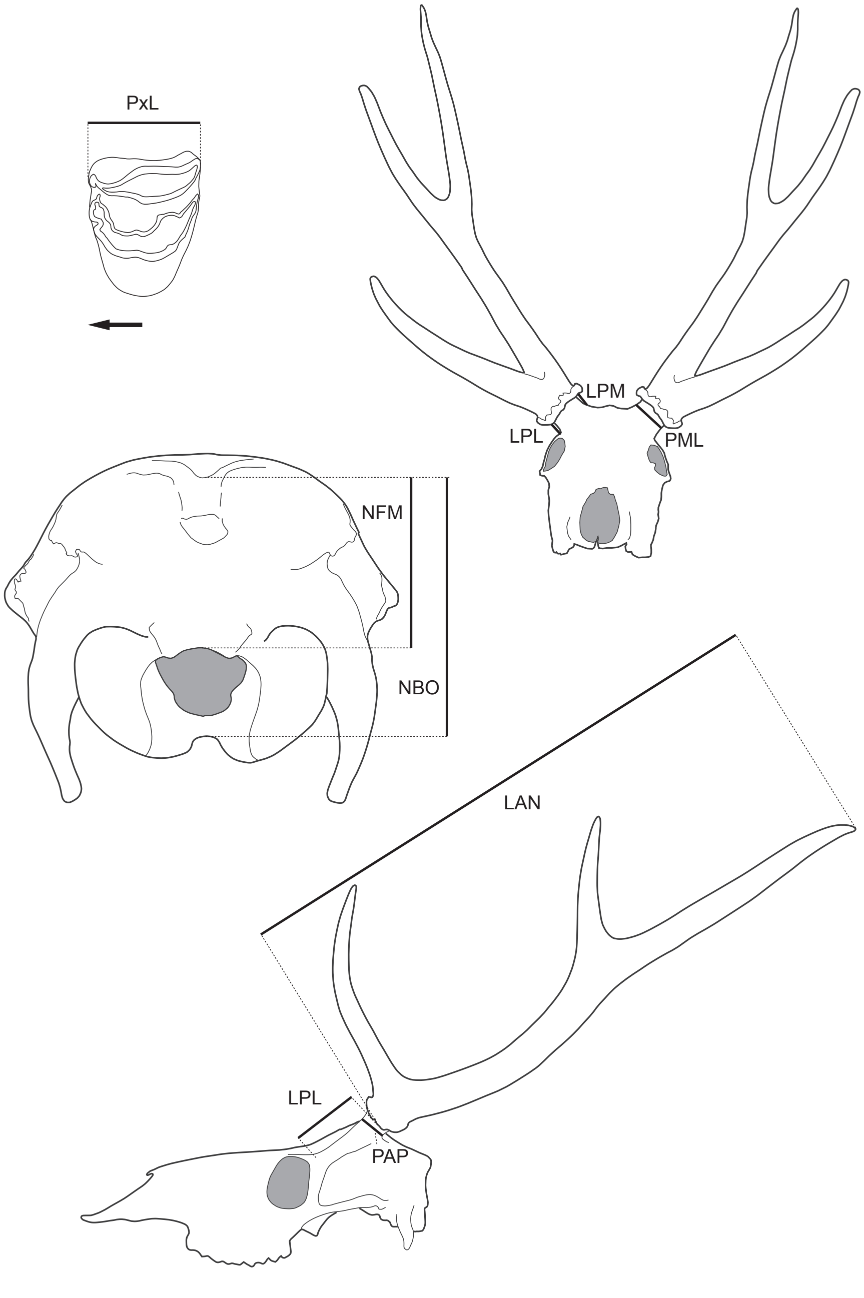

Figure 3: All measuring distances of the skull in occipital view, of the upper premolar, and of antlers and pedicles. (Drawing by Nicola Heckeberg)
