# Supplementary material for: The systematics of the Cervidae: a total evidence approach
Source: PeerJ. 2020 Feb 18;8:e8114. doi: 10.7717/peerj.8114 (PMC7034380; doi:10.7717/peerj.8114)
Supplement: Supplemental Information 8 [file peerj-08-8114-s008.pdf]

Supplementary Information – Parameters of Phylogenetic Analyses

The systematics of the Cervidae: a total evidence approach

Nicola S. Heckeberg[1,2,3]

[1]Department of Earth and Environmental Sciences, Palaeontology & Geobiology, Ludwig-Maximilians-Universität München, Munich, Germany
[2]SNSB – Bayerische Staatssammlung für Paläontologie und Geologie, Munich, Germany
[3]Current address: Museum für Naturkunde, Leibniz Institute for Evolution and Biodiversity Science, Berlin, Germany

Table 1: Overview of all analyses undertaken mainly based on the morphological character matrix. Dent=Dental, Cran=Cranial, UnO=unordered, O=ordered, N=No, Y=Yes; Opt. Crit.=Optimality Criterion, CI=consistency index, HI=homoplasy index, RI=retention index, RC=rescaled consistency index, E=Extant, F=Fossil.

**Analysis ID**	**Opt. Crit.**	**Data Set**	**Characters**	**Taxa**	**Ordered**	**CI**	**HI**	**RI**	**RC**	**Tree Length**
Dent_UnO_FE	MP	dental	79	78	N	0.2559	0.9006	0.4786	0.1225	1016
Dent_O_FE	MP	dental	79	78	Y	0.1848	0.9285	0.2116	0.0391	1412
Dent_O_E	MP	dental	79	47	Y	0.2567	0.8850	0.2982	0.0766	861
Dent_O_F	MP	dental	79	31	Y	0.3520	0.7458	0.4036	0.1420	358
Dent_MB_UnO	BI	dental	79	78	N	–	–	–	–	–
Dent_MB_O	BI	dental	79	78	Y	–	–	–	–	–
Dent_ML	ML	dental	79	78	N	–	–	–	–	–
Cran_UnO_FE	MP	cranial	89	78	N	–	–	–	–	–
Cran_O_FE	MP	cranial	89	78	Y	–	–	–	–	–
Cran_O_E	MP	cranial	89	47	Y	0.2186	0.8357	0.4329	0.0946	773
Cran_O_F	MP	cranial	89	31	Y	–	–	–	–	–
Cran_MB_UnO	BI	cranial	89	78	N	–	–	–	–	–
Cran_MB_O	BI	cranial	89	78	Y	–	–	–	–	–
Cran_ML	ML	cranial	89	78	N	–	–	–	–	–
Combi_UnO_FE	MP	combined	168	78	N	0.2216	0.8825	0.4312	0.0956	2017
Combi_O_FE	MP	combined	168	78	Y	0.1597	0.9155	0.1653	0.0264	2806
Combi_O_E	MP	combined	168	47	Y	0.2583	0.8503	0.4335	0.1120	1510
Combi_O_F	MP	combined	168	31	Y	0.3238	0.7350	0.1556	0.0504	698
Combi_MB_UnO	BI	combined	168	78	N	–	–	–	–	–
Combi_MB_O	BI	combined	168	78	Y	–	–	–	–	–
Combi_ML	ML	combined	168	78	N	–	–	–	–	–

Table 2: Results from the Stepping Stone Sampling analyses. Values represent mean Bayes Factors calculated from the mean of the sum of the marginal likelihoods of the two runs of each analysis.

	Unpart Unord Gam	PartMin	PartMax	UnPart NoGam	UnPartOrd
UnpartUnordGam	—	-18,792119	-22,391951	-69,252349	4,000185
PartMin	18,792119	—	-3,599831	—	—
PartMax	22,391951	3,599831	—	—	—
UnPartNoGam	69,252349	—	—	—	—
UnPartOrd	-4,000185	—	—	—	—

Table 3: Statistical data for all molecular markers

Gene	Abbr.	Total Length	Excluded	Final Length	Coding	#	ingroup	outgroup	Identical sites*	Pairwise Identity	Ungapped lengths†	GC	A	C	G	T	Other	Gaps [-]
kappa-casein	*Csn*	714	1-349	369	Y	20	13	7	263 (71.3%)	93.4%	312.0 ±90.4; 156; 369	2,864 (45.9%)	1,908 (30.6%)	1,837 (29.4%)	1,027 (16.5%)	1,466 (23.5%)	Y:1	0 (0.0%)
alpha-lactalbumin	*Lalba*	525	1-16, 227-235, 389-404, 505-525	465	N	25	19	6	282 (60.6%)	93.1%	442.6 ±38.5; 269; 464	4,528 (40.2%)	2,813 (25.4%)	2,271 (20.5%)	2,257 (20.4%)	3,722 (33.6%)	Y:1	195 (1.7%)
protein kinase C iota	*Prkci*	578	1-28, 169-183, 292-306, 570-578	513	N	29	23	6	348 (67.8%)	95.8%	498.2 ±19.6; 415; 509	4,014 (27.4%)	4,829 (33.4%)	1,677 (11.6%)	2,337 (16.2%)	5,596 (38.7%)	Y:4; W:3; R:2	189 (1.3%)
prion protein	*Prnp*	863	1-37, 254-277, 351-353,831-863	768	Y	21	15	6	631 (82.2%)	97.2%	742.3 ±97.3; 315; 768	8,556 (54.9%)	3,939 (25.3%)	3,868 (24.8%)	4,688 (30.1%)	3,073 (19.7%)	Y:11; W:2; S:1; R:6; M:1	0 (0.0%)
sex determining region on y-chromosome	*Sry*	690	–	690	Y	70	66	4	510 (73.9%)	97.2%	629.6 ±90.7; 146; 690	20,849 (47.3%)	14,032 (31.8%)	10,677 (24.2%)	10,172 (23.1%)	9,193 (20.9%)	–	18 (0.0%)
mitochondrial genome	mtG	14904	–	14904	Y	46	39	7	8,748 (58.8%)	88.9%	14868.6 ±2.2; 14861; 14878	259,297 (37.8%)	226,307 (33.1%)	167,043 (24.4%)	92,254 (13.5%)	198,332 (29.0%)	Y:9; S:2; R:5; N:1; M:2; K:2	1,627 (0.2%)
cytochrome b	*Cytb*	1140	–	1140	Y	130	124	6	583 (51.1%)	87.9%	na	52,573 (35.5%)	41,340 (27.9%)	35,444 (23.9%)	17,129 (11.6%)	39,088 (26.4%)	Y:52; W:15; S:3; R:22; M:5; K:2; D:1	15,099 10.2%

Notes. *positions refer to final length sequence; †values are provided as [mean ±Stdv; min; max]; columns 'GC' to 'T' and 'Gaps': Frequemcy (%) of non-gaps
