# Supplementary material for: The systematics of the Cervidae: a total evidence approach
Source: PeerJ. 2020 Feb 18;8:e8114. doi: 10.7717/peerj.8114 (PMC7034380; doi:10.7717/peerj.8114)
Supplement: Supplemental Information 9 [file peerj-08-8114-s009.pdf]

Supplementary Information – Topologies

The systematics of the Cervidae: a total evidence approach

Nicola S. Heckeberg[1,2,3]

[1]Department of Earth and Environmental Sciences, Palaeontology & Geobiology, Ludwig-Maximilians-Universität München, Munich, Germany
[2]SNSB – Bayerische Staatssammlung für Paläontologie und Geologie, Munich, Germany
[3]Current address: Museum für Naturkunde, Leibniz Institute for Evolution and Biodiversity Science, Berlin, Germany

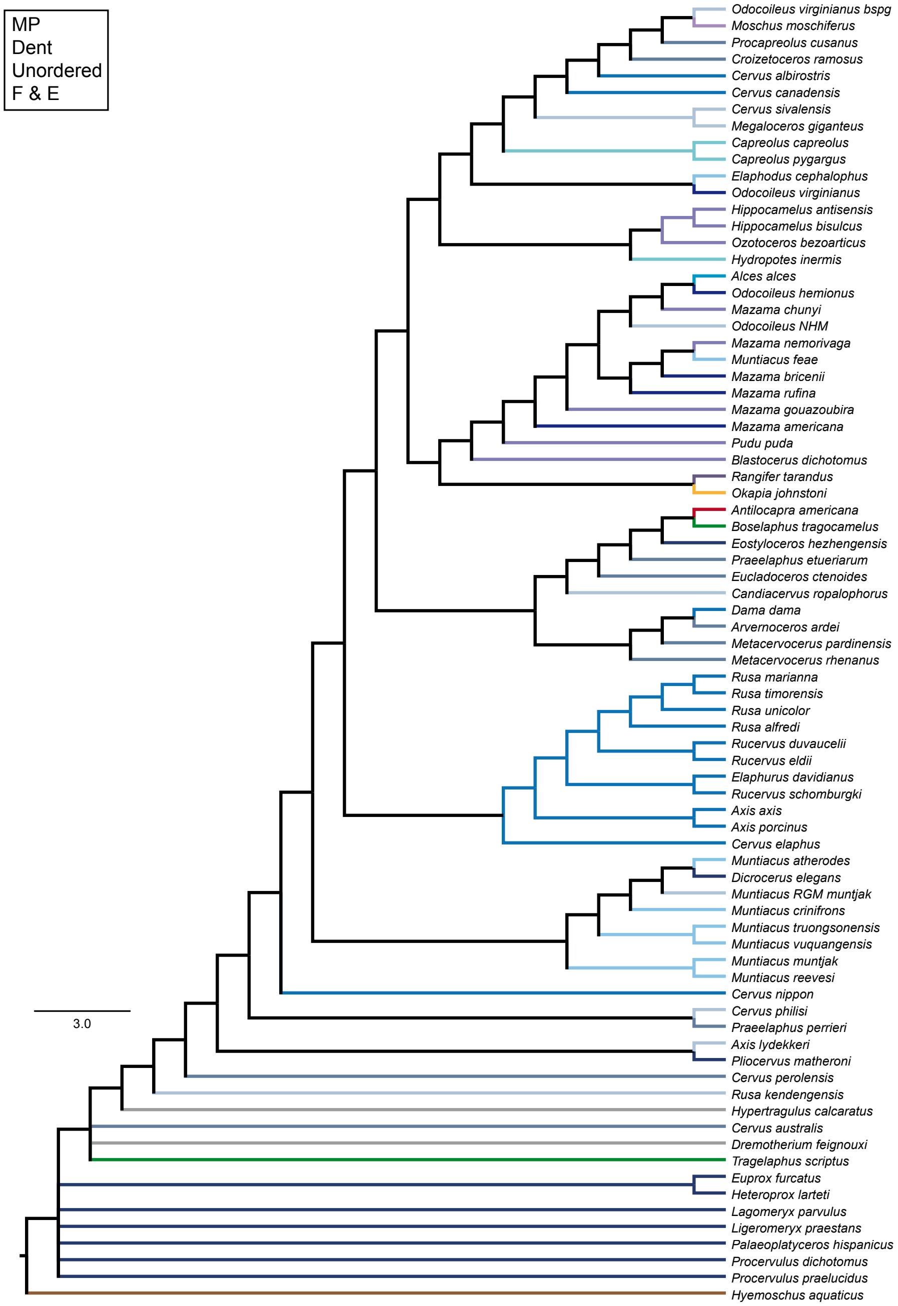

Figure 1: Consensus topology of the MP analysis based on the unordered dental character set for fossil and extant taxa.

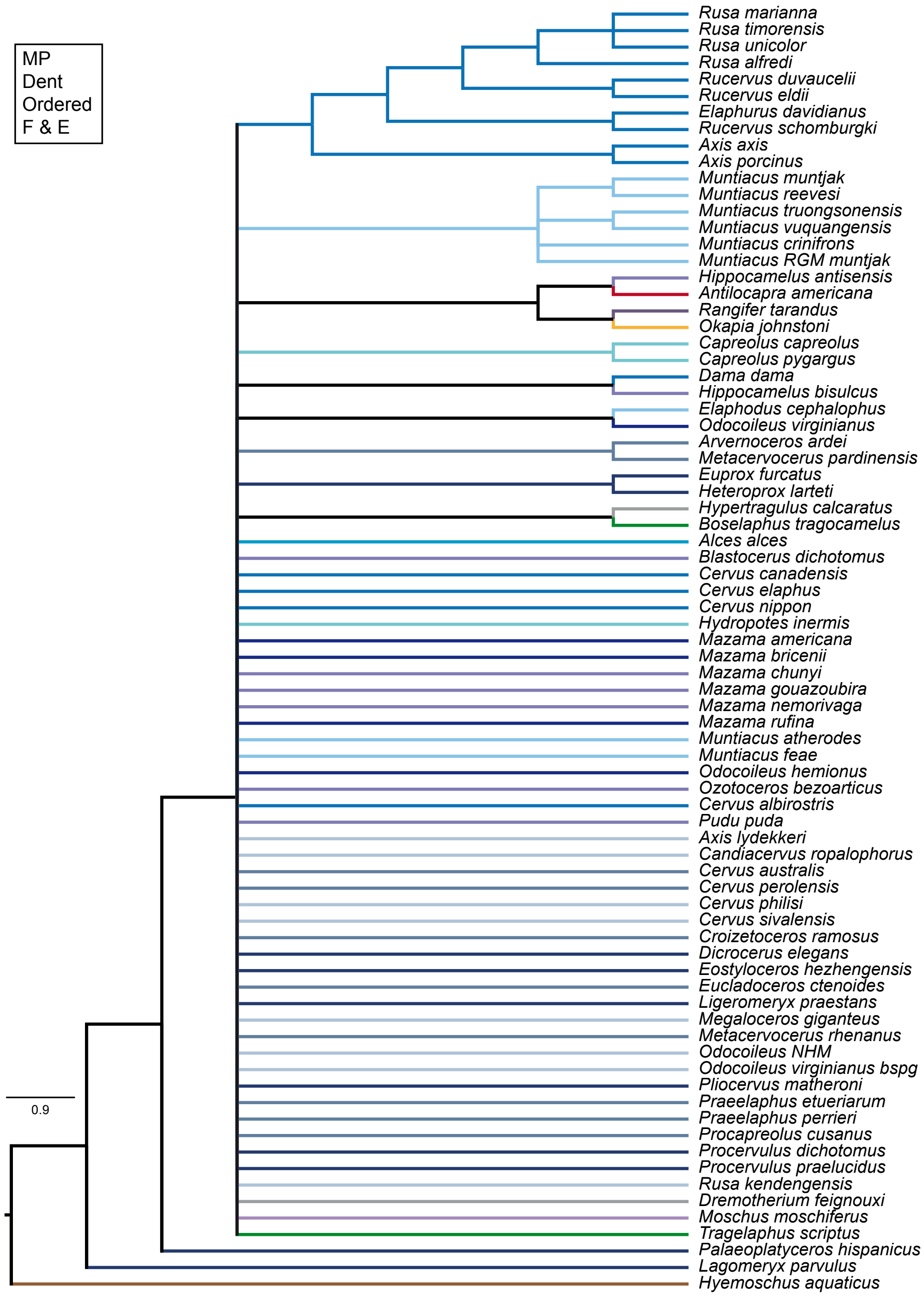

Figure 2: Consensus topology of the MP analysis based on the ordered dental character set for fossil and extant taxa.

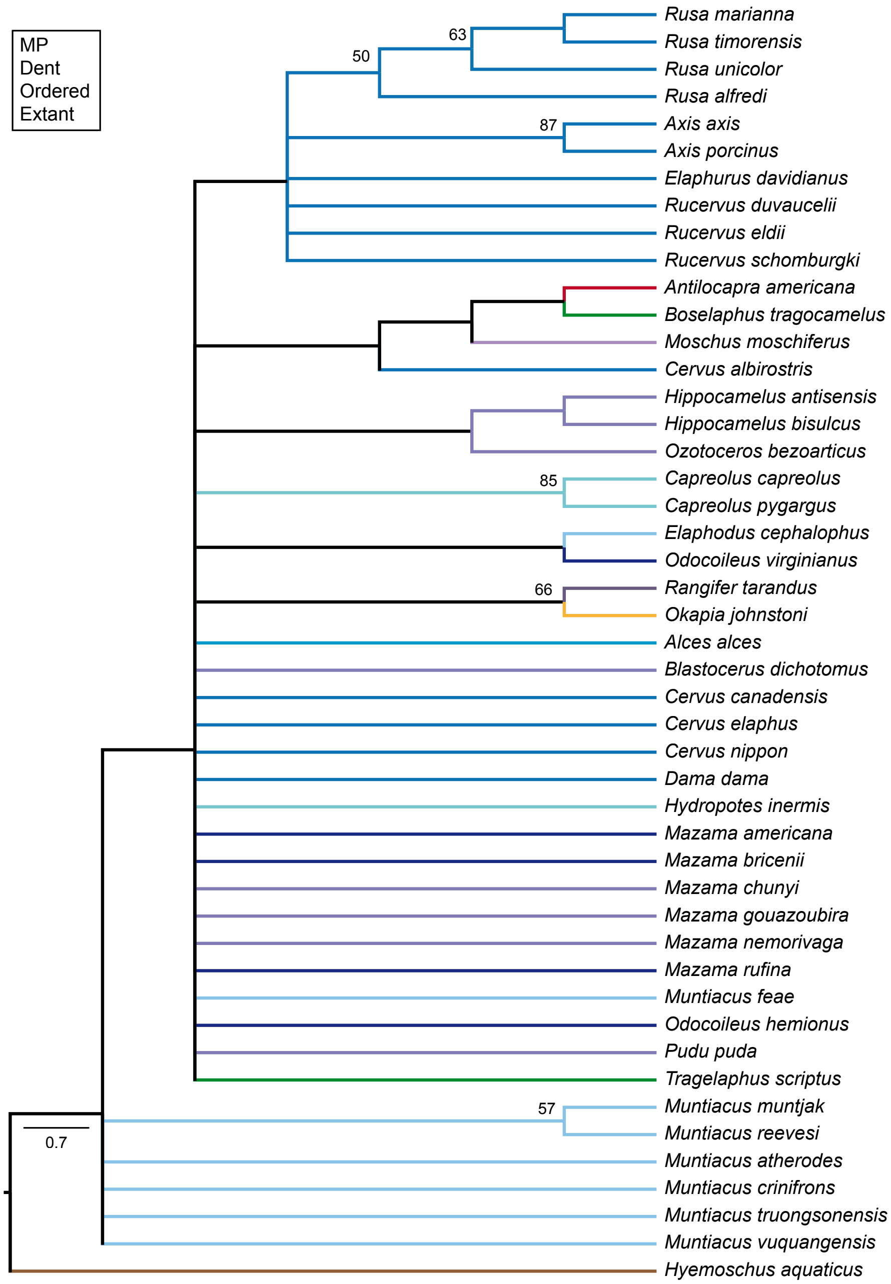

Figure 3: Consensus topology of the MP analysis based on the ordered dental character set for extant taxa.

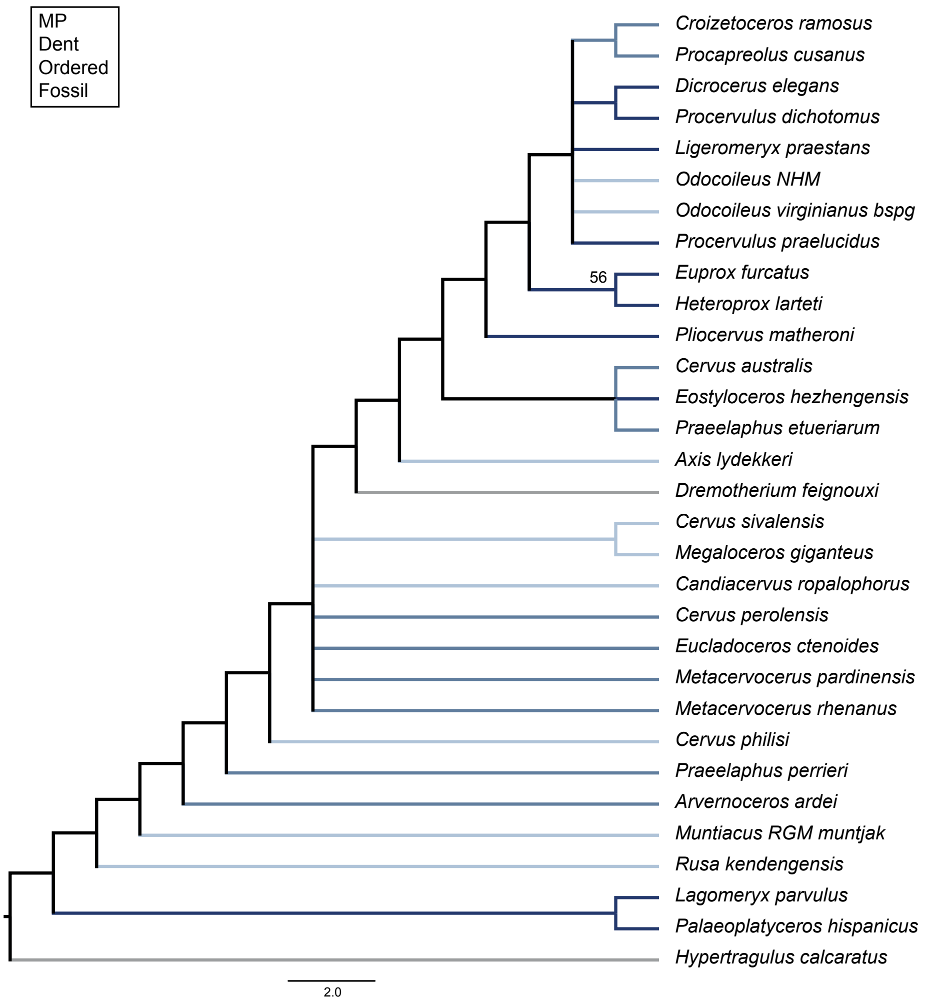

Figure 4: Consensus topology of the MP analysis based on the ordered dental character set for fossil taxa.

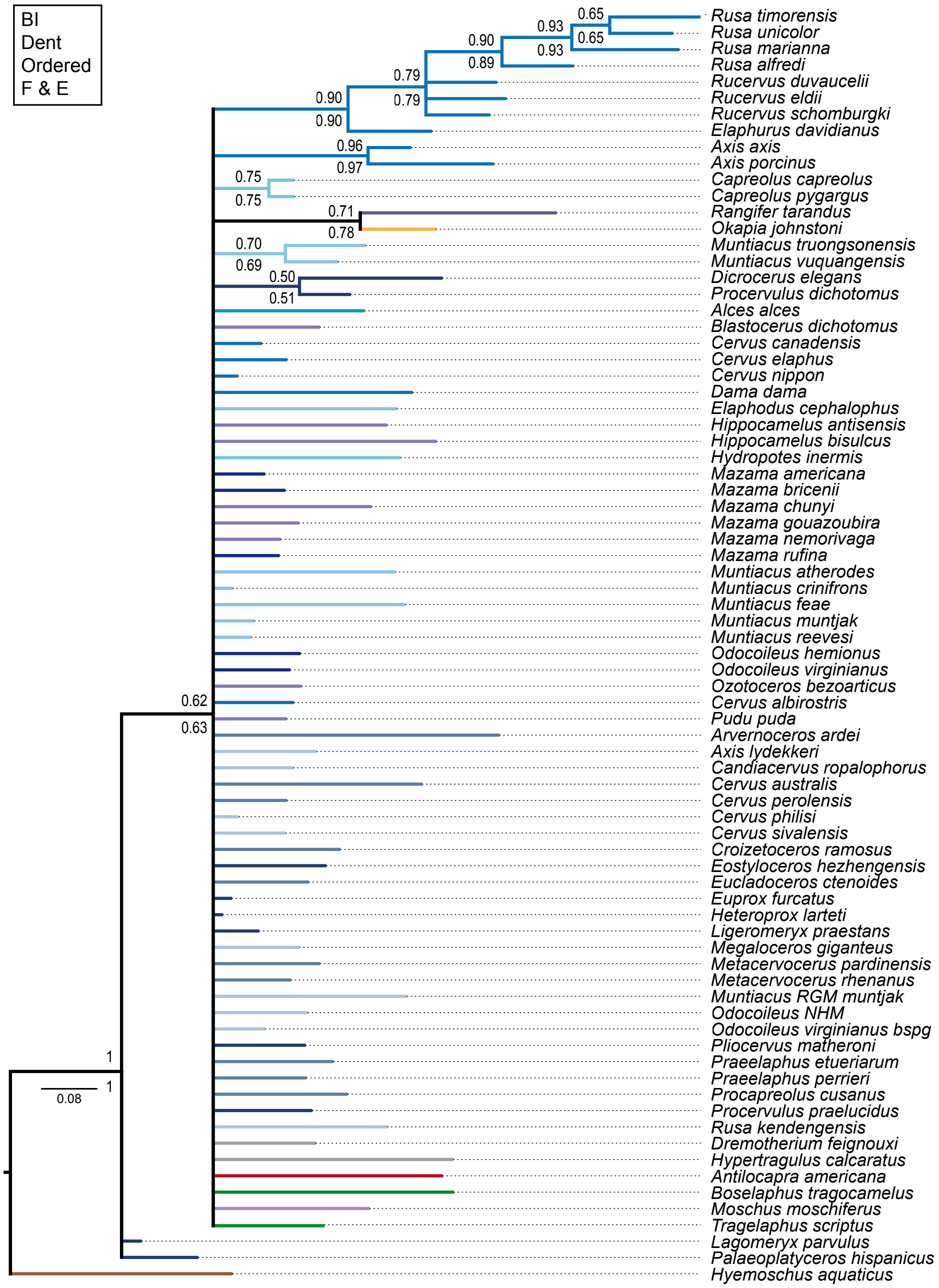

Figure 5: Consensus topology of the BI analysis based on the ordered and unordered dental character set for fossil and extant taxa. Posterior probabilities of the ordered analysis are above the branches, the posterior probabilities of the unordered analysis are below the branches.

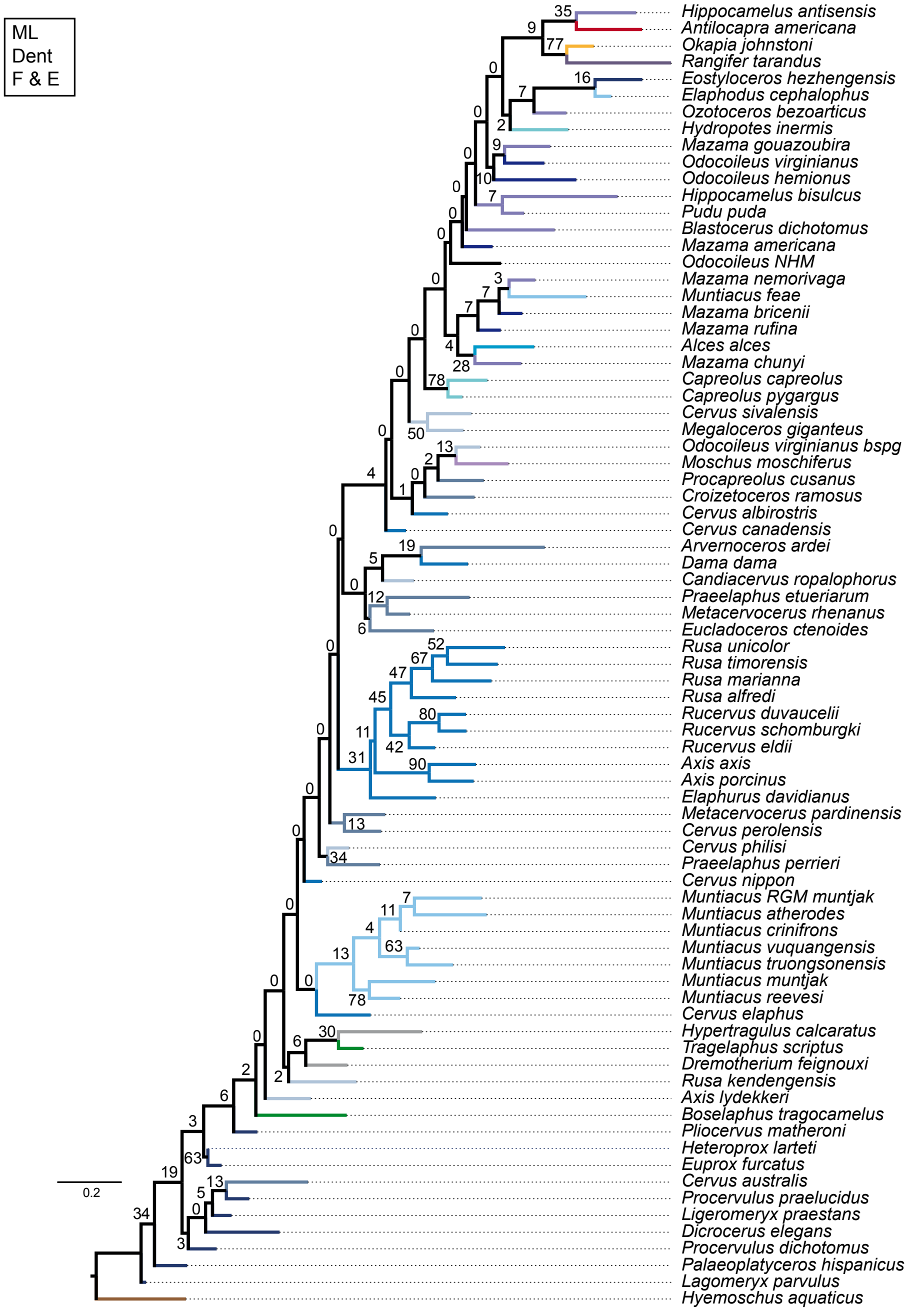

Figure 6: Best tree of the ML analysis based on the dental character set for fossil and extant taxa. Bootstrap support values are shown.

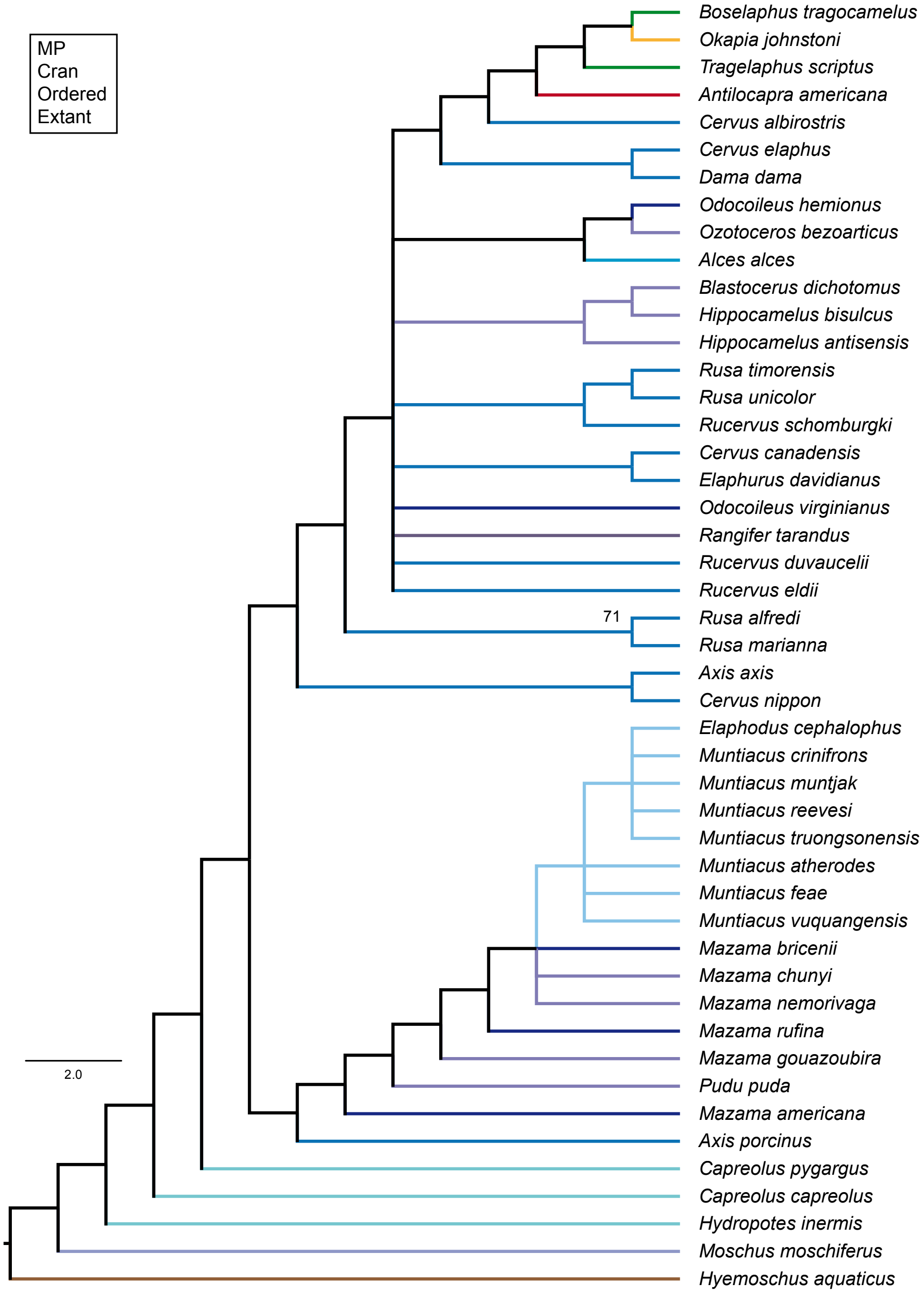

Figure 7: Consensus topology of the MP analysis based on the ordered cranial character set for extant taxa.

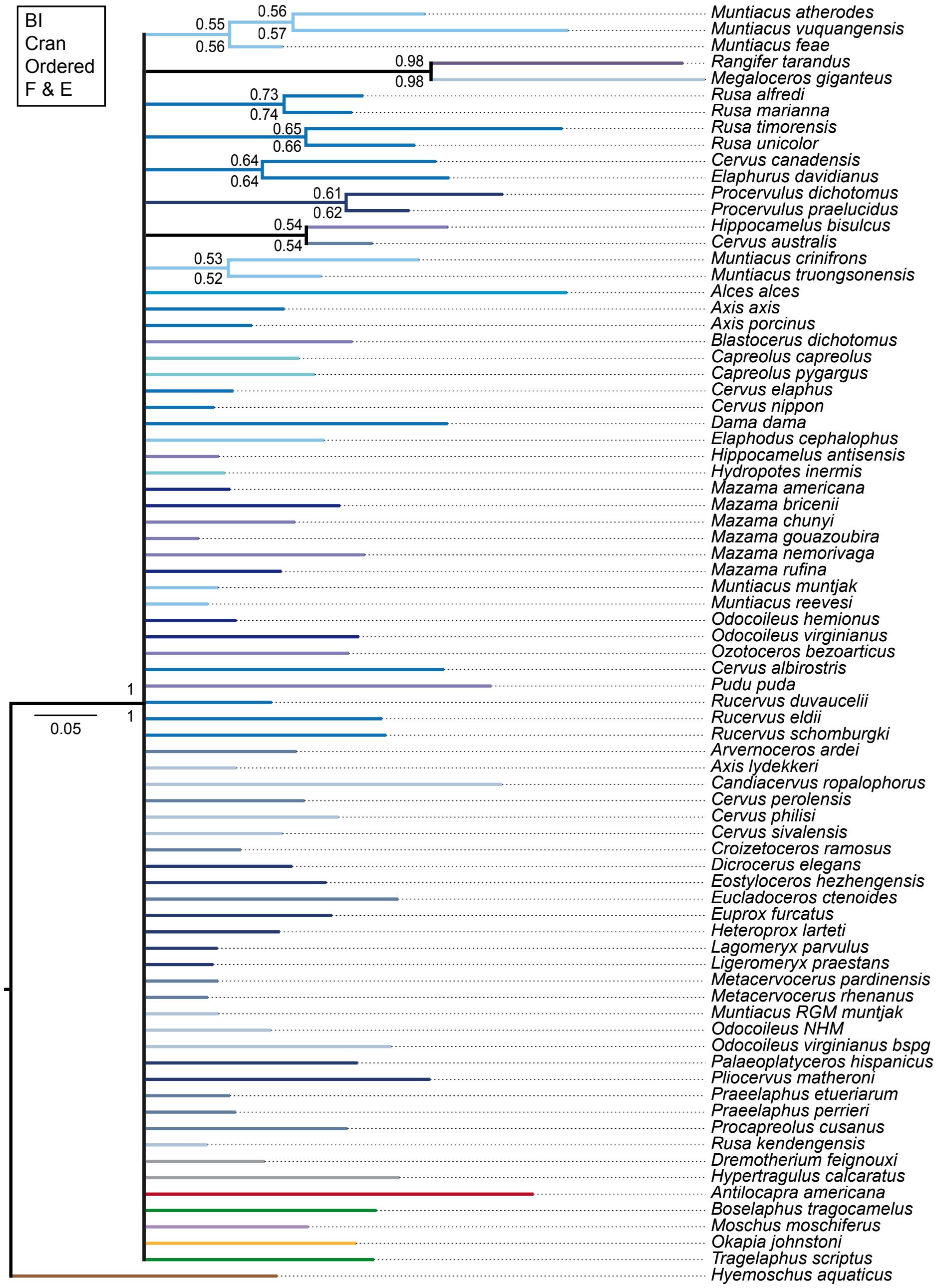

Figure 8: Consensus topology of the BI analysis based on the ordered and unordered cranial character set for fossil and extant taxa. Posterior probabilities of the ordered analysis are above the branches, posterior probabilities of the unordered analysis are below the branches.

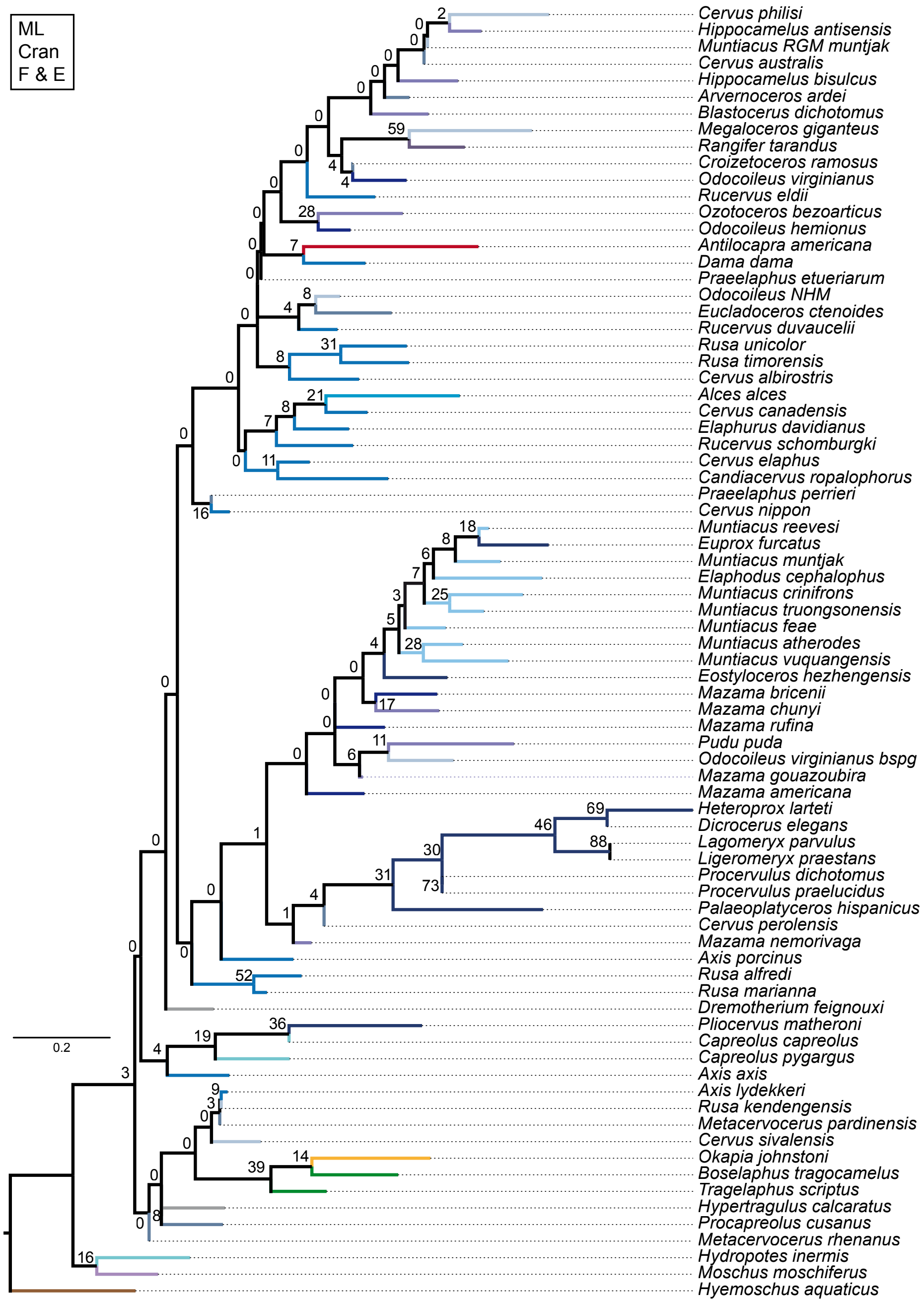

Figure 9: Best tree of the ML analysis based on the cranial character set for fossil and extant taxa. Bootstrap support values are shown.

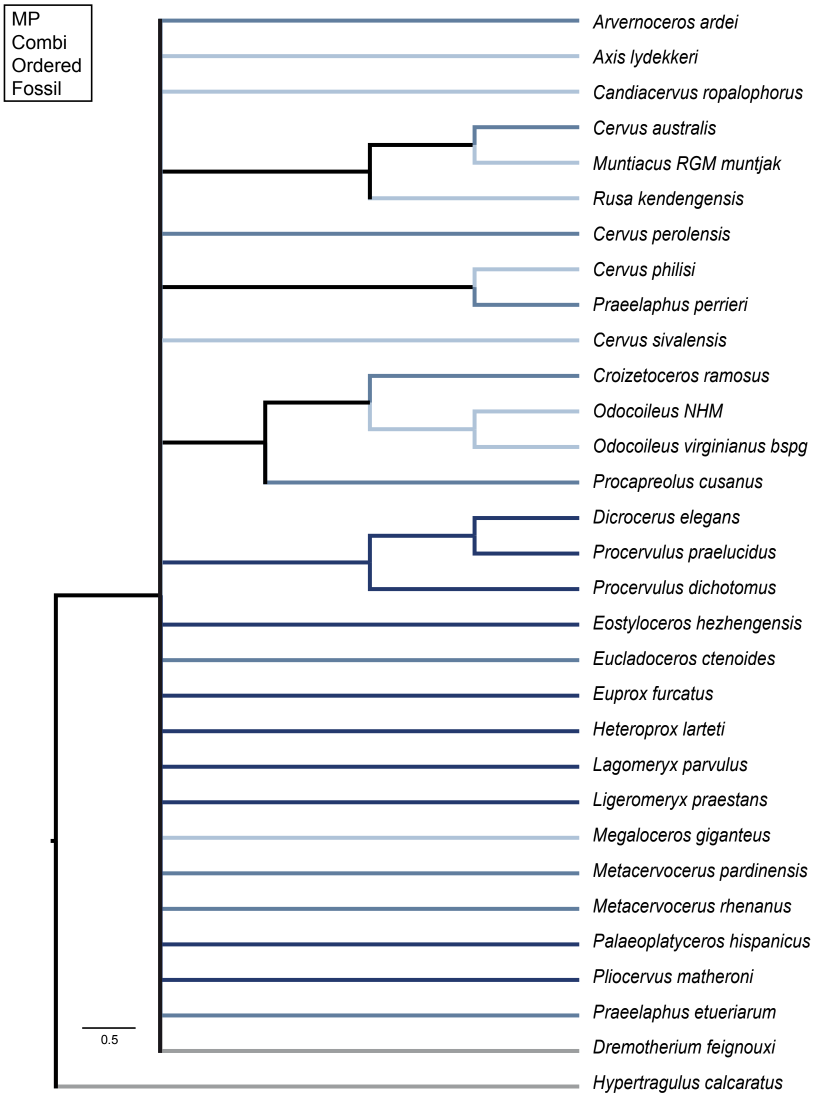

Figure 10: Consensus topology of the MP analysis based on the ordered combined character set for fossil taxa.

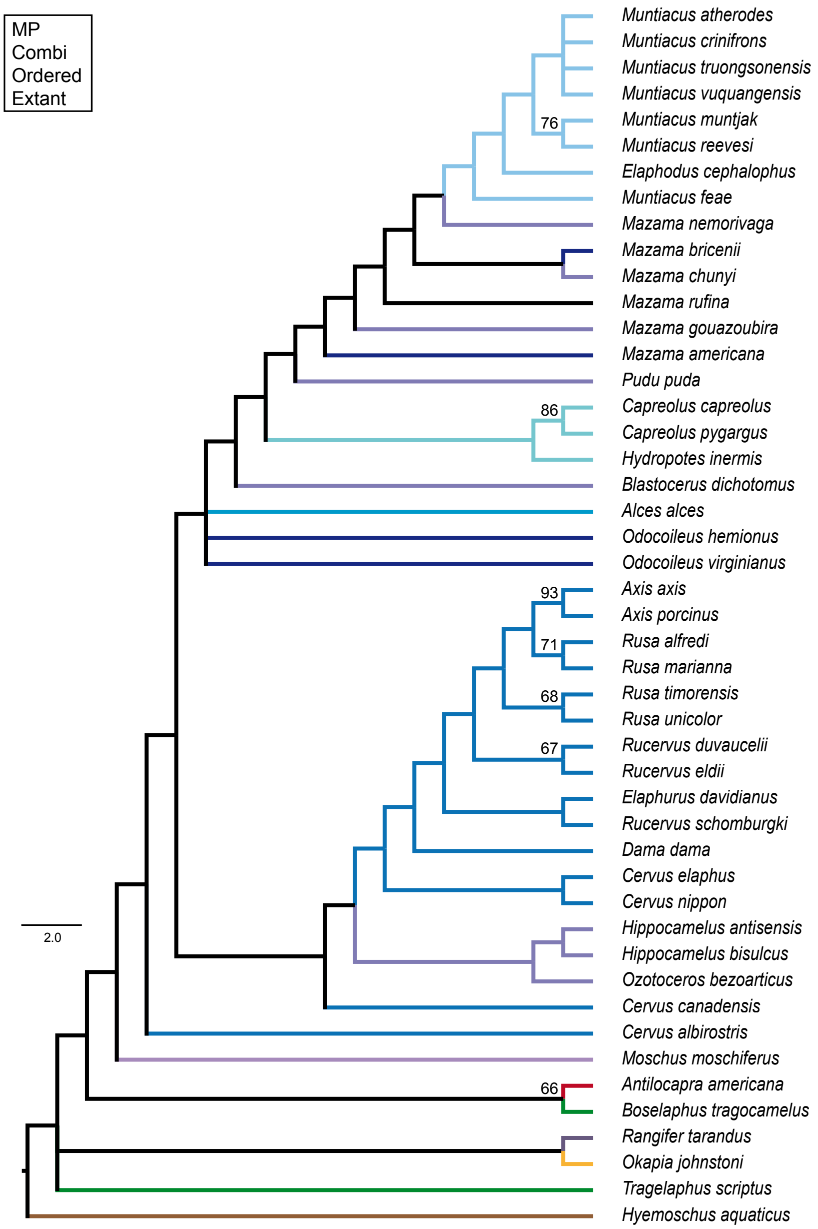

Figure 11: Consensus topology of the MP analysis based on the ordered combined character set for extant taxa.

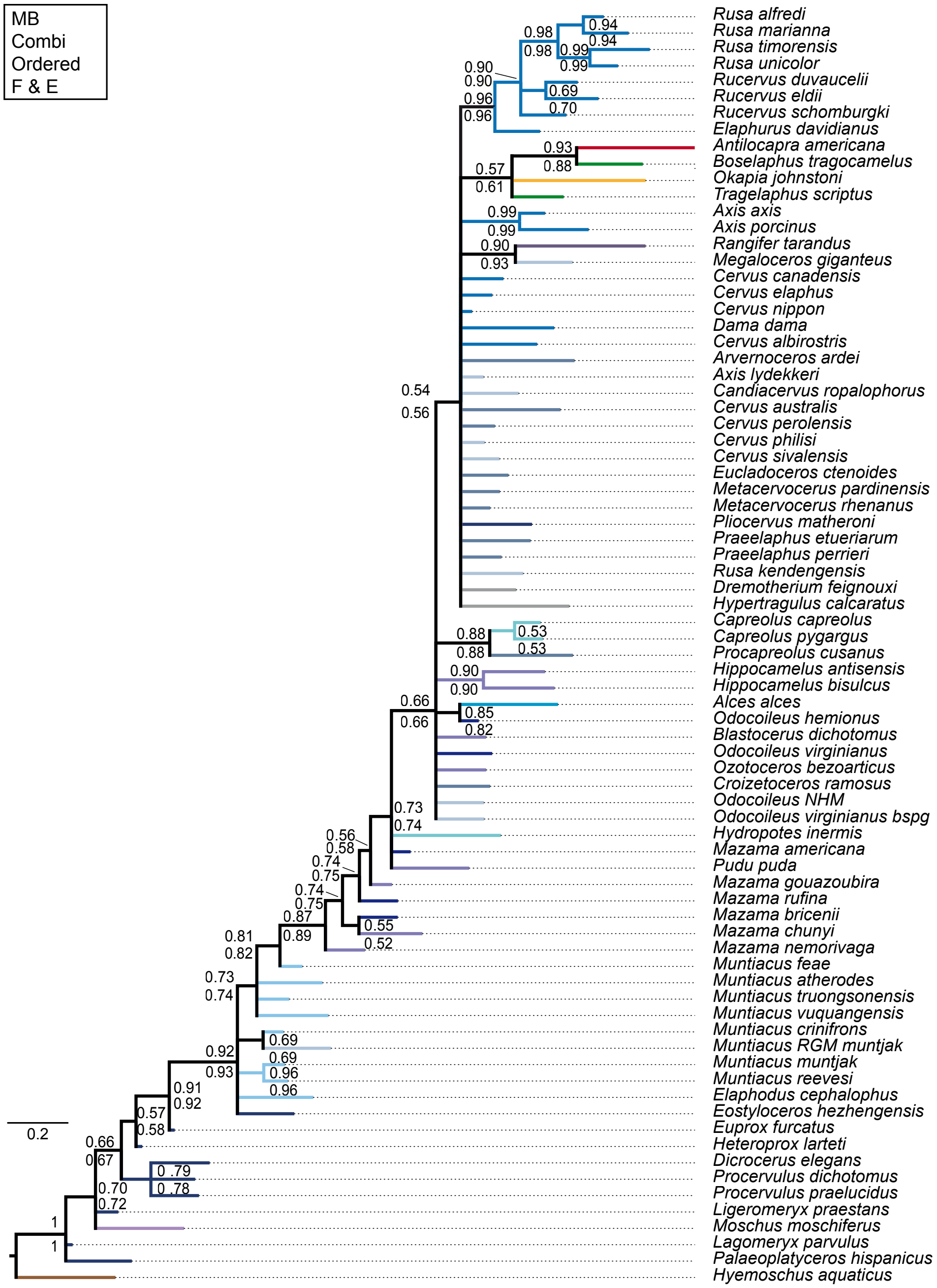

Figure 12: Consensus topology of the BI analysis based on the ordered and unordered combined character set for fossil and extant taxa. Posterior probabilities of the ordered analysis are above the branches, posterior probabilities of the unordered analysis are below the branches.

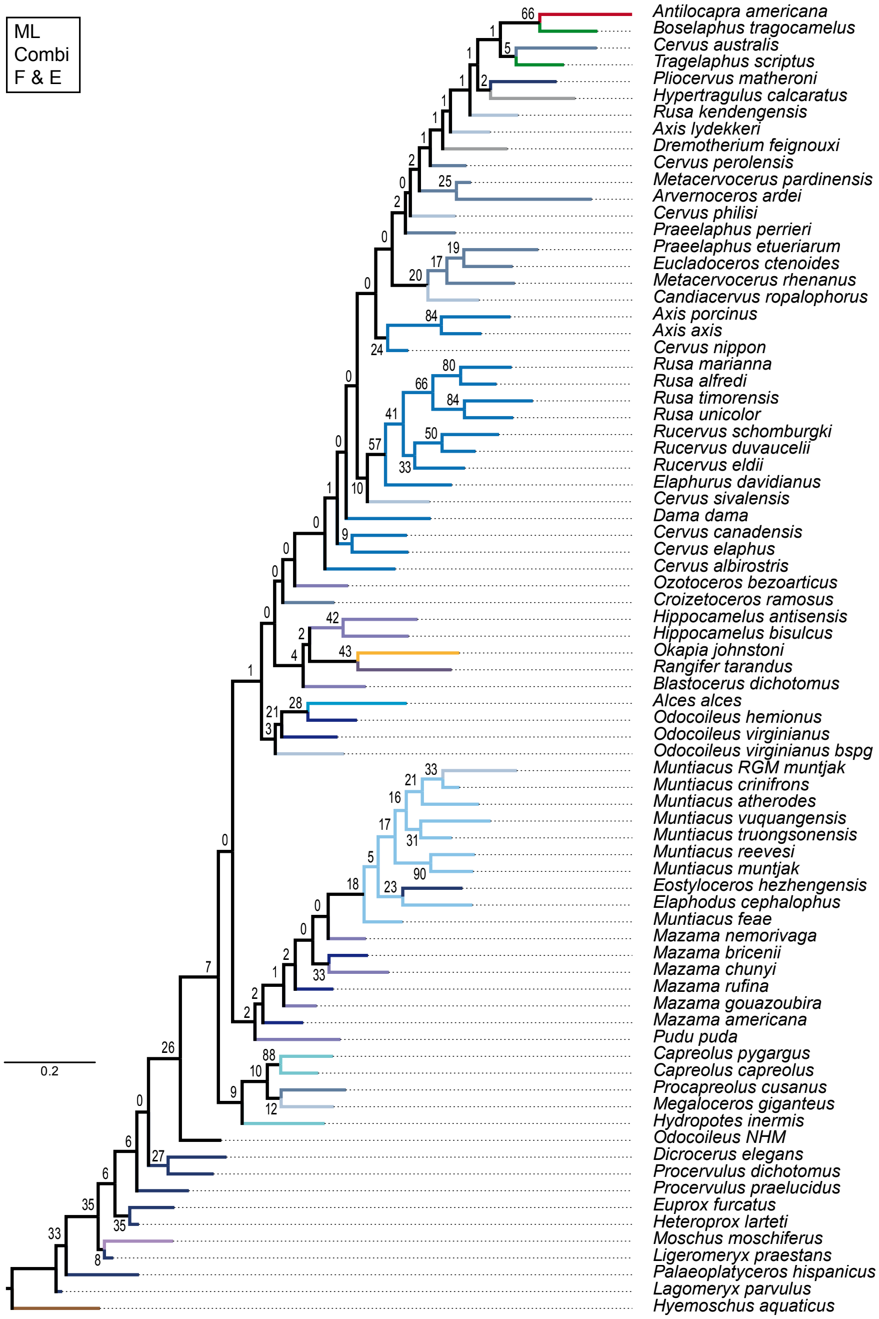

Figure 13: Best tree of the ML analysis based on the combined character set for fossil and extant taxa. Bootstrap support values are shown.

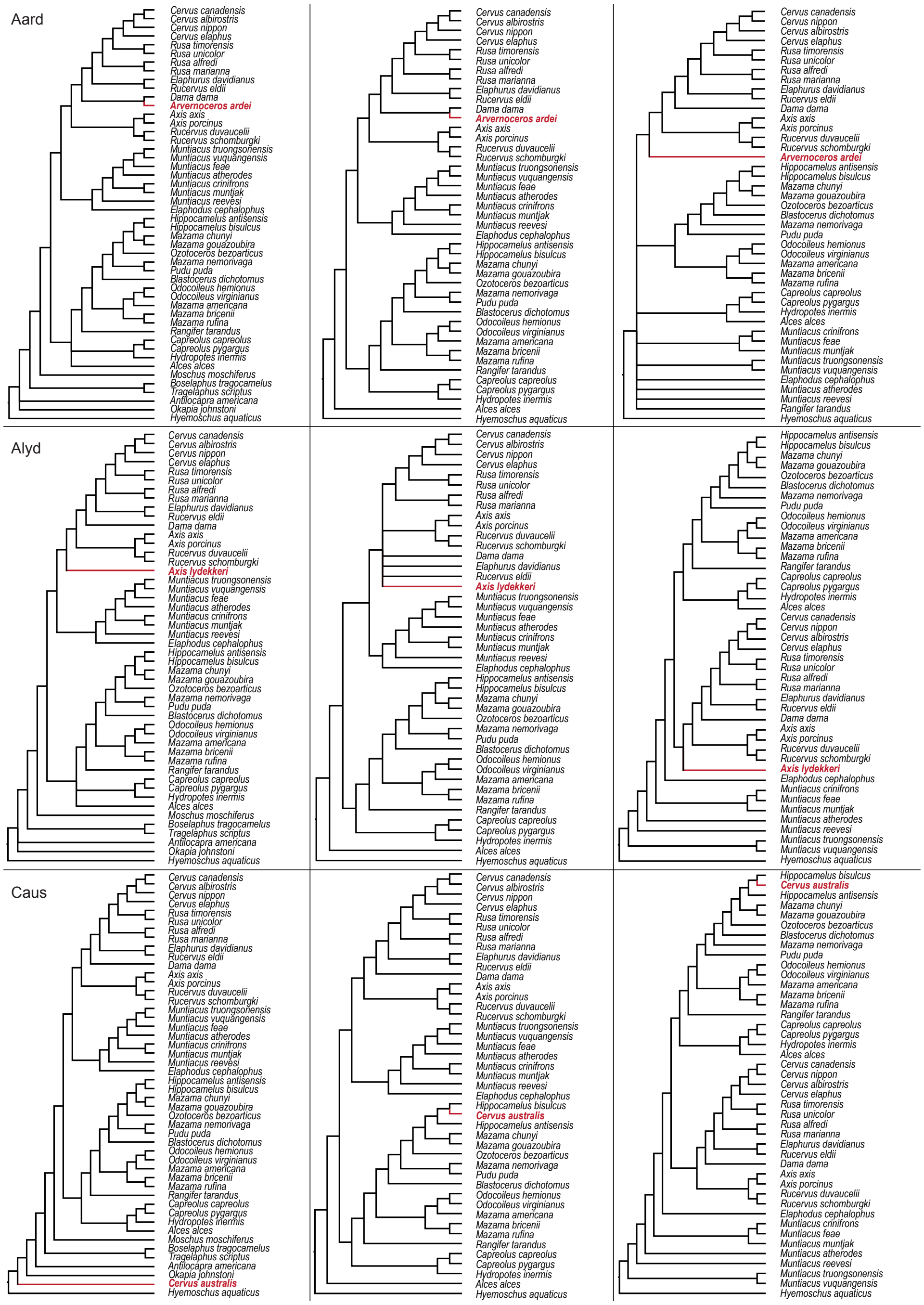

Figure 14: Overview of the three single fossil analyses for *Arvernoceros ardei*, *Axis lydekkeri*, and *Cervus australis*. Left: trees based on the supermatrix of the mitochondrial genome and the combined morphological data including outgroup taxa, middle: supermatrix excluding most outgroup taxa, right: backbone analyses.

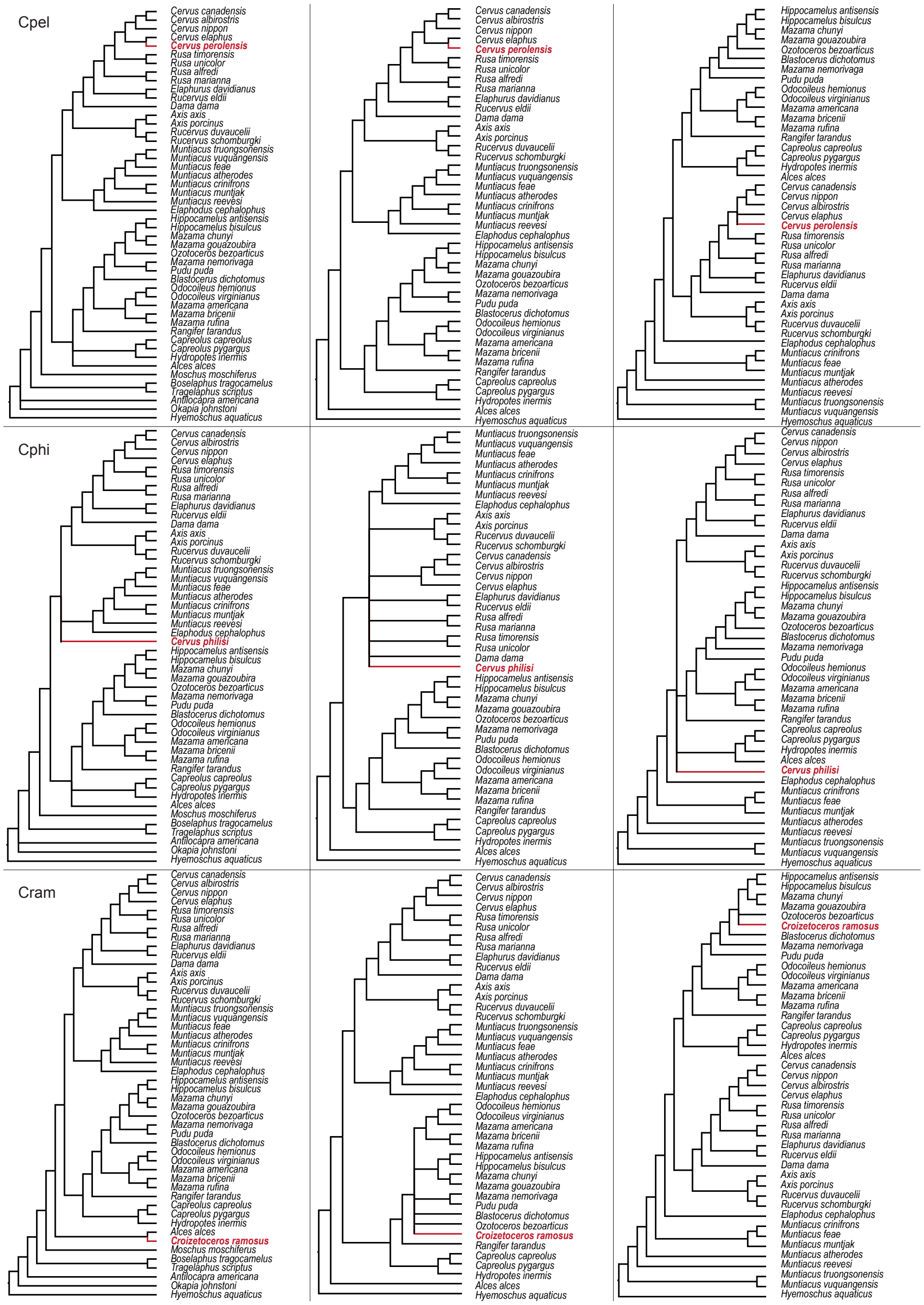

Figure 15: Overview of the three single fossil analyses for *‘Cervus’ perolensis*, *‘Cervus’ philisi*, and *Croizetoceros ramosus*. Left: trees based on the supermatrix of the mitochondrial genome and the combined morphological data including outgroup taxa, middle: supermatrix excluding most outgroup taxa, right: backbone analyses.

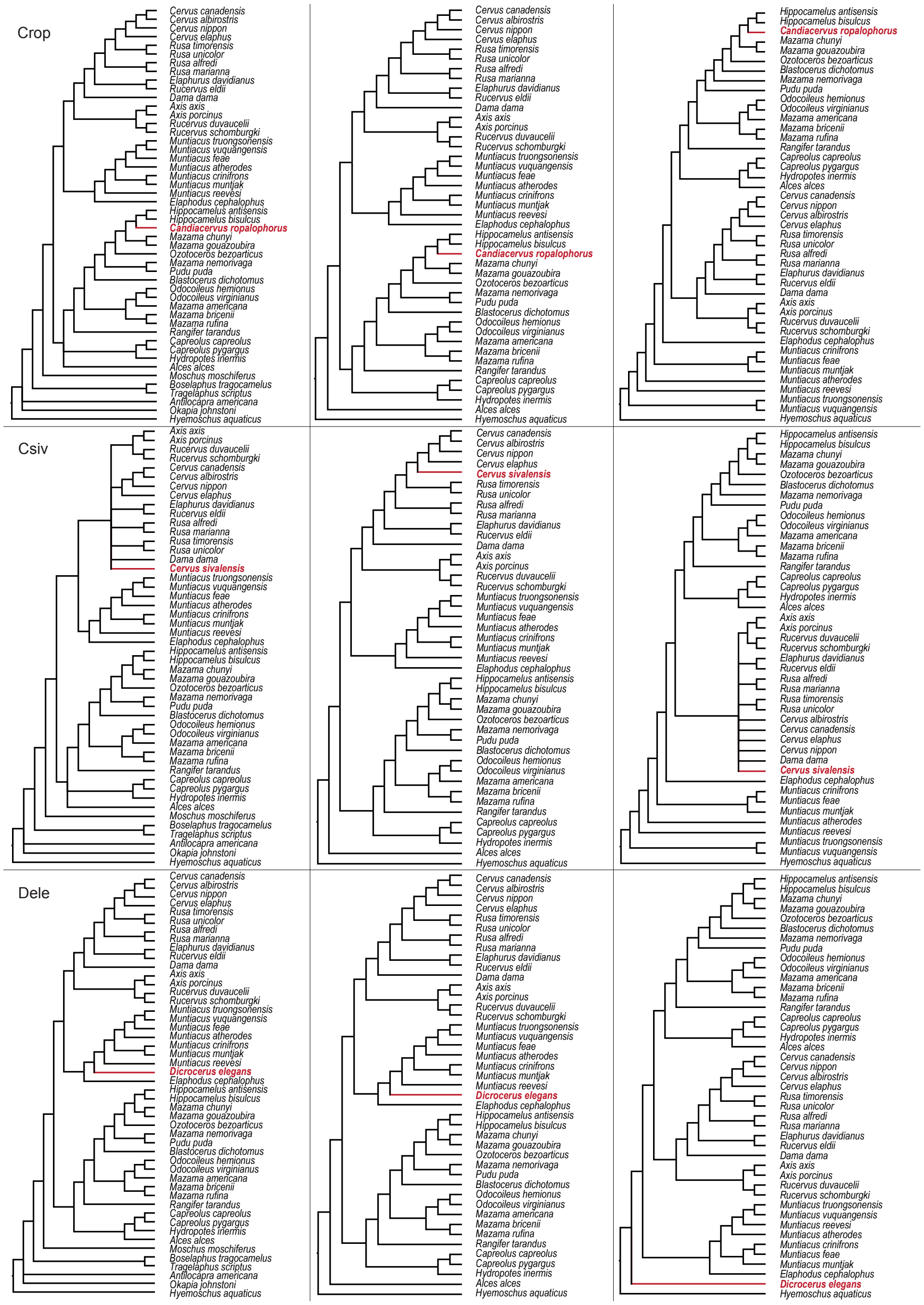

Figure 16: Overview of the three single fossil analyses for *Candiacervus ropalophorus*, *'Cervus' sivalensis*, and *Dicrocerus elegans*. Left: trees based on the supermatrix of the mitochondrial genome and the combined morphological data including outgroup taxa, middle: supermatrix excluding most outgroup taxa, right: backbone analyses.

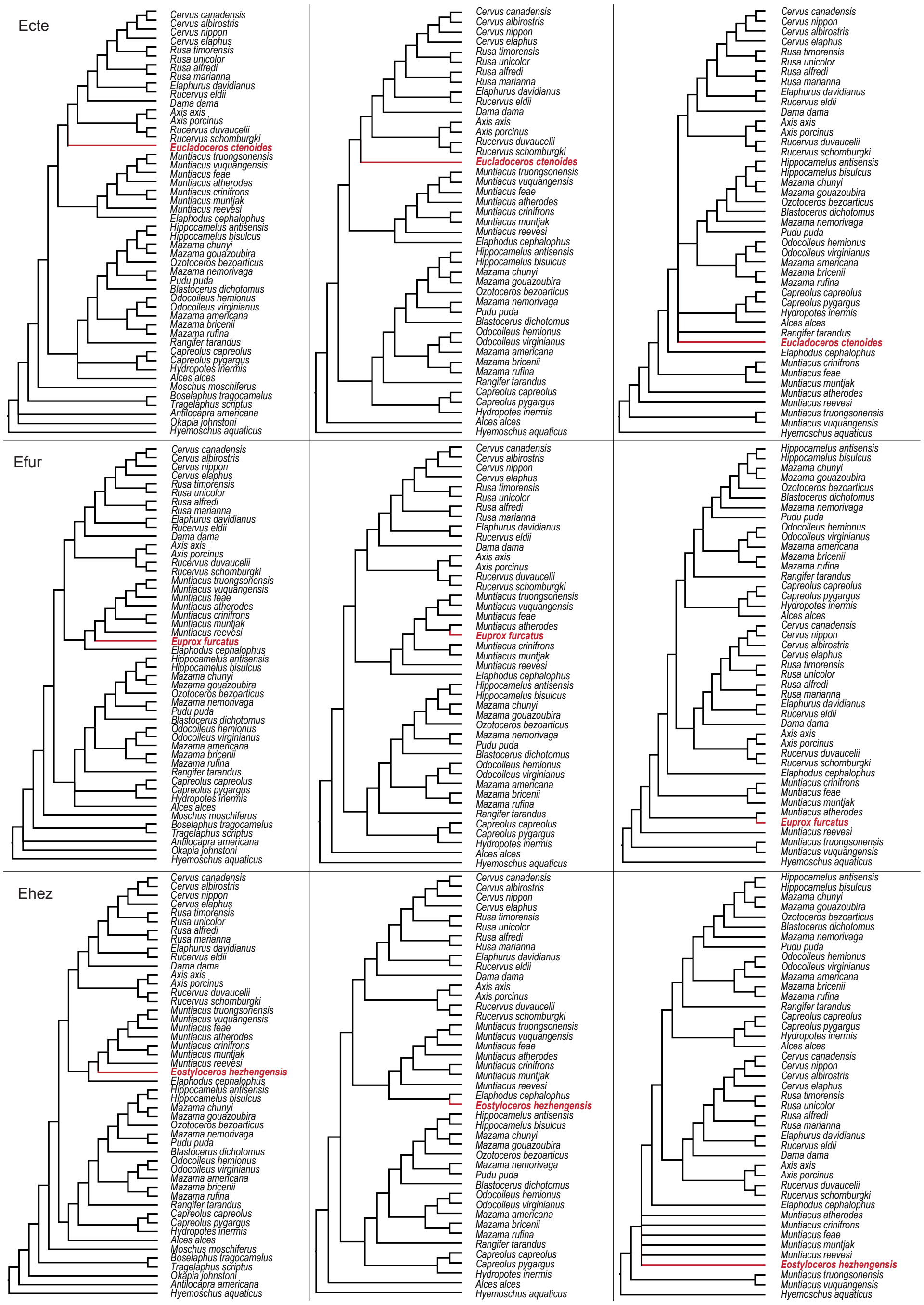

Figure 17: Overview of the three single fossil analyses for *Eucladoceros ctenoides*, *Euprox furcatus*, and *Eostyloceros hezhengensis*. Left: trees based on the supermatrix of the mitochondrial genome and the combined morphological data including outgroup taxa, middle: supermatrix excluding most outgroup taxa, right: backbone analyses.

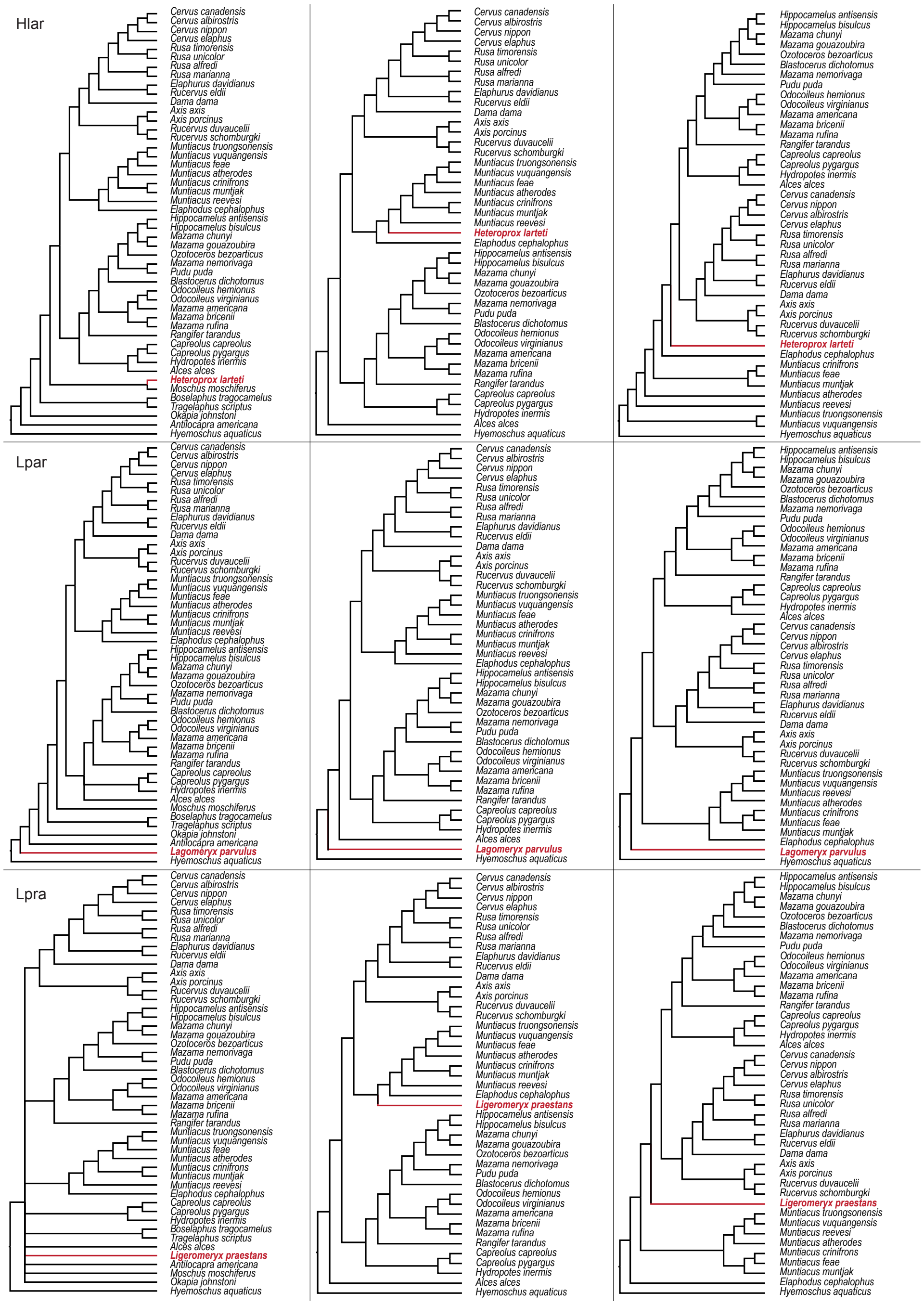

Figure 18: Overview of the three single fossil analyses for *Heteroprox larteti*, *Lagomeryx parvulus*, and *Ligeromeryx praestans*. Left: trees based on the supermatrix of the mitochondrial genome and the combined morphological data including outgroup taxa, middle: supermatrix excluding most outgroup taxa, right: backbone analyses.

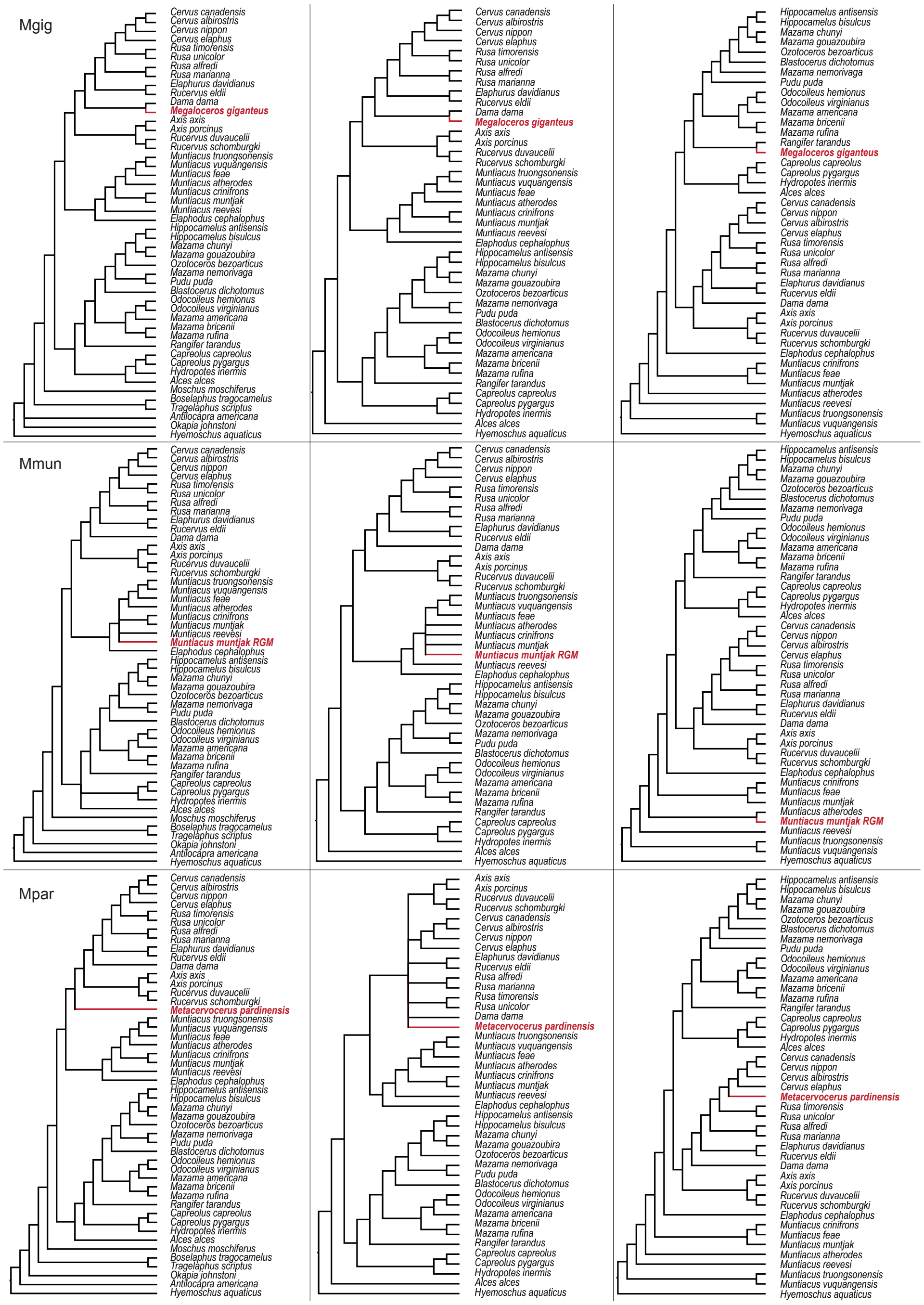

Figure 19: Overview of the three single fossil analyses for *Megaloceros giganteus*, *Muntiacus muntjak*, and *Metacervocerus pardinensis*. Left: trees based on the supermatrix of the mitochondrial genome and the combined morphological data including outgroup taxa, middle: supermatrix excluding most outgroup taxa, right: backbone analyses.

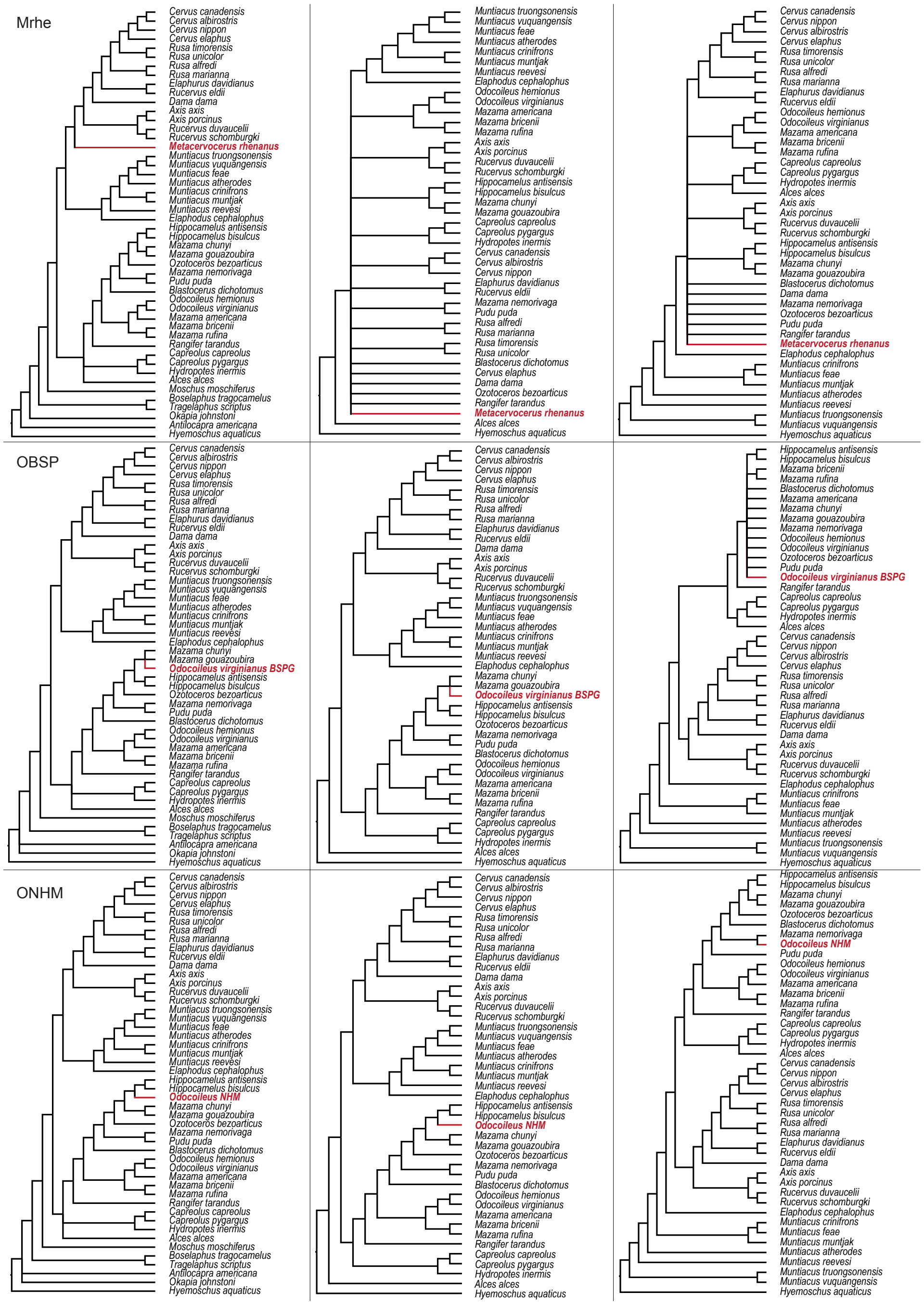

Figure 20: Overview of the three single fossil analyses for *Metacervocerus rhenanus*, *Odocoileus* (BSPG), and *Odocoileus* (NHM). Left: trees based on the supermatrix of the mitochondrial genome and the combined morphological data including outgroup taxa, middle: supermatrix excluding most outgroup taxa, right: backbone analyses.

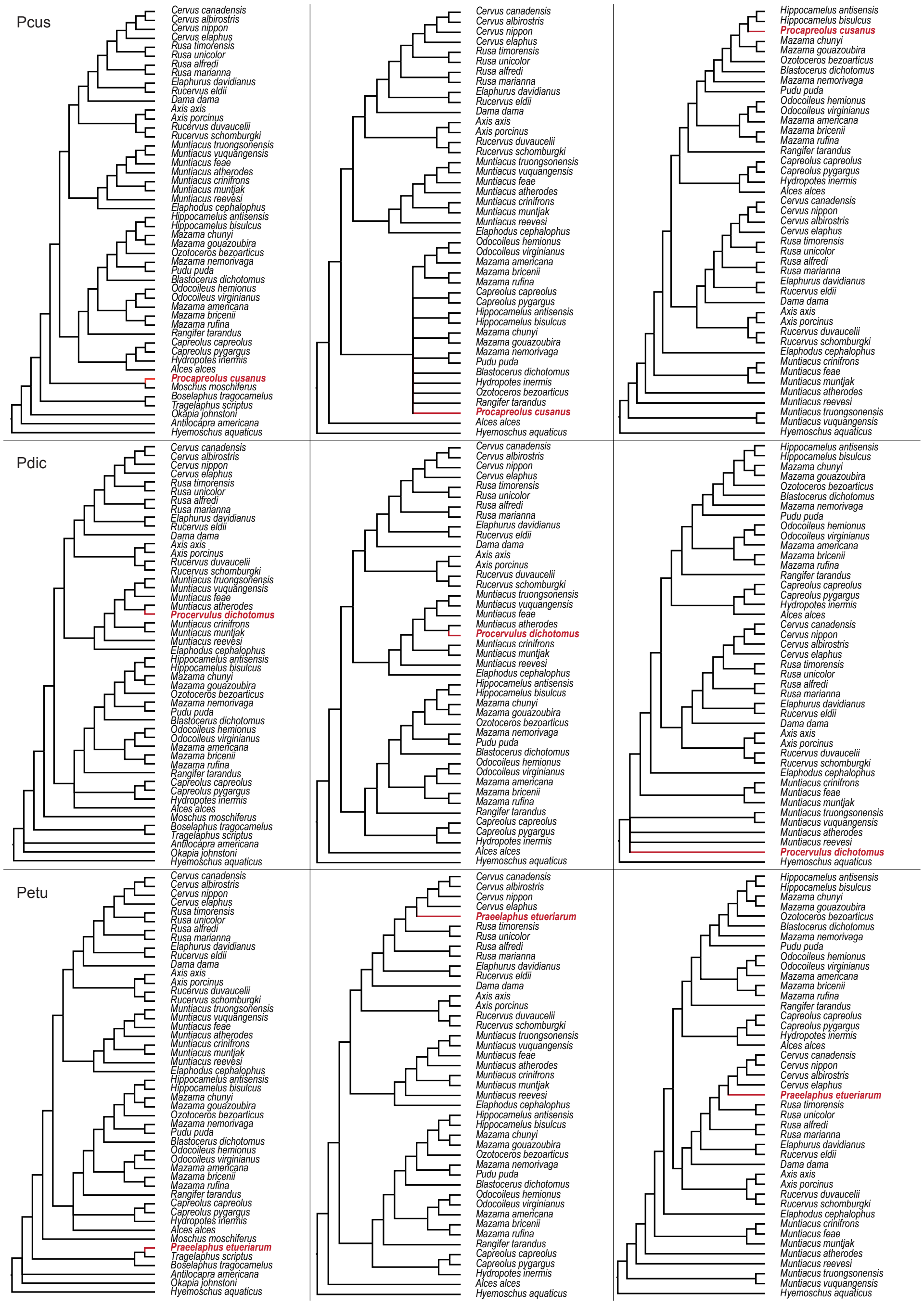

Figure 21: Overview of the three single fossil analyses for *Procapreolus cusanus*, *Procervulus dichotomus*, and *Praeelaphus etueriarum*. FLeft: trees based on the supermatrix of the mitochondrial genome and the combined morphological data including outgroup taxa, middle: supermatrix excluding most outgroup taxa, right: backbone analyses.

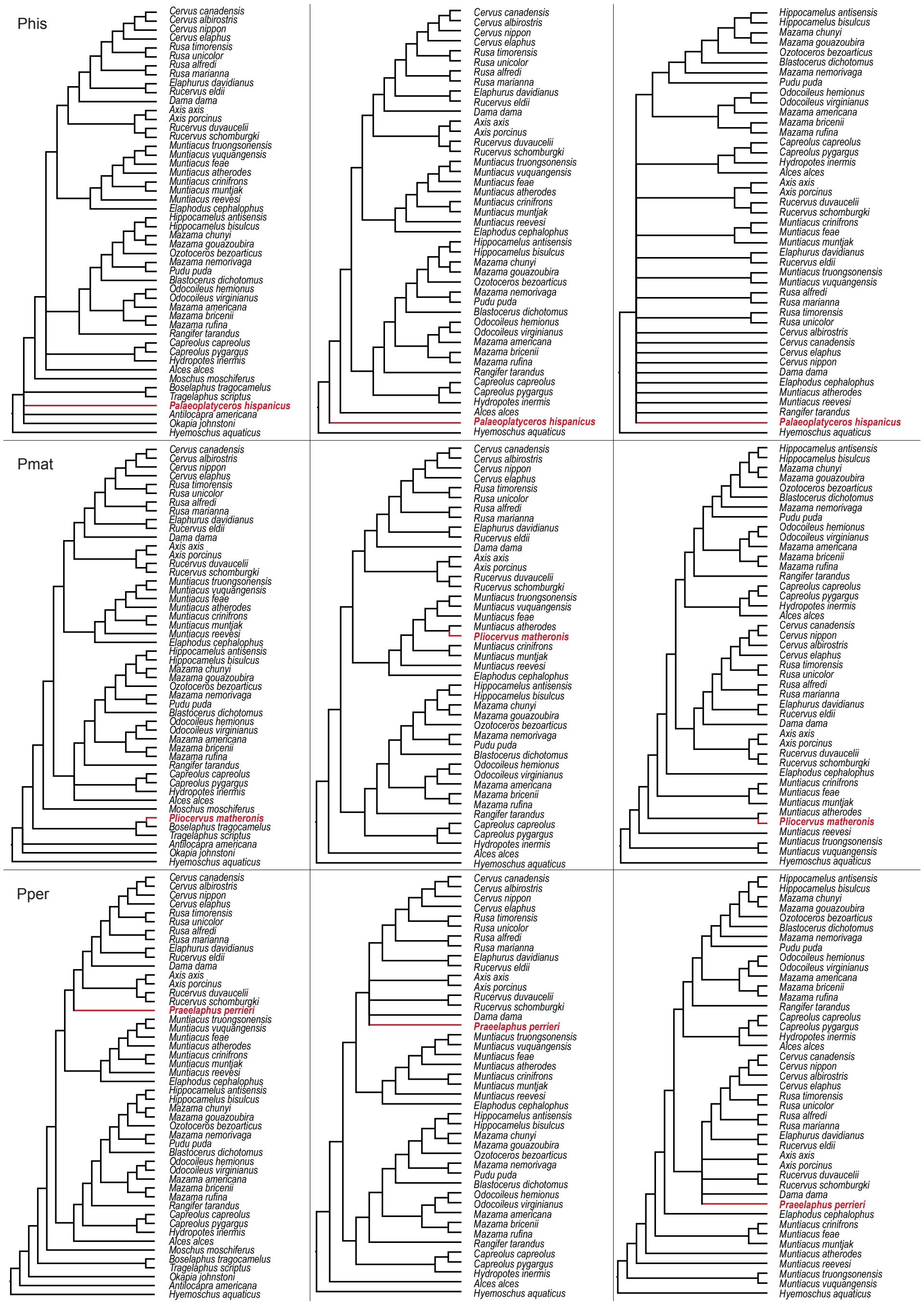

Figure 22: Overview of the three single fossil analyses for *Palaeoplatyceros hispanicus*, *Pliocervus matheronis*, and *Praeelaphus perrieri*. Left: trees based on the supermatrix of the mitochondrial genome and the combined morphological data including outgroup taxa, middle: supermatrix excluding most outgroup taxa, right: backbone analyses.

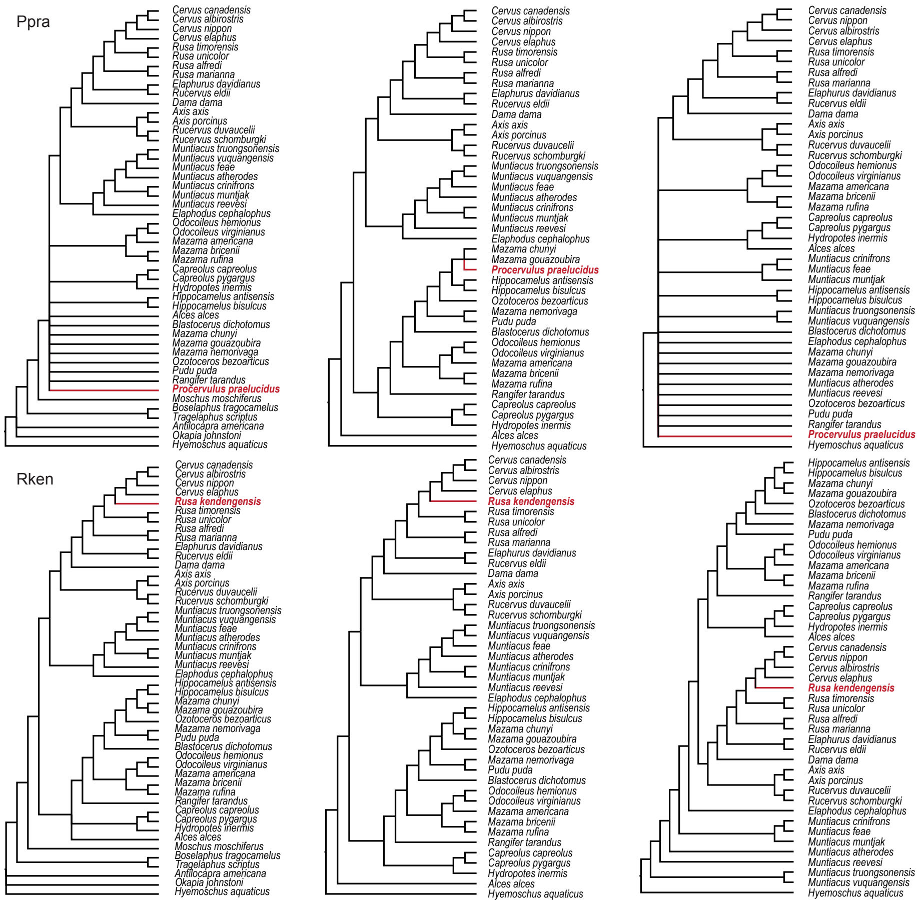

Figure 23: Overview of the three SFA for *Procervulus praelucidus* and *Rusa kendengensis*. Left: trees based on the supermatrix of the mitochondrial genome and the combined morphological data including outgroup taxa, middle: supermatrix excluding most outgroup taxa, right: backbone analyses.

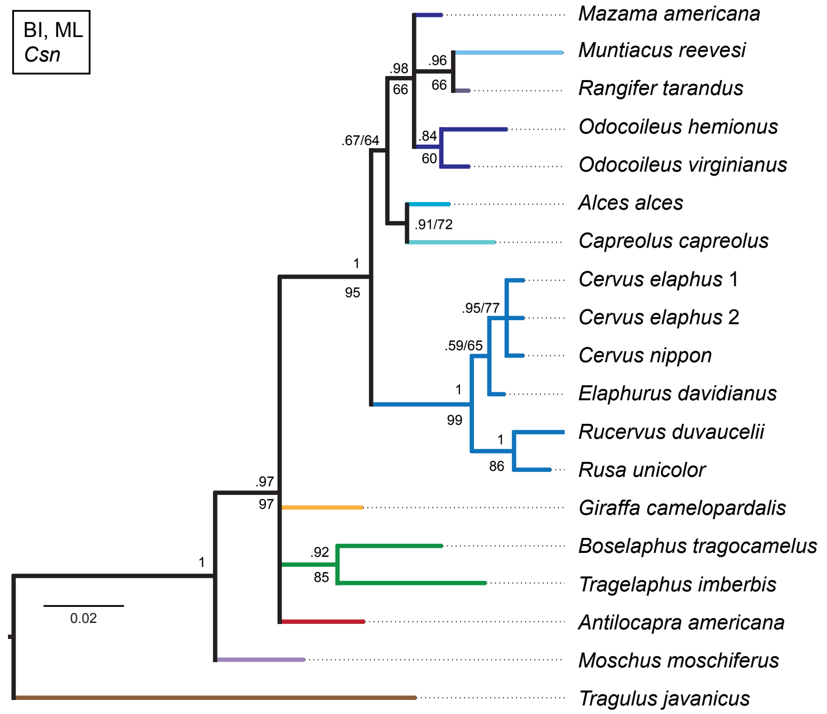

Figure 24: Bayesian consensus topology of the analysis of *Csn* including the posterior probabilities (above branches) and the ML bootstrap values (below branches) or as PP/BS.

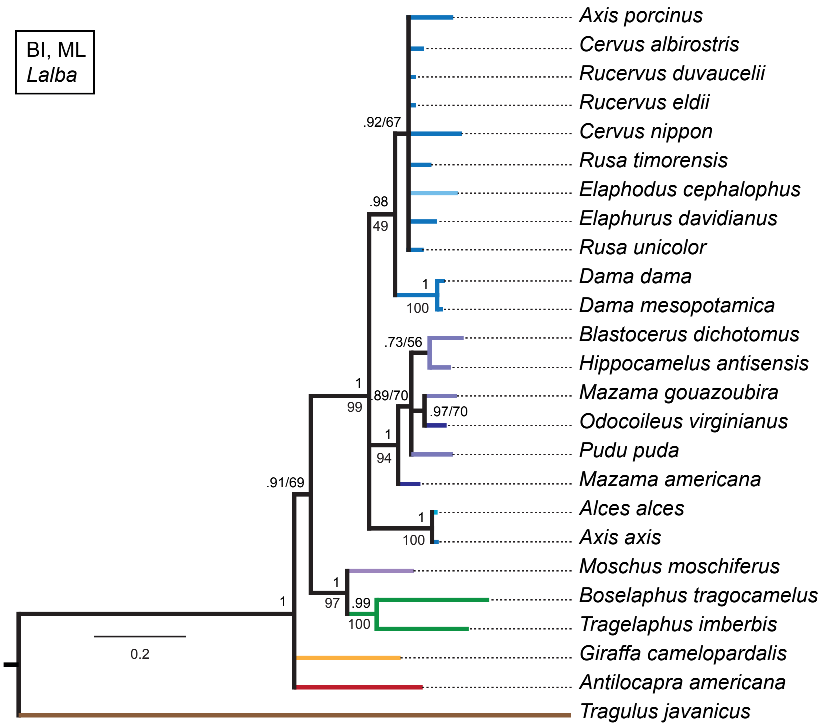

Figure 25: Bayesian consensus topology of the analysis of *Lalba* including the posterior probabilities (above branches) and the ML bootstrap values (below branches, or as PP/BS).

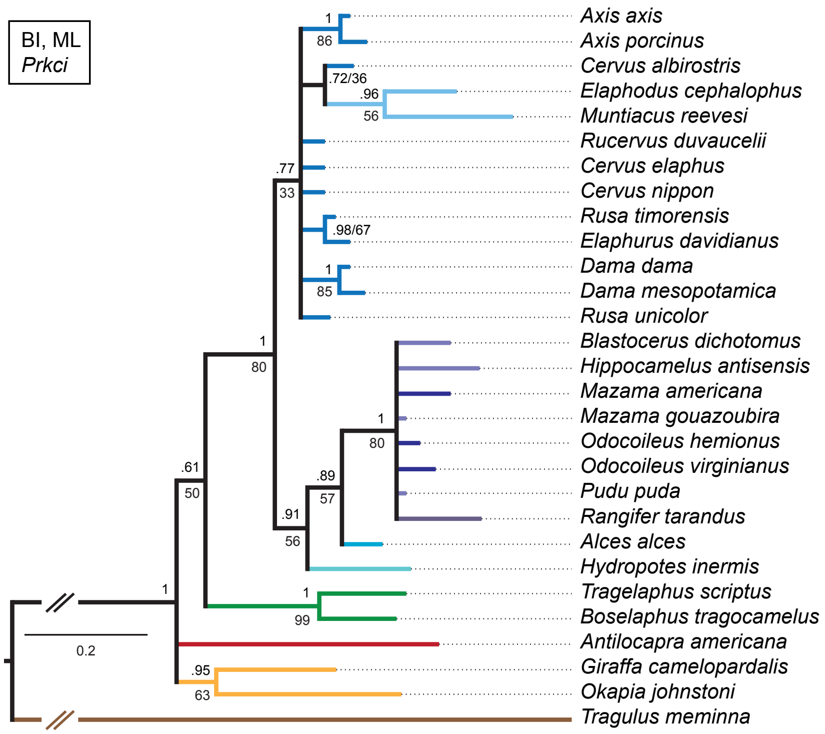

Figure 26: Bayesian consensus topology of the analysis of *Prkci* including the posterior probabilities (above branches) and the ML bootstrap values (below branches), or as PP/BS.

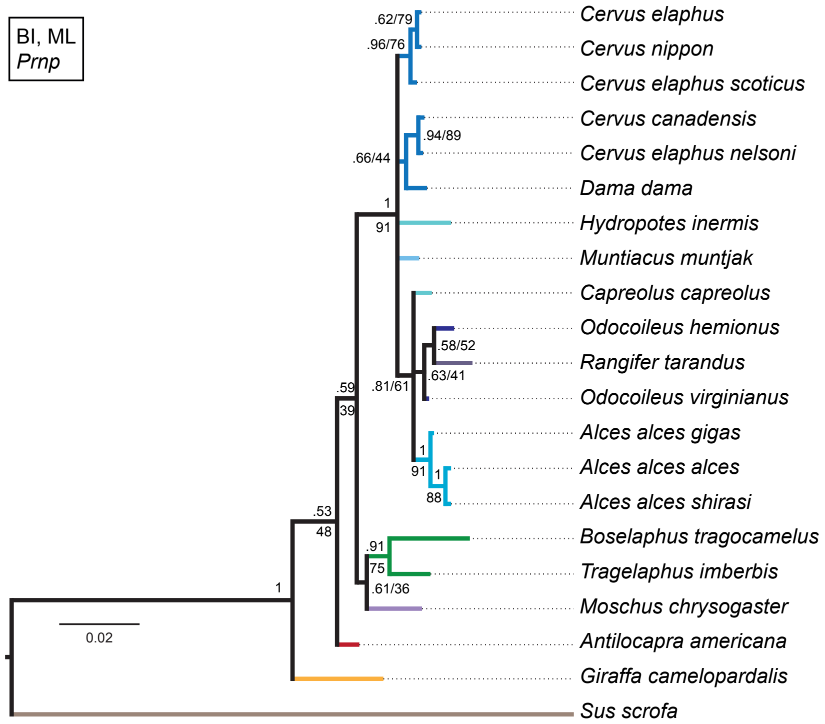

Figure 27: Bayesian consensus topology of the analysis of *Prnp* including the posterior probabilities (above branches) and the ML bootstrap values (below branches), or as PP/BS.

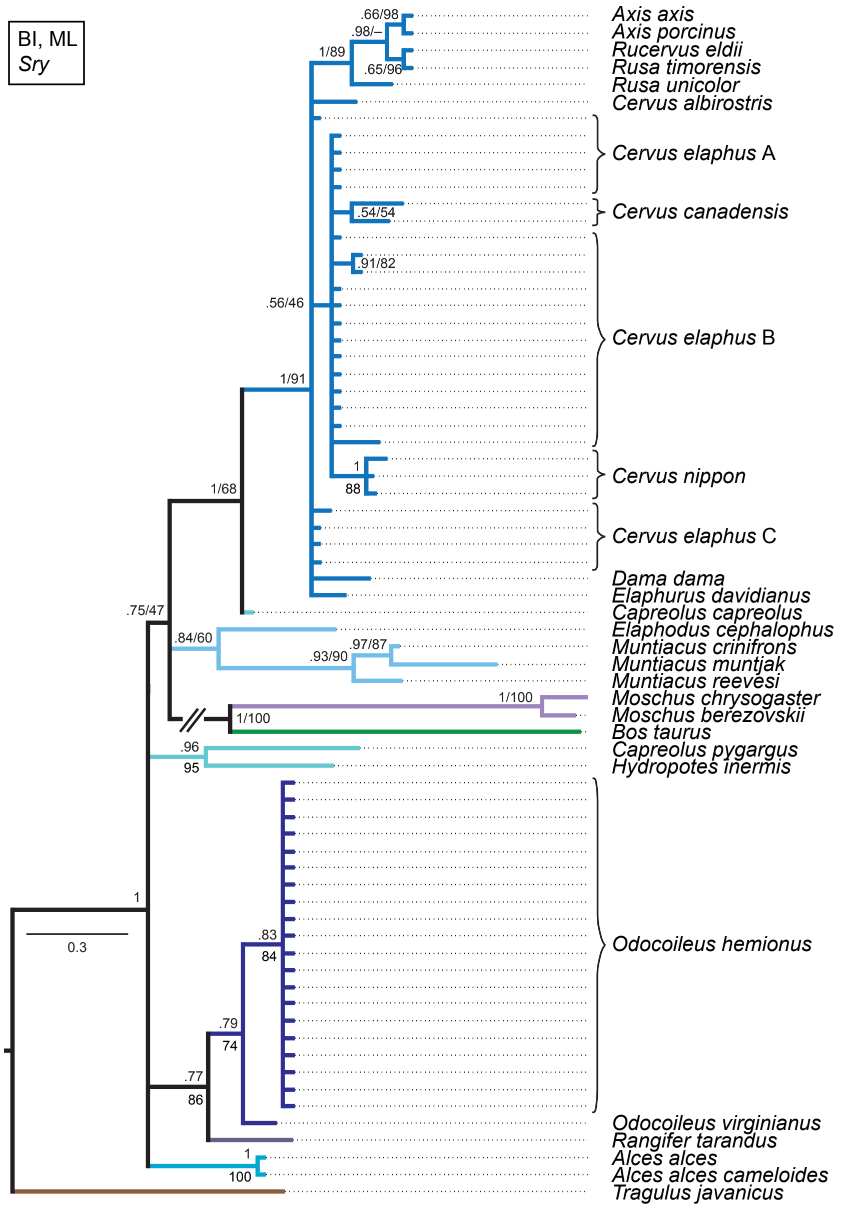

Figure 28: Bayesian consensus topology of the analysis of *Sry* including the posterior probabilities (above branches) and the ML bootstrap values (below branches) or as PP/BS.

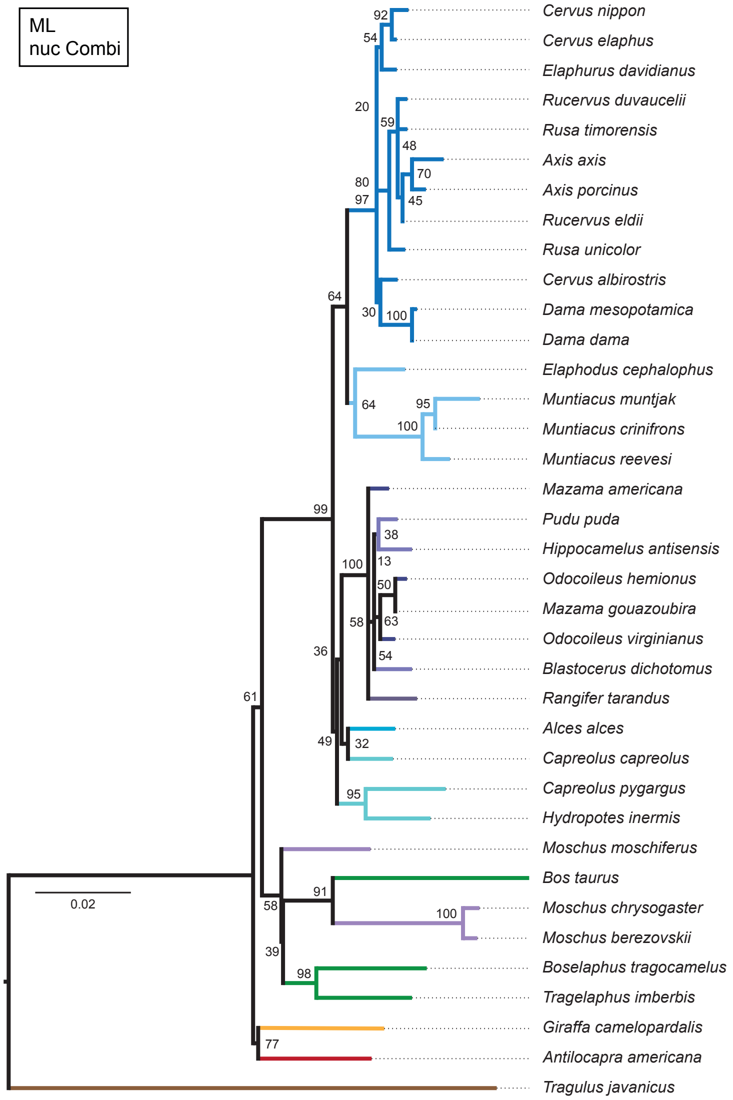

Figure 29: Maximum likelihood topology of the analysis combining five nuclear markers including bootstrap values.

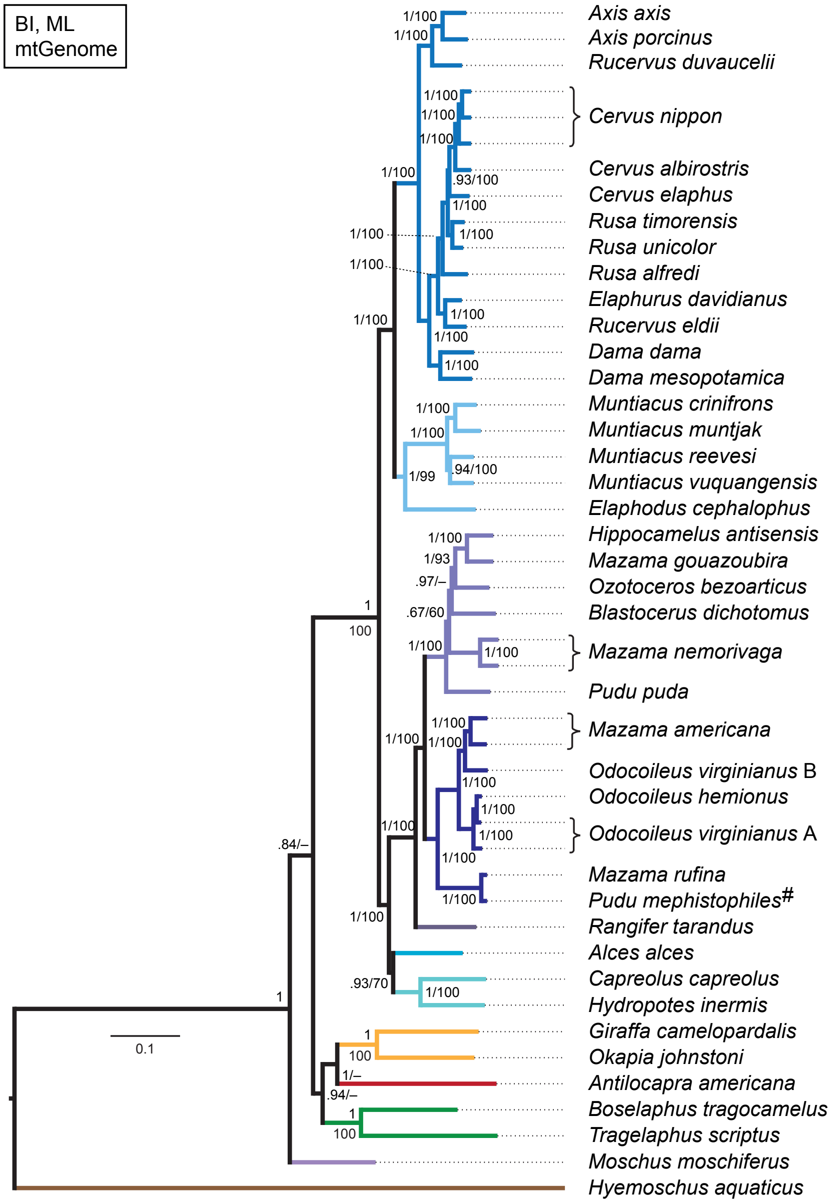

Figure 30: Bayesian consensus topology of the analysis of the complete mitochondrial genome including the posterior probabilities (above branches) and the ML bootstrap values (below branches) or as PP/BS. The misidentified *Pudu mephistophiles* sequence is indicated with #.

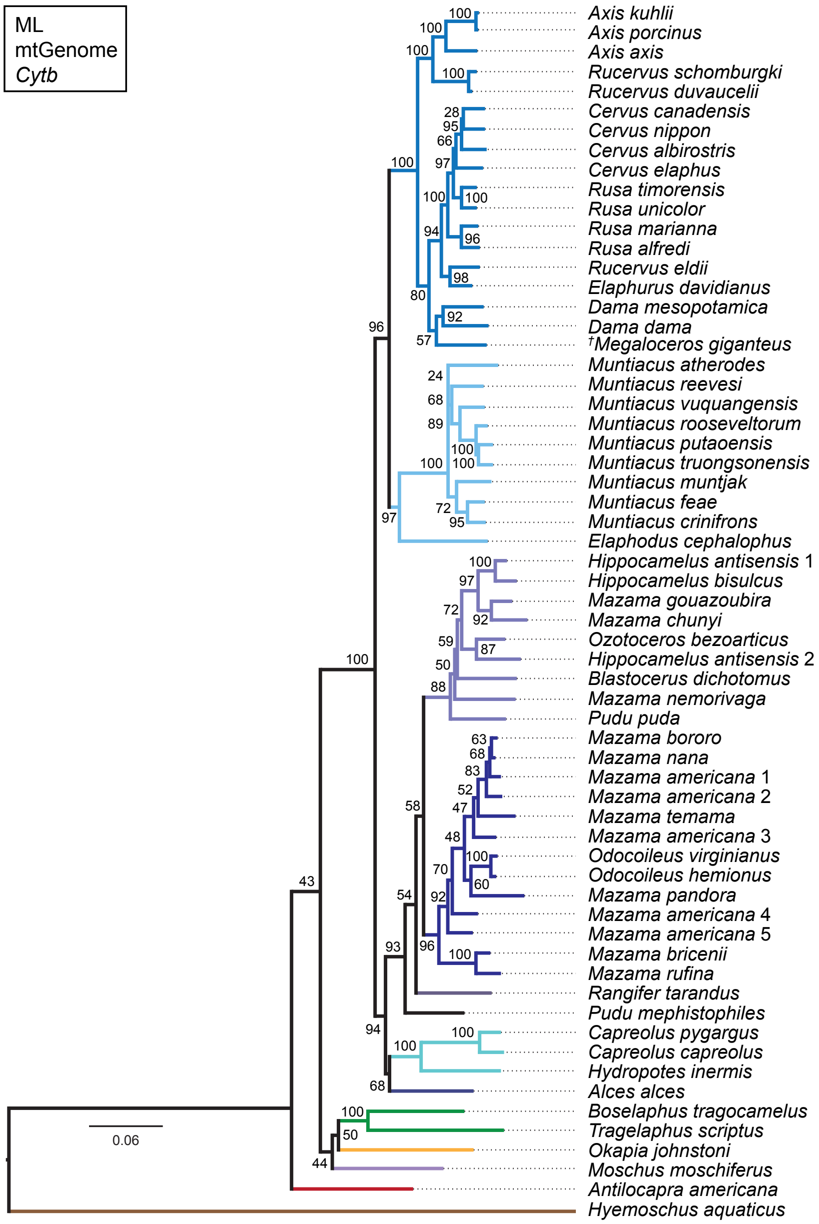

Figure 31: Best tree of the ML analysis of the complete mitochondrial genome including the *Cytb*-region with extended taxon sampling. The values represent bootstrap support.

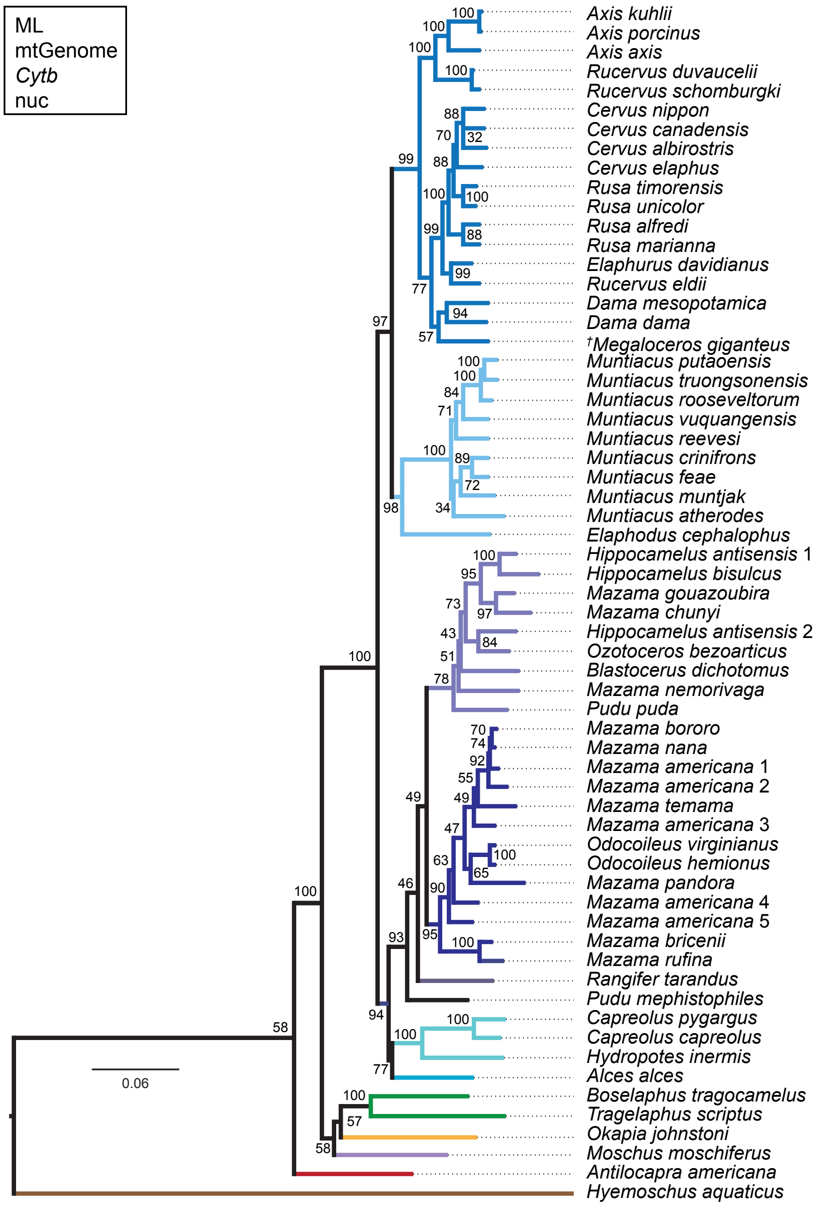

Figure 32: Best tree of the ML analysis of the combined nuclear and mitochondrial data set. The values represent bootstrap support.
